# PI3K/AKT/mTOR Axis in Cancer: From Pathogenesis to Treatment

**DOI:** 10.1002/mco2.70295

**Published:** 2025-07-30

**Authors:** Mingyang Jiang, Ke Zhang, Zhenwang Zhang, Xinran Zeng, Zihang Huang, Peizhuo Qin, Zhilin Xie, Xue Cai, Milad Ashrafizadeh, Yu Tian, Ruqiong Wei

**Affiliations:** ^1^ Department of Bone and Joint Surgery The First Affiliated Hospital of Guangxi Medical University Nanning China; ^2^ Hubei Key Laboratory of Diabetes and Angiopathy School of Pharmacy Xianning Medical College Hubei University of Science and Technology Xianning Hubei Province China; ^3^ Guangxi Medical University Nanning China; ^4^ Department of Psychosomatic Medicine The Huizhou Second People's Hospital Guangdong China; ^5^ Department of Radiation Oncology and Shandong Provincial Key Laboratory of Radiation Oncology Shandong Cancer Hospital and Institute Shandong First Medical University and Shandong Academy of Medical Sciences Jinan Shandong China; ^6^ Research Center the Huizhou Central People's Hospital Guangdong Medical University Huizhou Guangdong China; ^7^ Department of Rehabilitation Medicine The First Affiliated Hospital of Guangxi Medical University Nanning China

**Keywords:** cancer therapy, chemotherapy and immunotherapy, drug delivery, molecular pathway, PI3K/AKT/mTOR axis

## Abstract

The PI3K/AKT/mTOR signaling axis is a pivotal regulator of key cellular functions, including proliferation, metabolism, survival, and immune modulation. In cancer, its dysregulation drives malignant transformation, tumor progression, therapeutic resistance, and immune evasion. Despite numerous studies, an integrated understanding of this pathway's multifaceted role in tumor biology and treatment remains incomplete. This review comprehensively outlines the oncogenic mechanisms governed by the PI3K/AKT/mTOR pathway, including its regulation of epithelial–mesenchymal transition, autophagy, apoptosis, glycolysis, ferroptosis, and lipid metabolism. We emphasize the dual role of autophagy, its interplay with therapeutic resistance, and its contextual impact on cancer dynamics. Moreover, we explore the epigenetic regulation of this axis by noncoding RNAs (miRNAs, lncRNAs, circRNAs) and its influence on tumor hallmarks. The review also highlights the pathway's involvement in modulating responses to chemotherapy, radiotherapy, and immunotherapy, as well as its role in remodeling the tumor microenvironment. We critically evaluate emerging therapeutic strategies targeting the PI3K/AKT/mTOR axis, including small‐molecule inhibitors, phytochemicals, and nanoparticle‐based systems. By elucidating the integrated landscape of this pathway, our review highlights its value as a central therapeutic target and offers insights into precision oncology approaches aimed at overcoming drug resistance and enhancing treatment efficacy.

## Graphical abstract

The PI3K/AKT/mTOR signaling axis is crucial in cancer progression, regulating cell growth, survival, and metabolism. Often hyperactivated through mutations or loss of tumor suppressors such as PTEN, this pathway promotes cancer cell proliferation and survival by inhibiting apoptosis and activating mTOR, which drives protein synthesis and suppresses autophagy. The axis also contributes to therapy resistance, immune evasion, and metastasis. Targeting PI3K, AKT, or mTOR with inhibitors shows therapeutic potential, but overcoming resistance remains a challenge. Understanding its interaction with metabolic pathways like glycolysis and glutamine metabolism is key to developing effective treatments.

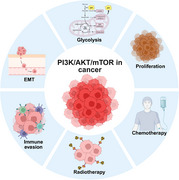

## 1 Introduction

The management and treatment of cancer remain major challenges in healthcare, despite significant advances in diagnostic and therapeutic tools. The American Cancer Society's 2025 Cancer Statistics [1] report projects over 2 million new cancer cases and approximately 618,120 cancer‐related fatalities in the United States for that year. Although overall cancer mortality has declined, saving 4.5 million lives since 1991 due to reduced smoking, early diagnosis, and effective therapies, significant racial disparities persist. Native American and Black communities continue to experience disproportionately higher death rates across various malignancies. Additionally, the cancer incidence rate among females, particularly middle‐aged and younger women, has increased, with elevated rates of lung and breast cancers. In contrast, there has been a decrease in cancer incidence among males. In 2021, the incidence rate of lung cancer among females under the age of 65 years was higher than that of males, indicating a shift in trends. Despite improvements in screening and treatment that have enhanced survival rates, especially for melanoma and leukemia, progress has been hindered by health disparities, inadequate preventive measures, and increasing cancer incidence in younger demographics. Addressing these issues requires improving access to healthcare and enhancing preventive measures.

In the recent years, research has focused on better understanding the biological profile of cancer, which is crucial for developing more effective therapeutics and overcoming carcinogenesis. Cancer development depends on genomic mutations that drive abnormal proliferation and another issue is related to the immune evasion [2, 3, 4, 5]. Additionally, tumor cells exhibit metastasis and spread to other parts of the body [6]. It has been shown that the metastatic rate of tumor cells can be influenced by transfer RNAs [7]. Research has also concentrated on elucidating oncogenic factors in tumors, including phosphoinositide 3‐kinase (PI3K)/protein kinase B (AKT)/mammalian target of rapamycin (mTOR), RAS/MAPK, and MYC [8]. Another key molecular factor is transforming growth factor‐β (TGF‐β), which controls cancer metastasis, stem cell features, and therapeutic resistance [9]. Therefore, dysregulation of molecular pathways is commonly observed in human cancers. Beyond molecular pathways, the roles of specific cells in tumorigenesis have been evaluated. For instance, neutrophils have been shown to trigger angiogenesis, metastasis, and immunosuppression [10]. Cancer cells’ response to reactive oxygen species (ROS) changes is complex and depends on various factors, including ROS types, levels, localization, persistence, origin, environment, and cancer stage [11]. Understanding the specific functions of each pathway in tumorigenesis can pave the way for cancer therapy, which is the focus of this review. Moreover, this review highlights the role of the PI3K/AKT/mTOR axis in carcinogenesis, with significant emphasis on cancer growth, metastasis, therapy resistance, and immune interactions. The PI3K/AKT/mTOR axis is among the most dysregulated pathways in cancers [12, 13, 14, 15, 16, 17], prompting studies to reveal new aspects of this pathway in tumorigenesis.

We describe the role of the PI3K/AKT/mTOR axis in regulating major hallmarks of cancer, including survival, metabolic reprogramming, angiogenesis, and immune evasion. We discuss the intricate molecular interactions and genomic alterations in this pathway, highlighting mutations in PIK3CA and phosphatase and tensin homolog (PTEN). We demonstrate the dual function of this pathway in regulating apoptosis, autophagy, and epithelial–mesenchymal transition (EMT), providing novel therapeutic targets. We also demonstrate the regulation of the PI3K/AKT/mTOR axis by small molecules, phytochemicals, and nanoparticles. Additionally, we cover the regulation of this axis by noncoding RNAs, a major step in the epigenetic regulation of human cancers.

## 2 PI3K/AKT/mTOR

### 2.1 Structural Components and Genetic Alterations of the PI3K/AKT/mTOR Pathway

**FIGURE 1:**
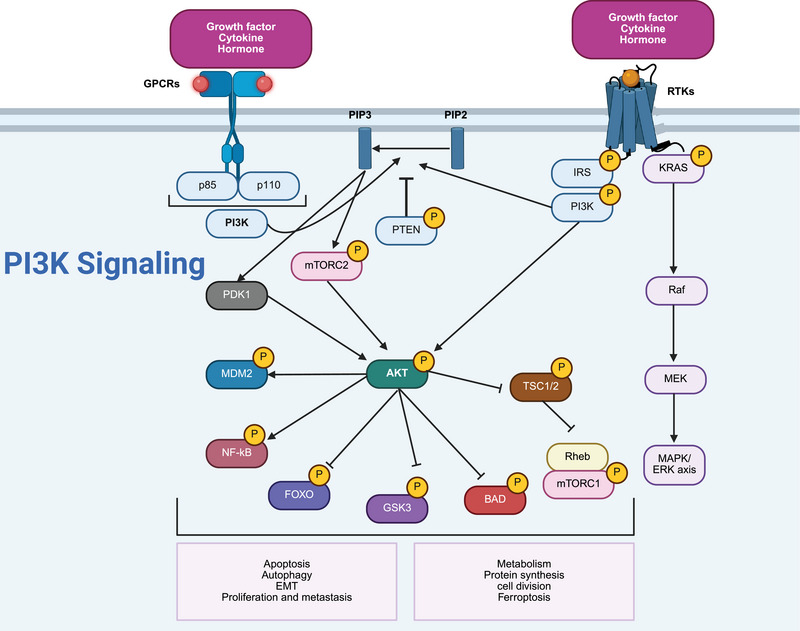
A schematic representation of PI3K/AKT/mTOR axis (adapted with permission from Ref. [25]). Two receptors including RTKs and GPCRs exert a significant function in the induction of PI3K. GPCRs can interact with p85 and p110 subunits of PI3K to elevate the generation of PIP3. A similar strategy is followed by RTKs interacting with IRS and PI3K to trigger PIP3 generation. PTEN is a negative regulator through suppressing PIP2 conversion to PIP3. The generated PIP3 stimulates PDK1 and mTORC2 to enhance levels of AKT. On the other hand, activated AKT enhances the levels of MDM2 and NF‐kB, while it downregulates FOXO, GSK3, Bad and TSC1/2. The stimulation of PI3K/AKT/mTOR axis has been demonstrated in the various cancers [26, 27, 28]. AKT, protein kinase B; BAD, Bcl‐2‐associated death promoter; FOXO, Forkhead box O; GPCRs, G protein‐coupled receptors; GSK3, glycogen synthase kinase 3; IRS, insulin receptor substrate; KRAS, Kirsten rat sarcoma viral oncogene homolog; MAPK/ERK, mitogen‐activated protein kinase/extracellular signal‐regulated kinase; MDM2, mouse double minute 2 homolog; MEK, mitogen‐activated protein kinase kinase; mTORC1/2, mechanistic target of rapamycin complex 1/2; NF‐κB, nuclear factor kappa‐light‐chain‐enhancer of activated B cells; PDK1, 3‐phosphoinositide‐dependent protein kinase‐1; PI3K, phosphoinositide 3‐kinase; PIP2, phosphatidylinositol 4,5‐bisphosphate; PIP3, phosphatidylinositol 3,4,5‐trisphosphate; PTEN, phosphatase and tensin homolog; Raf, rapidly accelerated fibrosarcoma kinase; Rheb, Ras homolog enriched in brain; RTKs, Receptor tyrosine kinases; TSC1/2, tuberous sclerosis complex 1 and 2; (created with Biorender.com).

**FIGURE 2:**
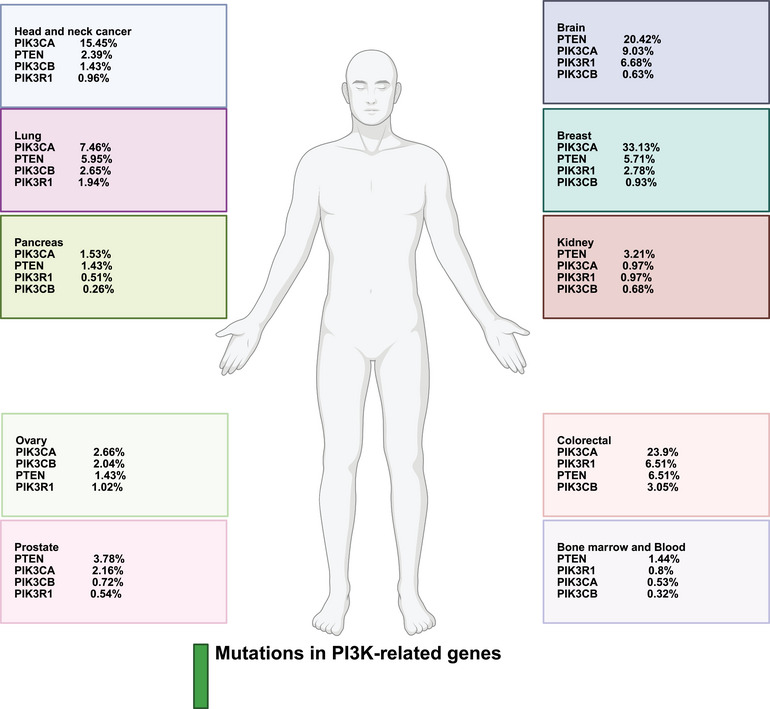
Mutations in the PI3K‐related genes (adapted with permission from Ref. [29]). PIK3CA, phosphatidylinositol‐4,5‐bisphosphate 3‐kinase catalytic subunit alpha; PIK3CB, phosphatidylinositol‐4,5‐bisphosphate 3‐kinase catalytic subunit beta; PIK3R1, phosphoinositide‐3‐kinase regulatory subunit 1; PTEN, phosphatase and tensin homolog; (created with Biorender.com).

**FIGURE 3:**
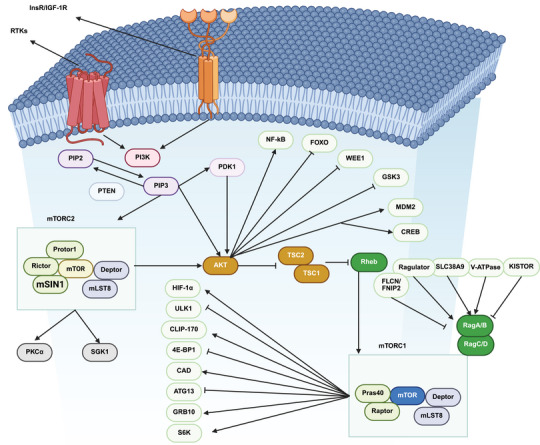
The regulation of PI3K/AKT/mTOR at the RNA and DNA levels. In response to growth factors or cytokines, receptor tyrosine kinases (RTKs) are activated and attract PI3K to the plasma membrane. At the membrane, PI3K catalyzes the conversion of PI(4,5)P2 to PI(3,4,5)P3, which then activates AKT via PDK1 and mTORC2. Activated AKT promotes several cellular functions, such as cell cycle progression, proliferation, apoptosis suppression, glucose metabolism, cell survival, actin cytoskeleton structure, transcription, motility, and movement. It accomplishes this by phosphorylating downstream targets like TSC2, so alleviating TSC2‐induced inhibition of Rheb and facilitating mTORC1 activation at the lysosome. Moreover, mTORC1 activity is modulated by amino acids through distinct sensors such as regulator, V‐ATPase, and notably Rags, which position mTORC1 at the lysosome. Upon activation, mTORC1 stimulates protein synthesis, increases nucleotide production, and suppresses autophagy and proteasomal degradation (adapted with permission from Ref. [30]). 4E‐BP1, eukaryotic translation initiation factor 4E‐binding protein 1; AKT, protein kinase B; ATG13, autophagy related 13; CAD, carbamoyl‐phosphate synthetase 2, aspartate transcarbamylase, and dihydroorotase; CLIP‐170, cytoplasmic linker protein of 170 kDa; CREB, cAMP response element‐binding protein; Deptor, DEP domain containing MTOR‐interacting protein; FLCN/FNIP2, folliculin/folliculin interacting protein 2; FOXO, Forkhead Box O; GRB10, growth factor receptor‐bound protein 10; GSK3, glycogen synthase kinase 3; HIF‐1α, hypoxia‐inducible factor 1‐alpha; IGF‐1R, insulin‐like growth factor 1 receptor; InsR, insulin receptor; KISTOR, Kidney‐specific TOR‐interacting protein; MDM2, mouse double minute 2 homolog; mLST8, mammalian lethal with SEC13 protein 8; mTORC1, mechanistic target of rapamycin complex 1; mTORC2, mechanistic target of rapamycin complex 2; NF‐κB, nuclear factor kappa‐light‐chain‐enhancer of activated B cells; PDK1, 3‐phosphoinositide‐dependent protein kinase‐1; PI3K, phosphoinositide 3‐kinase; PIP2, phosphatidylinositol 4,5‐bisphosphate; PIP3, phosphatidylinositol 3,4,5‐trisphosphate; PKCα, protein kinase C alpha; Pras40, proline‐rich AKT substrate of 40 kDa; PTEN, phosphatase and tensin homolog; RagA/B/C/D, Ras‐related GTP‐binding proteins; Raptor, regulatory associated protein of mTOR; Rheb, Ras homolog enriched in brain; Rictor, rapamycin‐insensitive companion of mTOR; RTKs, receptor tyrosine kinases; S6K, ribosomal protein S6 kinase; SGK1, serum and glucocorticoid regulated kinase 1; SLC38A9, solute carrier family 38 member 9; TSC1/2, tuberous sclerosis complex 1 and 2; ULK1, Unc‐51 like autophagy activating kinase 1; V‐ATPase, vacuolar‐type H+‐ATPase; WEE1, WEE1 G2 checkpoint kinase; (created with Biorender.com).

The human genome has been demonstrated to encode three classes of intracranial aneurysms (IA) p110 isoforms, including p110α (encoded by PIK3CA) and p110β (PIK3CB), demonstrating expression in various cell types, and p110δ (PIK3CD), with specific expression in immune cells [18]. There are five class IA PI3K regulatory subunit isoforms and splice variants in humans, including p85α, p85β, p55γ, p55α, and p50α, with p85α being the most well known and encoded by PIK3R1. The class IB PI3K catalytic subunit is p110γ (PIK3CG), capable of binding to the regulatory subunits p101 or p87 [18]. The function of each class of PI3K is determined based on its significance in cell signaling (class I and II) or membrane trafficking (class II and III). The class IA PI3Ks, and specifically the p110α isoform, demonstrate significant function in human cancers as part of the PI3K axis. Various cancers have demonstrated mutations or amplifications in the PIK3CA gene [19]. Suppression of the p110α isoform has elevated carcinogenesis in breast tumors [20]. The modulatory roles of other class IA isoforms, including p110β and p110δ, have been demonstrated in preclinical studies [20, 21]. Notably, a small fraction of class I PI3K activity is required to preserve cell survival and proliferation [22, 23]. On the other hand, mTOR is considered as a conserved serine/threonine kinase with significant function in regulating growth and metabolism. A number of upstream factors, including the human epidermal growth factor receptor, insulin‐like growth factor 1 (IGF‐1), and vascular endothelial growth factor (VEGF), can regulate mTOR and are known as positive mediators. Conversely, PTEN, tuberous sclerosis complex 1 and 2 (TSC1/2), and liver kinase B1 (LKB1) are considered negative regulators of mTOR. The mTORC1 and mTORC2 are two complexes of mTOR, with mTORC1 being rapamycin‐sensitive and responding to energy, nutrient, and stress sensing to enhance protein synthesis and cellular growth by targeting 4E‐BP1 and S6K. Dysregulation of the PI3K/AKT/mTOR axis has been observed in human cancers, increasing proliferation and metastasis. Therefore, mTOR inhibitors, especially those targeting mTORC1, have been developed to treat cancers [24]. Figure 1 demonstrates the typical PI3K/AKT/mTOR axis, also summarized in Table 1. Figure 2 demonstrates the genes with frequent mutations in human cancers related to PI3K. Figure 3 demonstrates the regulation and interaction of PI3K/AKT at the RNA and DNA levels.

**TABLE 1:** A summary of the role of PI3K/AKT/mTOR axis in cancer progression.

Cancer	Remark	References
Lung cancer	Mex3a demonstrates interaction with LAMA2 to trigger PI3K/AKT axis in metastasis	[31]
Bladder cancer	ARID1A‐deficient cells demonstrate reliance on PI3K axis and it has sensitivity to inhibitors of EZH2 and PI3K	[32]
Gastric cancer	WIPF1 enhances PI3K/AKT expression Myocardin increases WIPF1 expression	[33]
Gastric cancer	PAX3 stimulates proliferation and angiogenesis through enhancing VEGF, PI3K, and MET	[34]
Prostate cancer	Suppressing HDAC and PI3K can impair phenotypic heterogeneity	[35]
Breast cancer	PRR14 stimulates PI3K and suppresses CHEK2 in enhancing tumorigenesis	[36]
Lung cancer	TIM‐4 mediates mitochondrial homeostasis and enhances lung tumor progression via L‐OPA1 by PI3K/AKT overexpression	[37]
–	Upregulation of PI3K enhances immune evasion through increasing an inhibitory myeloid TME	[38]
–	Upregulation of PAK and PI3K triggers sorafenib resistance	[39]

Abbreviations: AKT, protein kinase B; ARID1A, AT‐rich interaction domain 1A; CHEK2, checkpoint kinase 2; EZH2, enhancer of Zeste homolog 2; HDAC, histone deacetylase; L‐OPA1, long isoform of optic atrophy 1; LAMA2, laminin subunit alpha 2; MET, mesenchymal–epithelial transition factor; NLRP6, NOD‐like receptor family pyrin domain containing 6; p85α, regulatory subunit alpha of PI3K; PAK, p21‐activated kinase; PAX3, paired box gene 3; PI3K, phosphoinositide 3‐kinase; PRR14, proline‐rich protein 14; TIM‐4, T‐cell immunoglobulin and mucin‐domain containing‐4; VEGF, vascular endothelial growth factor; WIPF1, WASP interacting protein family member 1.

### 2.2 Activation and Oncogenic Role of the PI3K/AKT/mTOR Axis

One of the commonly activated pathways in human cancers is the PI3K/AKT axis [40, 41, 42]. The stimulation of the PI3K/AKT axis under physiological conditions can occur as a result of induction by insulin, growth factors, and cytokines. This axis participates in the regulation of important metabolic mechanisms, such as glucose metabolism, macromolecule synthesis, and redox balance modulation. The induction of PI3K in human cancers causes metabolic reprogramming by enhancing the function of nutrient transporters and metabolic enzymes, to meet the demands of growing cells. By highlighting the function of the PI3K/AKT axis in metabolism in normal cells and how such regulation occurs in tumor cells, the metabolic vulnerabilities can be identified [43]. The major hallmarks of cancer, including survival, metastasis, proliferation, metabolism, and therapy resistance, can be regulated by PI3K/AKT. PI3K can regulate the tumor microenvironment (TME) and control a number of important mechanisms, including angiogenesis and inflammatory factor recruitment. Genomic alterations, such as PIK3CA mutation, PTEN, AKT, RAS1, and mTOR, can modulate this axis [44, 45]. The function of PI3K is related to PIP2 phosphorylation, to accelerate the generation of PIP3. Then, PIP3 participates in the recruitment of proteins, including AKT [46]. Upon stimulation, AKT contributes to the phosphorylation of substrates, and mTOR is considered the most well‐known downstream target of AKT, contributing to protein synthesis to enhance tumorigenesis. The components of the PI3K/AKT/mTOR axis have demonstrated mutations and activation in various tumors [45, 47, 48]. The class I PI3Ks are considered heterodimeric lipid kinases, comprised of a p110 catalytic subunit and a regulatory subunit [18, 49].

### 2.3 PI3K/AKT/mTOR and Biological Mechanisms

#### 2.3.1 PI3K/AKT/mTOR and EMT

##### 2.3.1.1 PI3K/AKT/mTOR Axis in EMT‐Mediated Cancer Progression

EMT is a flexible and dynamic process involving phenotype transformation and changes in biochemical factors. The phenotype changes are based on the transformation of epithelial cells to mesenchymal cells, along with the loss of apical–basal polarity and cell–cell adhesion features. The biochemical alterations involve an increase in the levels of Snail, Slug, zinc‐finger E‐box‐binding homeobox 1 (ZEB)Polymerase (RNA) III (DNA directed) polypeptide G, and TWIST, which are capable of inducing EMT. Despite its critical role in physiological processes such as embryogenesis and wound healing, the role of EMT in cancer has achieved significant interest. Increasing evidence has highlighted the role of EMT in cancer metastasis and drug resistance [50, 51, 52, 53, 54, 55], which is the focus of this section on the interaction between PI3K/AKT and EMT.

Understanding the mechanisms driving peritoneal metastasis is crucial in the field of gastric cancer (GC), as it is a key factor in poor prognosis. Elevated expression of apolipoprotein C‐II (APOC2) is associated with enhanced EMT, increased cellular migration, invasion, and proliferation, mediated through activation of the PI3K/AKT/mTOR cascade. APOC2 was found to interact with CD36, a lipid metabolism receptor, forming an axis promoting EMT and peritoneal dissemination of GC cells. Disruption of either APOC2 or CD36 significantly suppressed these malignant behaviors in vitro and in murine models, whereas pharmacological modulation of the PI3K/AKT/mTOR pathway reversed these effects. These findings suggest the APOC2–CD36 axis as a potential therapeutic target for preventing aggressive GC progression [56]. In addition to GC, PI3K/AKT activity has been linked to the aggressiveness of bladder cancer. A significant upregulation of polymerase (RNA) III (DNA directed) polypeptide G (POLR3G)Glutathione peroxidase 2 was identified in advanced bladder cancer tissues, correlating with aggressive tumor behavior and reduced survival. Suppression of POLR3G expression reduced cellular migration and invasion and reversed mesenchymal marker expression, highlighting its pivotal role in EMT regulation. Mechanistically, POLR3G activates the PI3K/AKT pathway. Functional assays further demonstrated that the prometastatic effects of POLR3G were mediated through this pathway, both in vitro and in vivo, establishing POLR3G as a critical effector of EMT and metastasis in bladder cancer [57]. However, the upstream regulatory mechanisms of POLR3G expression and its interactions with other signaling pathways in bladder cancer remain unclear and warrant further investigation. Future studies should explore the therapeutic potential of targeting POLR3G in combination with PI3K/AKT inhibitors to assess synergistic effects in preventing EMT and metastasis.

The function of EMT in promoting cancer metastasis is not specific to any one type of cancer; therefore, in cases of PI3K/AKT upregulation, EMT induction may drive metastasis across various tumors. This highlights the importance of targeting EMT‐related pathways as a promising strategy to limit metastatic progression across different cancer types. Therefore, therapeutic targeting of EMT is a promising strategy for suppressing cancer metastasis. Carboxypeptidase A4 (CPA4) has been identified as a regulator of cancer progression and serves as a reliable biomarker in various tumors. CPA4 has oncogenic functions in pancreatic cancer and is directly associated with tumor size, stage, and lymph node (LN) metastasis. Its contribution to metastasis is linked to the activation of the PI3K/AKT/mTOR signaling axis, a critical mediator of EMT. CPA4 stimulates PI3K/AKT/mTOR to trigger EMT‐related metastasis [58]. Similarly, glutathione peroxidase 2 (GPX2) has been shown to promote EMT and metastasis in NSCLC via modulation of the same pathway. GPX2 was identified as a critical regulator of NSCLC progression and metastasis. Elevated GPX2 expression correlates with poor prognosis and is associated with larger tumor size, LN metastasis, and higher TNM stage. Mechanistically, GPX2 enhances EMT, as evidenced by reduced E‐cadherin and increased vimentin and Snail levels. Functionally, GPX2 promotes NSCLC cell migration and invasion in vitro and lung metastasis in vivo. These effects are mediated through ROS reduction and activation of the PI3K/AKT/mTOR/Snail pathway, highlighting GPX2 as a potential diagnostic biomarker and therapeutic target in NSCLC [59]. Further supporting this mechanism, sodium bicarbonate cotransporter 3 (SLC4A7)PAK5 (P21 (RAC1) Activated Kinase 5) has been implicated in EMT induction and metastatic behavior in head and neck squamous cell carcinoma (HNSCC). SLC4A7 was found to be upregulated in HNSCC and associated with poor prognosis. Its expression enhances cell migration, invasion, and EMT without significantly affecting proliferation. Mechanistically, SLC4A7 activates the PI3K/AKT/mTOR pathway, leading to the increased expression of mesenchymal markers and the EMT‐TF SNAI2. These effects were reversed by GDC‐0980, confirming pathway involvement. In vivo, SLC4A7 promotes lung metastasis in a tail vein model, reinforcing its role in tumor progression. Overall, SLC4A7 functions as a key regulator of EMT and metastasis in HNSCC via PI3K/AKT/mTOR [60]. According to these studies, the major focus has been on the role of PI3K/AKT in EMT regulation in solid tumors. However, hematological tumors are also among the most malignant cancers worldwide [61, 62] and therefore, EMT induction by PI3K/AKT in these tumors requires significant research to further develop novel therapeutics.

In cancer biology, the mechanisms underlying tumor cell migration, invasion, and metastasis are critically influenced by molecular pathways regulating EMT. One such regulatory molecule is PAK5 (P21 (RAC1) Activated Kinase 5) (PAK5)F‐box protein 11, whose role in EMT and metastasis has been extensively studied in ovarian cancer. Elevated expression of PAK5 has been observed in ovarian cancer tissues, particularly in cases with distant metastasis, correlating with poorer patient survival. When PAK5 was suppressed, cancer cells adopted epithelial characteristics, showed reduced motility, and exhibited downregulation of mesenchymal markers. Conversely, overexpression of PAK5 led to enhanced mesenchymal traits and significantly increased cell migration and invasion. These cellular changes were mechanistically linked to the activation of the PI3K/AKT pathway, as evidenced by decreased phosphorylation of pathway components upon PAK5 knockdown. Thus, the oncogenic potential of PAK5 has been established through its role in promoting EMT and enhancing metastatic behaviors via PI3K/AKT pathway activation [63]. Consistent with these findings, another EMT‐promoting factor, F‐box protein 11 (FBXO11), has shown similar oncogenic behavior, particularly in GC. Increased expression of FBXO11 has been associated with enhanced tumor aggressiveness, including larger tumor size, LN metastasis, and advanced clinical stage. Poor overall survival has been linked to its upregulation. Cellular experiments have demonstrated that silencing FBXO11 reduces proliferation, migration, and invasion of GC cells, while its overexpression promotes these processes. EMT processes are facilitated through FBXO11 activity, alongside activation of the PI3K/AKT pathway. This activation appears to be mediated, at least in part, through the downregulation of PTEN, a known tumor suppressor. Pharmacological inhibition of PI3K/AKT effectively neutralized the oncogenic effects attributed to FBXO11, highlighting the pathway's centrality [64]. Future studies may consider understanding how PI3K/AKT and EMT are connected to macrophage polarization. In fact, future studies could demonstrate that PI3K/AKT‐induced EMT in macrophages enhances M2 polarization in carcinogenesis or has no impact. A critical molecular mechanism underlying cancer metastasis has been identified, in which an alternatively spliced variant of a cytokine significantly promotes EMT, enhances cellular invasion, and accelerates metastatic progression in breast cancer. Marked downregulation of the epithelial marker E‐cadherin and upregulation of the mesenchymal marker N‐cadherin were observed, alongside increased Akt phosphorylation, indicating activation of the PI3K/Akt signaling pathway. These alterations resulted in greater invasive capacity of tumor cells in vitro and increased metastatic burden in vivo, ultimately leading to reduced survival. Importantly, these effects were reversed upon inhibition of the PI3K/Akt pathway, suggesting a direct mechanistic link and highlighting a potential therapeutic target for intervention [65]. The studies highlighted the pivotal role of the PI3K/AKT pathway in driving EMT and metastatic progression across multiple cancers, including ovarian, gastric, and breast cancers. Key regulators, such as PAK5, FBXO11 and an alternatively spliced cytokine variant, converge on PI3K/AKT activation to promote EMT, downregulate epithelial markers (E‐cadherin), and upregulate mesenchymal markers (N‐cadherin, vimentin), thereby enhancing cell migration, invasion, and metastasis. Notably, pharmacological inhibition of PI3K/AKT reverses these effects, suggesting its therapeutic potential. Future research should explore the broader implications of PI3K/AKT‐mediated EMT in the TME, particularly its influence on immune cell polarization, such as macrophages, in breast cancer. Investigating whether PI3K/AKT‐induced EMT drives M2 macrophage polarization, a protumorigenic phenotype could reveal novel mechanisms of immune evasion and metastasis, offering new targets for combination therapies aimed at disrupting both EMT and immunosuppressive microenvironments.

EMT modulation can occur via PI3K/AKT through interactions with different pathways, such as transforming growth factor beta (TGF‐β), nuclear factor kappa‐light‐chain‐enhancer of activated B cells (NF‐κB), tyrosine kinase receptors, and Wnt/β‐catenin. TGF‐β is a cytokine capable of inducing EMT, which can occur as a result of PI3K/AKT upregulation, either directly through RhoA or indirectly via EGFR regulation. Such events can mediate the phosphorylation of transcription factors, such as Twist, and induce TGF‐β2 production, providing a positive feedback loop that facilitates EMT. Additionally, PI3K/AKT plays a significant role in EMT induction through NF‐κB, as highlighted in breast cancer, where hyperactivation of NF‐κB and Snail expression can downregulate E‐cadherin and enhance invasiveness, independent of TGF‐β. Ras, a tyrosine kinase receptor, can control PI3K/AKT and ERK to accelerate EMT and tumorigenesis. Its absence leads to the suppression of PDGF‐mediated AKT activation and MMP2 release, decreasing migration and invasion. The Wnt/β‐catenin pathway affects PI3K/AKT, elevating the nuclear translocation and transcriptional function of β‐catenin through its phosphorylation at Ser552, while simultaneously downregulating GSK3β via phosphorylation at Ser9. This event suppresses the degradation of both β‐catenin and Snail, thereby increasing EMT [66].

The studies have highlighted the role of PI3K/AKT in EMT modulation, tumorigenesis, and therapy resistance. Transforming acidic coiled‐coil‐containing protein 3 (TACC3) is recognized as a vital oncogenic driver that accelerates EMT through the activation of PI3K/AKT and ERK. Its upregulation enhances proliferation, metastasis, and transformation, whereas its depletion overcomes the EMT phenotype, demonstrating its potential as a therapeutic target. Pleckstrin homology‐like domain family A member 2 (PHLDA2) is also upregulated in CRC, enhancing tumorigenesis and EMT via the PI3K/AKT pathway. Its silencing suppresses EMT and proliferation while stimulating autophagy and apoptosis through the PI3K/AKT/mTOR and PI3K/AKT/GSK‐3β pathways. Conversely, FAT atypical cahderin 4 (FAT4) can impair CRC by decreasing proliferation, migration, and EMT while enhancing autophagy; its functions are partially triggered through the downregulation of the PI3K/AKT pathway, highlighting the roles of both mTOR and GSK‐3β [67, 68, 69].

##### 2.3.1.2 Therapeutic Targeting of PI3K/AKT‐Induced EMT and Future Perspectives

Recent therapeutic advancements have demonstrated that targeting the PI3K/AKT/mTOR pathway can yield substantial anticancer effects, particularly by modulating EMT and enhancing drug sensitivity. In the context of GC, thymoquinone has shown promising antitumor effects with selective cytotoxicity against GC cells while sparing normal gastric epithelial cells. This treatment has achieved significant inhibition of cell proliferation, colony formation, and invasiveness in a dose‐dependent manner. Apoptotic pathways were activated, as indicated by the upregulation of proapoptotic markers and the downregulation of antiapoptotic proteins. EMT was effectively reversed, as evidenced by reduced expression of mesenchymal markers and increased levels of epithelial markers. Additionally, key components of the PI3K/AKT/mTOR pathway were downregulated, indicating that this pathway was disrupted [70]. Complementary findings have emerged in epithelial ovarian cancer (EOC), where chemoresistance has been a major clinical obstacle. Targeting the PI3K/AKT/mTOR axis has been shown to overcome this resistance while concurrently reversing EMT and reducing CSC characteristics. Specifically, enhanced chemoresistance and metastatic potential in EOC have been correlated with increased EMT and CSC traits, all of which are associated with elevated PI3K/AKT/mTOR activity. Upon inhibition of this pathway using BEZ235 in combination with cisplatin (DDP), significant decreases were observed in clonogenicity, EMT markers (N‐cadherin, vimentin), and CSC markers (CD44v6, CD117, ALDH1A1, Snail), while apoptosis and ROS levels were significantly elevated. These outcomes suggest that cotargeting the PI3K/mTOR pathway with standard chemotherapy may effectively restore drug sensitivity and mitigate malignant progression [71]. Together, these findings support the therapeutic value of cotargeting the PI3K/mTOR pathway alongside conventional chemotherapy. This approach not only reduces tumor aggressiveness but also restores chemosensitivity and impedes metastatic progression.

This section highlights the essential role of the PI3K/AKT pathway in modulating EMT, which contributes to tumor cell dissemination and drug resistance. The PI3K/AKT pathway interacts with numerous upstream regulators, including TGF‐β, NF‐κB, tyrosine kinase receptors, and Wnt/β‐catenin, to facilitate EMT. This process is driven by the transcriptional activation of factors such as Snail, Slug, and Twist, leading to increased invasion, proliferation, and drug resistance. Various oncogenic drivers (APOC2, POLR3G, CPA4, GPX2) and suppressors (FAT4) influence carcinogenesis by regulating the PI3K/AKT pathway, further emphasizing its significance. However, a fundamental limitation of current studies is the lack of mechanistic insights into how PI3K/AKT–EMT interactions interface with immunological regulation, such as macrophage polarization, and the TME. Future research should focus on elucidating these relationships and exploring combinatorial treatment strategies targeting the PI3K/AKT pathway and EMT to address metastasis and drug resistance.

#### 2.3.2 PI3K/AKT/mTOR and Autophagy

##### 2.3.2.1 Role of PI3K/AKT/mTOR in Regulating Autophagy and Tumor Adaptation

Autophagy is a primary process that dynamically regulates cellular homeostasis through the degradation and recycling of damaged organelles, misfolded proteins, and unwanted cellular components. As a lysosome‐dependent mechanism, autophagy involves lysosomes in the degradation of cellular materials. It can affect survival by providing nutrients and energy under conditions such as hypoxia and nutrient deprivation. Additionally, autophagy contributes to the quality control by reducing the accumulation of toxic substances. Dysregulation of autophagy has been associated with the pathogenesis of various diseases, including cancer [72, 73], neurodegenerative diseases [74, 75], cardiovascular diseases [76, 77], metabolic disorders [78, 79], and infections [80]. Therefore, understanding the molecular pathways regulating autophagy is crucial. This section focuses on the interaction between the PI3K/AKT/mTOR axis and autophagy in human cancers.

The TME is a multifaceted and dynamic system that includes mesenchymal stem cells, fibroblasts, immune cells, endothelial precursors, blood vessels, and secreted cytokines. These components affect carcinogenesis. The TME is frequently characterized by hypoxia, acidosis, inflammation, and nutritional deficiency, all of which are factors that accelerate autophagy. This process serves as a survival mechanism, improving the adaptation of tumor cells to these conditions. The PI3K/AKT/mTOR axis is considered vital for controlling autophagy in the TME. It affects activities such as angiogenesis through HIF‐1α translation under hypoxic conditions and the survival of endothelial cells. Environmental stresses, such as hypoxia and acidic pH, can stimulate autophagy by suppressing AKT phosphorylation. Compounds such as quinacrine and cediranib facilitate autophagy under hypoxic conditions, thereby demonstrating antiangiogenic and anticancer properties through PI3K/AKT downregulation. Similarly, the natural substance DT‐13 demonstrates antiangiogenic activity by enhancing autophagy via a comparable mechanism. Acidic conditions can induce autophagy‐related morphological changes, cell cycle arrest, and decreased proliferation. They can also affect immunological reactions, including M2 macrophage polarization in breast tumors, via the mTOR and ROS/ERK pathways. Telomerase components, such as TERT, modulate mTORC1 to stimulate autophagy, which is essential for nutrient recycling in metabolically stressed neoplastic cells. In pancreatic tumors, drugs including ancistrolikokine E can impair cellular adaptation to the nutritional deprivation by targeting the AKT/mTOR/autophagy pathway. Furthermore, the inflammatory receptor RAGE enhances autophagy and suppresses apoptosis, thereby promoting pancreatic tumor survival [81].

FGF21 expression is significantly downregulated in prostate cancer (PCa) tissues and cell lines. FGF21 inhibits the proliferation, clone formation, migration, and invasiveness of LNCaP cells and promotes apoptosis. Additionally, it attenuates high glucose‐induced proliferation and apoptosis in LNCaP cells. Mechanistically, FGF21 promotes autophagy in LNCaP cells by inhibiting the PI3K/AKT/mTOR pathway. In vivo experiments have shown that FGF21 overexpression inhibits PCa tumorigenesis in nude mice. These findings suggest that FGF21 is a potential novel target for PCa therapy [82].

**FIGURE 4:**
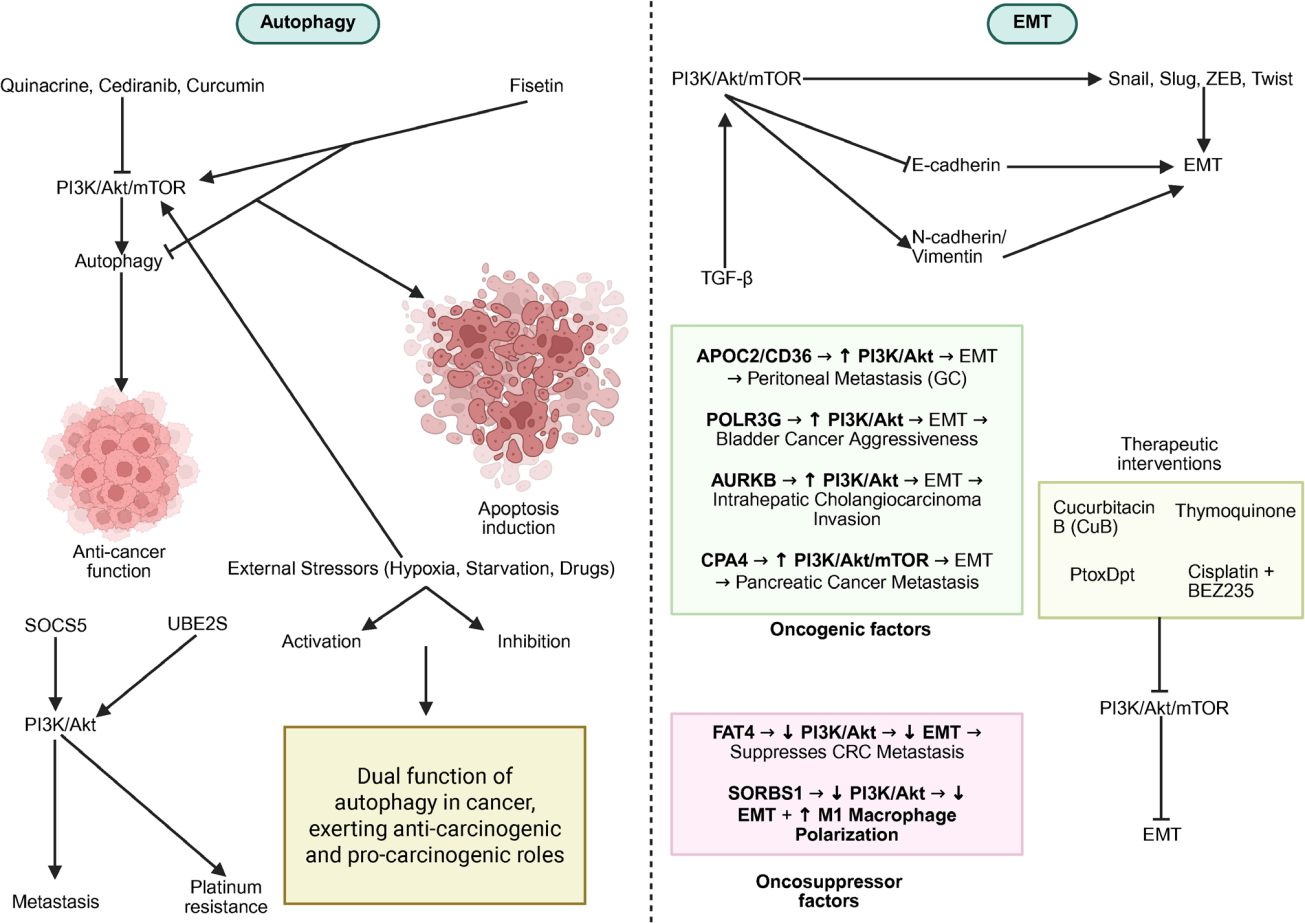
The function of PI3K/AKT in the modulation of autophagy and EMT. Several kinds of therapeutic compounds have been introduced to regulate PI3K/AKT/mTOR axis in affecting EMT and autophagy in human cancers including quinacrine, ceiranib, curcumin, cucurbitacin B, thymoquinone, PtoxDpt, and cisplatin. The problem is related to the dual function of autophagy in cancer providing some challenges regarding induction or inhibition of PI3K/AKT/mTOR axis to modulate it. PI3K/AKT/mTOR axis has been able to regulate EMT in the different cancers including gastric cancer, bladder tumor, and pancreatic cancer, among others; (created with Biorender.com).

Autophagy has emerged as a key cellular process implicated in cancer drug resistance, often regulated by the PI3K/AKT/mTOR axis. Several recent studies have elucidated the molecular mechanisms by which dysregulation of this pathway contributes to the chemoresistance through modulation of autophagic activity. In OC, the ubiquitin‐conjugating enzyme UBE2S has been identified as a major player in promoting DDP resistance by inhibiting autophagy through activation of the PI3K/AKT/mTOR pathway. A significant correlation has been demonstrated between elevated UBE2S expression and chemoresistance. Activation of the PI3K/AKT/mTOR pathway by UBE2S leads to the inhibition of autophagy, thereby promoting DDP resistance in OC. Significantly higher UBE2S levels were observed in resistant samples compared with sensitive ones. Knockdown of UBE2S impaired cell proliferation, migration, and tumor growth both in vitro and in vivo. Enhanced autophagic activity was induced upon UBE2S silencing, as indicated by increased autophagy‐related proteins, and this effect was reversed by an mTOR agonist, confirming the regulatory axis. These results highlight the critical role of UBE2S in modulating autophagy‐mediated drug resistance via the PI3K/AKT/mTOR signaling cascade [83]. Similarly, the transcription factor E2F2 plays a dual role in GC, contributing to tumor aggressiveness and immune modulation through autophagy suppression. A strong association has been identified between elevated E2F2 expression and the aggressive biological behavior of GC cells. Poor OS has been linked to high E2F2 levels, which were significantly upregulated in GC tissues and cell lines. Cellular migration and invasiveness were promoted, while autophagy was inhibited through activation of the PI3K/AKT/mTOR pathway. In contrast, E2F2 knockdown led to enhanced autophagic activity, reduced motility, and invasion of GC cells. Furthermore, alterations in E2F2 expression influenced immune infiltration, particularly in relation to PD‐1/PD‐L1 and various T‐cell subsets. These findings suggest a dual role for E2F2 in modulating both tumor progression and immune response through autophagy regulation [84]. Autophagy has also been linked to the resistance to oxaliplatin (OXA) in GC, mediated by elevated expression of ANXA1. Interestingly, unlike UBE2S and E2F2, ANXA1 promotes autophagy by suppressing the PI3K/AKT/mTOR pathway. A strong correlation has been demonstrated between ANXA1 upregulation and increased OXA resistance in GC. This resistance is driven by autophagy, which is promoted through suppression of the PI3K/AKT/mTOR pathway. It has been observed that ANXA1 expression is significantly elevated in OXA‐resistant GC cells and tissues. High levels of ANXA1 are associated with reduced apoptosis, enhanced autophagic flux, and poor OS. Moreover, inhibition of ANXA1 resensitizes resistant cells to OXA both in vitro and in vivo, without affecting baseline proliferation. This indicates that chemoresistance, rather than proliferation, is primarily regulated by ANXA1 [85]. In cervical cancer (CC), autophagy regulation occurs through a different axis involving miR‐338 and its downstream target ATF2, again implicating the PI3K/AKT/mTOR pathway in controlling both autophagic flux and cell proliferation. A significant inhibitory effect on cell proliferation and autophagy was observed through modulation of ATF2 by miR‐338. Reduced levels of miR‐338 were associated with increased expression of ATF2, enhanced autophagy, and higher proliferative capacity in CC cells. Upon restoration of miR‐338, suppression of both autophagy‐related markers (such as LC3‐II) and proliferative behaviors was achieved. The autophagic activity promoted by miR‐338 inhibition mirrored the effect of rapamycin, indicating involvement of mTOR regulation. Mechanistically, miR‐338 was shown to target ATF2 and regulate the PI3K/AKT/mTOR pathway, resulting in downstream changes in p‐mTOR and p‐p70S6 levels. These findings suggest that autophagy suppression is mediated through this signaling cascade [86]. Therefore, increasing evidence demonstrates that autophagy and the PI3K/AKT/mTOR axis interact in the regulation of tumorigenesis. Although the role of the PI3K/AKT pathway in accelerating tumorigenesis has been confirmed, in the case of protective autophagy, activation of the PI3K/AKT/mTOR axis can suppress autophagy to exert anticancer activity. This highlights the dual function of both autophagy and the PI3K/AKT/mTOR axis. Table 2 summarizes the role of PI3K/AKT axis in the regulation of EMT in human cancers. Figure 4 highlights the role of PI3K/AKT in the regulation of autophagy and EMT.

**TABLE 2:** The role of PI3K/AKT axis in EMT regulation.

Cancer	Outcome	References
Gastric cancer	Upregulation of TREM2 stimulates EMT through PI3K/AKT upregulation	[87]
Gastric cancer	GRSF1 stimulates PI3K/AKT to accelerate EMT‐driven metastasis	[88]
Colorectal cancer	Echinacoside impairs tumorigenesis through inhibition of PI3K/AKT downregulation	[89]
Non‐small cell lung cancer	CCL2 stimulates PI3K/AKT and autophagy to increase invasion and EMT	[90]
Bladder cancer	POLR3G stimulates EMT through PI3K/AKT upregulation	[57]
Cholangiocarcinoma	EMT induction by PLCB1/PI3K/AKT axis in enhancing tumorigenesis	[91]
Prostate cancer	Quercetin suppresses PI3K/AKT axis and enhances apoptosis	[92]
Esophageal cancer	NETO2 upregulates ERK, PI3K/AKT, and Nrf2 to enhance growth and invasion	[93]
Gastric cancer	Apolipoprotein C‐II mediates EMT in enhancing PI3K/AKT axis to promote tumorigenesis and peritoneal metastasis	[56]
Bladder cancer	TEAD4 stimulates PI3K/AKT to promote EMT	[94]
Pancreatic cancer	Paracrine HGF stimulates EMT and enhances drug resistance through c‐Met/PI3K/AKT upregulation	[95]
Colorectal cancer	MYSM1 upregulates miR‐200/CDH1 and downregulates PI3K/AKT to disrupt cancer progression	[96]
Breast cancer	TSP50 induces PI3K p110α‐driven AKT axis to enhance EMT and stem cell features	[97]
Cervical cancer	TIM‐1 upregulates PI3K/AKT axis to increase growth and invasion	[98]
Cervical cancer	Magnolol and 5‐fluorouracil exerts synergistic impact in the suppression of metastasis through downregulation of Snail, Slug and vimentin, and increasing E‐cadherin levels	[99]
Lung cancer	Cancer/testis antigen LDHC upregulates PI3K/AKT/GSK‐3β axis in enhancing metastasis and growth	[100]
Breast cancer	*Hypericum roeperianum* bark extract reduces PI3K/AKT expression and suppresses EMT	[101]
Pancreatic cancer	FAM126A demonstrates interaction with ENO1 to stimulate PI3K/AKT axis to promote growth and invasion	[102]
Liver cancer	NCAPD2 upregulates PI3K/AKT/mTOR axis to promote carcinogenesis	[103]

Abbreviations: β‐catenin, beta‐catenin; AKT, protein kinase B; CCL2, C‐C motif chemokine ligand 2; CD24, cluster of differentiation 24; CDH1, cadherin 1; circPIP5K1A, circular RNA phosphatidylinositol‐4‐phosphate 5‐kinase type‐1 alpha; E‐cadherin, epithelial cadherin; EMT, epithelial–mesenchymal transition; ENO1, enolase 1; ERK, extracellular signal‐regulated kinase; FAM126A, family with sequence similarity 126 member A; FGF2, fibroblast growth factor 2; FGFR1, fibroblast growth factor receptor 1; GRSF1, G‐rich RNA sequence binding factor 1; GSK‐3β, glycogen synthase kinase 3 beta; HGF, hepatocyte growth factor; IGFBP7, insulin‐like growth factor binding protein 7; KLF2, Kruppel‐like factor 2; KRT80, keratin 80; LDHC, lactate dehydrogenase C; miR‐200, microRNA‐200; miR‐671‐5p, microRNA‐671‐5p; mTOR, mechanistic target of rapamycin; MYSM1, Myb‐Like, SWIRM, and MPN domains 1; NCAPD2, non‐SMC condensin I complex subunit D2; Nrf2, nuclear factor erythroid 2‐related factor 2; p110α, PI3K catalytic subunit alpha; PI3K, phosphoinositide 3‐kinase; PLCB1, phospholipase C beta 1; POLR3G, RNA polymerase III subunit G; SLC4A7, solute carrier family 4 member 7; Slug, zinc finger protein SNAI2; Snail, zinc finger protein SNAI1; TEAD4, TEA domain transcription factor 4; TIM‐1, T‐cell immunoglobulin and mucin‐domain containing‐1; TIMP‐4, tissue inhibitor of metalloproteinases 4; TREM2, triggering receptor expressed on myeloid cells 2; TSP50, testes‐specific protease 50; WNT, wingless‐type MMTV integration site family.

##### 2.3.2.2 Therapeutic Targeting of PI3K/AKT‐Driven Autophagy in Cancer

The interplay between the PI3K/AKT/mTOR pathway and autophagy forms a vital axis in cancer growth and therapeutic response, as highlighted in studies across various tumor types. Autophagy is essential for cellular homeostasis and is tightly regulated by the PI3K/AKT/mTOR pathway, which functions as a negative regulator in nutrient‐rich and growth‐stimulating environments. In CRC, lomerizine 2HCl, a drug initially used for migraine alleviation, has been shown to suppress the PI3K/AKT/mTOR pathway, leading to decreased protective autophagy and reduced apoptosis. However, when combined with an autophagy inhibitor such as 3‐methyladenine (3‐MA), this treatment accelerates apoptosis. This demonstrates that autophagy inhibition can enhance the therapeutic response of CRC cells. In CC, a new polysaccharide from *Rosa rugosa* (RRP) has been shown to stimulate autophagy and increase apoptosis in a dose‐dependent manner via the ROS‐mediated mitochondrial pathway. The PI3K/AKT/mTOR axis serves as a key regulatory hub in this process. Autophagy can precede apoptosis, highlighting a sequence of events where autophagy promotes apoptosis. In PCa, FGF21 has been shown to function as a tumor suppressor by accelerating autophagy while reducing proliferation and metastasis. FGF21 suppresses the PI3K/AKT/mTOR/70S6K pathway, demonstrating that downregulation of this axis stimulates autophagy to restrict tumor growth and enhance apoptosis. Similarly, in oral cancer, chlorpromazine (CPZ) exerts anticancer effects by causing cell cycle arrest and cell death while also accelerating autophagy via modulation of the PI3K/AKT/mTOR/p70S6K pathway. CPZ‐mediated cell death is reduced by downregulation of both caspases and autophagy, highlighting the synergistic relationship between autophagy and apoptosis [104, 105, 106, 107].

Increasing evidence has highlighted the role of the PI3K/AKT/mTOR pathway in modulating autophagy in cancers. This pathway is a critical regulator of carcinogenesis and therapeutic resistance. Fisetin has been shown to suppress autophagy in hepatocellular carcinoma by activating PI3K/AKT/mTOR and inhibiting AMPK, resulting in autophagy inhibition and increased apoptosis. Plumbagin also suppressed PI3K/AKT and triggered autophagy and cell death in endometrial carcinoma cells. However, its anticancer effects were diminished by autophagy suppression, demonstrating the significance of autophagy in regulating therapeutic efficacy. Sodium cantharidate stimulated autophagy and apoptosis in breast tumors by blocking the PI3K/AKT/mTOR pathway. Upregulation of mTOR was found to overcome these effects, thereby highlighting the essential role of this axis in autophagy‐mediated tumor suppression. In esophageal squamous cell carcinoma, curcumin enhanced the antitumor efficacy of docetaxel by triggering apoptosis and autophagy through downregulation of the PI3K/AKT/mTOR pathway. Additionally, downregulation of autophagy further sensitized cells to therapy, demonstrating a therapeutic advantage in modulating autophagy. Sirtuin 3 downregulated PI3K/AKT/mTOR in lung tumors, thereby restoring autophagy flux and reducing doxorubicin‐mediated senescence and oxidative damage. Induction of autophagy emerged as a crucial factor in these processes [108, 109, 110, 111].

A study investigated the anticancer effects of apigenin, a dietary flavonoid, on HCC cells both in vitro and in vivo. The results showed that apigenin inhibits cell growth and induces apoptosis in HepG2 cells in a dose‐ and time‐dependent manner. It increases the expression of LC3‐II and the number of GFP–LC3 puncta, indicating autophagy induction. Inhibition of autophagy with 3‐MA or Atg5 gene silencing enhances apigenin‐induced proliferation inhibition and apoptosis, suggesting that apigenin‐induced autophagy has a protective effect against cell death. Mechanistically, apigenin induces apoptosis and autophagy through inhibition of the PI3K/AKT/mTOR pathway. In vivo experiments using a xenograft mouse model demonstrated that apigenin administration decreases tumor growth, and autophagy inhibition by 3‐MA significantly enhances the anticancer effect of apigenin. The study concludes that combining autophagy inhibitors with apigenin could be a potential chemotherapeutic strategy against hepatocellular carcinoma [112]. Although these studies do not demonstrate the direct interaction between the PI3K/AKT/mTOR pathway and autophagy, they provide valuable insights into the dual function of autophagy. Notably, autophagy is a regulator of other cell death mechanisms, including apoptosis. This has also been observed in the case of ferroptosis, where autophagy acts as a modulator. Therefore, regulating the PI3K/AKT/mTOR pathway to induce toxic autophagy can accelerate apoptosis and ferroptosis in cancers. Inhibition of protective autophagy can increase cell death. Since mTOR is a negative regulator of autophagy, its upregulation can suppress autophagy, which is beneficial in enhancing cell death. However, this scenario is valid only when autophagy has a protective function.

Salidroside, a natural compound extracted from *Rhodiola rosea*, was found to inhibit the growth of human GC AGS cells both in vitro and in vivo. It induced apoptosis in AGS cells, as evidenced by increased apoptosis rates, chromatin condensation, downregulation of antiapoptotic genes, upregulation of proapoptotic genes, and activation of caspase proteins. Salidroside also induced autophagy, as shown by the presence of autophagosomes, increased LC3‐II levels, and elevated expression of autophagy‐related proteins. The compound inhibited the activation of the PI3K/AKT/mTOR pathway, which was involved in both apoptosis and autophagy induction. Inhibition of autophagy with chloroquine enhanced salidroside‐induced apoptosis, indicating that salidroside‐mediated autophagy protected AGS cells from death. In an in vivo tumor xenograft trial, salidroside significantly reduced tumor volume and weight in nude mice without causing noticeable adverse effects on normal tissues [113]. Building on these results, additional analyses have deepened understanding of the interplay between autophagy and apoptosis, particularly in the context of therapeutic resistance and survival pathways. Although the regulation of the PI3K/AKT/mTOR axis in the treatment of cancer by natural and small molecules will be discussed in the next sections, the current findings support the notion that PI3K/AKT/mTOR‐driven autophagy can be therapeutically modulated. Moreover, these studies highlight an important fact: inhibition of the PI3K/AKT/mTOR axis by anticancer compounds and subsequent autophagy induction do not necessarily provide an anticancer function for autophagy. Instead, protective autophagy may be induced, and its inhibition can increase cell death in the form of apoptosis in tumor cells. A significant limitation of current studies is that, when evaluating the anticancer activity through autophagy inhibition, the primary focus has been on apoptosis. However, autophagy can also affect ferroptosis and other cell death mechanisms, which should be comprehensively evaluated.

ABBV‐744 was found to inhibit the growth of GC cells and patient‐derived tumor organoids in a dose‐dependent manner. It induced mitochondrial damage, ROS accumulation, cell cycle arrest, and apoptotic cell death. Transcriptomic analysis identified autophagy as a crucial pathway involved in ABBV‐744‐induced cell death. Mechanistically, ABBV‐744 inactivated the PI3K/Akt/mTOR/p70S6K pathway and activated the MAPK to induce autophagy flux. In vivo studies demonstrated that ABBV‐744 significantly suppressed GC cell growth via autophagy induction [114].

The current section highlights the dual function of autophagy in cancer, adding further complexity to its role through the PI3K/AKT/mTOR pathway. Autophagy is responsible for preserving cellular homeostasis by recycling damaged components and can improve survival under conditions such as hypoxia and starvation, which are common in the TME. The PI3K/AKT/mTOR pathway has been identified as a key regulator of autophagy, suppressing it in nutrient‐rich conditions. However, inhibition of the PI3K/AKT pathway can mediate autophagy. Studies across different cancers have demonstrated that modulation of the PI3K/AKT/mTOR axis can affect both survival and toxic autophagy. Therefore, both upregulation and downregulation of the PI3K/AKT/mTOR pathway can be observed in the context of autophagy regulation in cancer therapy. Natural substances (apigenin, baicalein, thymoquinone) and small molecules (ABBV‐744, fisetin) have exerted their antitumor functions via regulation of this axis. However, the role of autophagy differs between protective and cytotoxic, depending on the TME and the type of stimuli. This has been considered a significant challenge in the regulation of autophagy function. Despite its role in cancer suppression in some cases, autophagy has also been beneficial for enhancing resistance and survival in other tumors.

#### 2.3.3 PI3K/AKT/mTOR and Apoptosis

##### 2.3.3.1 PI3K/AKT/mTOR Regulation of Apoptosis in Cancer

Apoptosis is a distinct form of cell death that is vital for preserving cellular homeostasis and development through the elimination of damaged, dysfunctional, or aged cells. Apoptosis can be identified by unique morphological changes, including cell shrinkage, chromatin condensation, membrane blebbing, and the formation of apoptotic bodies, which are engulfed by phagocytes. Dysregulation of apoptosis has been demonstrated in numerous pathological conditions, including cancer, where a lack of apoptosis enhances the proliferation of cancer cells. Recent studies have highlighted that the PI3K/AKT pathway is a critical regulator of apoptosis in human cancers. KIF15 was found to be highly expressed in PCa tissues, correlating with tumor invasion depth and poor prognosis. Knockdown of KIF15 in PCa cells significantly inhibited cell proliferation, colony formation, and migration, while promoting apoptosis. Conversely, KIF15 overexpression had the opposite effects. Mechanistically, KIF15 knockdown upregulated proteins such as CD40L, cytochrome *c* (Cyt *c*), DR6, and p21, while downregulating IGF‐I and Survivin. This process was shown to involve the PI3K/Akt pathway. In vivo experiments further confirmed that KIF15 knockdown inhibited tumor growth in a mouse xenograft model, with downregulation of KIF15, p‐PI3K, and p‐Akt in the tumors [115]. Various anticancer compounds have demonstrated potential in regulating apoptosis through their effects on the PI3K/AKT pathway [116, 117, 118]. Notably, the regulation of the PI3K/AKT/mTOR axis can affect both apoptosis and autophagy in cancers. Several anticancer compounds, including salidroside [113], piperlongumine [119], thymoquinone [120], and eugenol [121], have been shown to modulate the PI3K/AKT/mTOR axis, thereby influencing both autophagy and apoptosis. Despite the regulation of the PI3K/AKT/mTOR axis and its impact on apoptosis and autophagy, the final role of autophagy in human cancers remains uncertain. In fact, autophagy can exert both protective and toxic functions. Moreover, autophagy can influence apoptosis. Although inhibition of the PI3K/AKT/mTOR axis can reduce tumorigenesis and apoptosis, it can also mediate protective autophagy, thereby enhancing cancer progression. This highlights the dual function of PI3K/AKT regulation in cancer therapy.

##### 2.3.3.2 Therapeutic Targeting of PI3K/AKT/mTOR to Modulate Apoptosis

Accordingly, the evasion of apoptosis has been highlighted as a hallmark of cancer. Apoptosis can be regulated by the PI3K/AKT/mTOR axis in human cancers, and its dysregulation further enhances tumorigenesis. Regulation of the PI3K/AKT/mTOR axis or its upstream mediators, such as PTEN, can enhance apoptosis and suppress carcinogenesis. Various anticancer compounds, including cryptotanshinone, salidroside, piperlongumine, thymoquinone, and eugenol, have been used to regulate the PI3K/AKT/mTOR axis, thereby controlling apoptosis and autophagy. The interaction among these pathways increases therapeutic complexity, since suppression of the PI3K/AKT axis can concurrently trigger protective autophagy, thereby decreasing therapy effectiveness. This highlights the dual function of the PI3K/AKT/mTOR axis: its downregulation can trigger apoptosis and diminish tumorigenesis. A significant limitation of existing research is the variable results of autophagy induction among different cancer types. Therefore, a mechanistic understanding of the regulation of apoptosis and autophagy by the PI3K/AKT/mTOR axis can accelerate the development of cancer treatments.

#### 2.3.4 PI3K/AKT/mTOR and Ferroptosis

##### 2.3.4.1 Mechanistic Role of PI3K/AKT/mTOR in Ferroptosis Regulation

Ferroptosis is another mechanism of cell death, caused by iron‐dependent lipid peroxidation and oxidative damage to the cell membrane. Ferroptosis is different from apoptosis and necrosis, and it is driven by metabolic dysfunctions involving iron and ROS generation. The induction of ferroptosis can decrease carcinogenesis. Drug resistance can occur as a result of ferroptosis inhibition. For more information on the role of ferroptosis in cancer, see these reviews [122, 123, 124, 125]. A significant tumor‐suppressive role has been demonstrated for ferroptosis, characterized by the inhibition of CRC cell invasion, migration, and EMT. Significantly reduced expression of KLF2 has been observed in CRC tissues, correlating with poor prognosis. Overexpression of KLF2 has been shown to suppress tumor growth both in vitro and in vivo. Mechanistically, ferroptosis is induced through upregulation of GPX4, while the PI3K/AKT is effectively inhibited. EMT markers such as E‐cadherin have been increased, whereas N‐cadherin and vimentin have been decreased, reinforcing the antimetastatic impact. These functional effects have been confirmed through both molecular and xenograft models, supporting KLF2 as a promising therapeutic target in CRC [126]. These results underscore the dual role of KLF2 in regulating both tumor suppression and ferroptosis, offering a mechanistic basis for targeting the PI3K/AKT in CRC therapy. The role of TIMP1 in sorafenib‐induced ferroptosis in CRC was investigated using HCT‐8 cell lines with altered TIMP1 expression. TIMP1 overexpression was found to induce resistance to sorafenib, while its knockdown suppressed the PI3K/AKT pathway and reduced GPX4 levels, thereby enhancing ferroptosis. Increased levels of ROS, iron, and malondialdehyde (MDA) were observed following TIMP1 depletion. In sorafenib‐resistant cells, cotreatment with sorafenib and the GPX4 inhibitor RSL3 triggered ferroptosis, leading to elevated iron and lipid peroxide levels and reduced cell viability. These findings suggest that TIMP1 inhibition and GPX4 targeting may enhance sorafenib efficacy in CRC [127]. Collectively, these findings highlight the importance of TIMP1 as a modulator of ferroptotic sensitivity and suggest potential combination strategies to overcome drug resistance in CRC. Cancer cells with mutations in PI3K or lacking PTEN are not vulnerable to ferroptosis; however, blocking the PI3K/AKT/mTOR pathway can render them sensitive. Resistance stimulates mTORC1 and induces SREBP1, modulating lipid metabolism. SCD1, regulated by SREBP1, contributes to the inhibition of ferroptosis through fatty acid synthesis. Inhibiting SREBP1 or SCD1 makes cancer cells with PI3K/AKT/mTOR mutations more susceptible to ferroptosis [128]. This provides a rationale for targeting lipid metabolic regulators such as SREBP1 and SCD1 in PI3K/AKT/mTOR‐mutant cancers, thereby overcoming intrinsic resistance to ferroptosis. SLC7A11 was significantly upregulated in GC tissues compared with surrounding tissues. Inhibiting SLC7A11 diminished cell growth, migration, and invasion and enhanced susceptibility to ferroptosis by controlling levels of ROS and lipid peroxidation. Upregulating SLC7A11 partially alleviated ferroptosis caused by erastin. Blocking SLC7A11 led to deactivation of the PI3K/AKT pathway, elevating lipid peroxidation and ultimately inhibiting the progression of GC [129]. These insights establish SLC7A11 as a critical node connecting ferroptosis regulation and PI3K/AKT pathway activity, suggesting its potential as a therapeutic target in gastric cancer.

##### 2.3.4.2 Therapeutic Modulation of PI3K/AKT‐Driven Ferroptosis in Cancer

Curcumin inhibits CRC cell proliferation by inducing ferroptosis, as demonstrated in HCT‐8 cells. Blocking ferroptosis with Fer‐1 reduced curcumin's antiproliferative effect, suggesting a ferroptosis‐dependent mechanism. Curcumin decreased levels of GSH, SLC7A11, and GPX4, while increasing levels of iron, MDA, and ROS, which is consistent with ferroptosis induction. Curcumin also suppressed the PI3K/AKT/mTOR pathway, as validated using a PI3K agonist (which reversed the effects) and a PI3K inhibitor (which enhanced them). Overall, curcumin promotes ferroptosis and inhibits CRC cell growth via suppression of the PI3K/AKT/mTOR pathway, highlighting its potential as a therapeutic agent for CRC [130].

**FIGURE 5:**
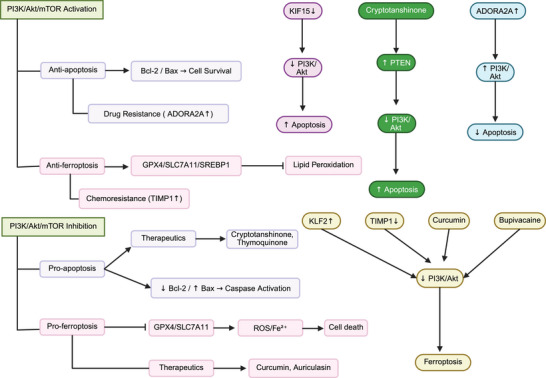
The regulation of apoptosis and ferroptosis by PI3K/AKT/mTOR. Upregulation of this pathways reduces apoptosis and ferroptosis causing chemoresitance, while its inhibition enhances cell death. A number of therapeutics including curcumin, auriculasin, bupivacaine, and cryptotanshinone can downregulate PI3K/AKT to stimulate apoptosis and ferroptosis; (created with Biorender.com).

The importance of ferroptosis, an iron‐dependent form of cell death distinct from apoptosis and necrosis was evaluated in the current section, demonstrating its potential in cancer therapy. Lipid peroxidation and oxidative damage can stimulate ferroptosis, which is important for overcoming tumor proliferation and chemoresistance. Dysregulation or inhibition of ferroptosis can enhance carcinogenesis. The PI3K/AKT/mTOR pathway has been identified as a regulator of ferroptosis, and its downregulation increases ferroptosis in cancers. According to studies, downregulation of the PI3K/AKT/mTOR pathway can enhance ferroptosis induction in cancer cells, especially those with PI3K mutations or PTEN deficiency, by downregulating downstream factors such as GPX4, SREBP1, and SLC7A11. These factors are crucial regulators of antioxidant defense and lipid metabolism. Natural chemicals and targeted therapeutics, including curcumin, auriculasin, bupivacaine, and nitidine chloride, accelerate ferroptosis via downregulation of the PI3K/AKT/mTOR, highlighting the therapeutic significance of this axis. A drawback of the current results is the context‐dependent susceptibility to ferroptosis, as several oncogenic alterations in the PI3K pathway mediate intrinsic resistance. This duality in function highlights the essential role of integrating PI3K/AKT/mTOR inhibitors with ferroptosis‐inducing drugs. Moreover, an interesting area for future development is the development of nanoparticles for targeting the PI3K/AKT/mTOR pathway to accelerate ferroptosis in a targeted manner. Although previous studies have developed nanostructures for stimulating ferroptosis in cancer [131, 132], their impact on the PI3K/AKT/mTOR pathway requires better understanding. Figure 5 highlights the potential of the PI3K/AKT pathway in the modulation of apoptosis and ferroptosis.

#### 2.3.5 PI3K/AKT/mTOR and Glycolysis

##### 2.3.5.1 Mechanistic Role of PI3K/AKT/mTOR in Cancer Glycolysis

Glycolysis is a unique type of energy metabolism in cancer cells. Even in the presence of oxygen, glycolysis can participate in ATP generation through glycolysis. This type of metabolism is also known as the Warburg effect. Therefore, a kind of respiratory impairment is observed in tumor cells [133]. This foundational understanding highlights the centrality of glycolytic reprogramming in cancer metabolism and sets the stage for exploring its regulation by key oncogenic pathways. Although various molecular factors regulating the PI3K/AKT pathway have been identified, the PI3K/AKT axis is considered a major regulator of glycolysis in cancers. A strong association has been demonstrated between elevated PTPRH expression and enhanced tumor aggressiveness. Increased PTPRH levels have been linked to larger tumor size, advanced clinical stage, and poor prognosis in NSCLC. Glycolytic activity was significantly upregulated, as indicated by elevated 18F‐FDG uptake, lactate production, and overexpression of glycolysis‐related proteins such as GLUT1, HK2, PKM2, and LDHA. Furthermore, cell proliferation, migration, and invasion were enhanced, while apoptosis was suppressed. These effects were mediated via activation of the PI3K/AKT/mTOR, as verified by modulation with specific inhibitors and activators. Targeting PTPRH was shown to effectively reverse these malignant phenotypes [134]. These findings implicate PTPRH as a potent modulator of glycolysis and tumor aggressiveness via PI3K/AKT/mTOR activation, reinforcing its value as a therapeutic target in NSCLC. CPNE1 is pivotal in controlling cell growth and differentiation by triggering the AKT/mTOR pathway and is associated with various types of cancers, including breast cancer. Suppression of CPNE1 in triple‐negative breast cancer (TNBC) cell lines led to decreased cell viability, migration, invasion, and proliferation. Elevated CPNE1 levels increased aerobic glycolysis via the PI3K/AKT/HIF‐1α pathway, which was blocked by a PI3K inhibitor [135]. This highlights the critical role of the PI3K/AKT pathway in glycolysis in cancer. Together, these observations confirm the functional involvement of CPNE1 in driving aerobic glycolysis through the PI3K/AKT/HIF‐1α axis and its contribution to TNBC progression. Circ‐100395 expression was found to be significantly decreased in papillary thyroid cancer (PTC) tissues and cell lines compared with normal tissues and cells. Low levels of circ‐100395 were associated with advanced TNM stage, LN metastasis, and poor overall survival in PTC patients. Overexpression of circ‐100395 in PTC cells inhibited cell viability, colony formation, migration, and invasion, while promoting apoptosis. It also reduced aerobic glycolysis by decreasing glucose uptake and lactate production. Mechanistically, circ‐100395 downregulated the PI3K/AKT/mTOR, and these effects could be reversed by the PI3K activator 740Y‐P [136]. A strong association has been established between the overexpression of APLN and the promotion of oncogenic processes in CC. Elevated APLN levels have been linked to enhanced cell proliferation, migration, and glycolysis, while its silencing led to significant inhibition of these processes. These effects were shown to be mediated via the PI3K/AKT/mTOR, as evidenced by changes in pathway activation and downstream targets such as HK2, PFKP, and LDHA. Furthermore, pharmacological inhibition of this pathway or blockade of the APLNR receptor successfully reversed APLN‐induced tumorigenic behaviors. In vivo studies confirmed that APLN depletion suppressed tumor growth and increased apoptosis, indicating its critical role in cancer progression [137]. These findings highlight APLN as a critical upstream effector of PI3K/AKT/mTOR‐mediated glycolysis, with clear implications for targeted intervention in CC. An increased PLOD1 expression was associated with significant enhancement in cell proliferation and aerobic glycolysis, accompanied by suppression of apoptosis and tumorigenesis. Elevated levels of glucose uptake, ATP production, lactate output, and ECAR, alongside reduced OCR, were recorded under PLOD1 overexpression, indicating a metabolic shift toward the Warburg effect. Additionally, a positive regulatory relationship was identified between PLOD1 and the SOX9/PI3K/AKT/mTOR signaling axis, further supporting oncogenic activity. Inhibition of PLOD1 expression led to reduced tumor growth in vivo and downregulation of associated oncogenic markers, highlighting its potential as a therapeutic target in GC [138]. Collectively, these results establish PLOD1 as a driver of metabolic reprogramming in GC via the SOX9/PI3K/AKT/mTOR pathway, offering new avenues for metabolic therapy. A significant oncogenic role has been attributed to SPAG4 in CRC progression. Increased SPAG4 expression was associated with enhanced proliferation, migration, invasion, mitochondrial respiration, and aerobic glycolysis in CRC cells. Elevated mitochondrial function was evidenced by upregulation of key markers such as NDUFA1, SDHB, ATP5A, and PGC‐1α, while increased glycolytic activity was promoted through upregulation of GLUT1, HK2, LDHA, PKM2, and PFK1. These metabolic changes were mediated through activation of the PI3K/AKT/mTOR pathway and its downstream factor HIF‐1α. Inhibition of this pathway by LY294002 effectively neutralized the tumor‐promoting effects of SPAG4, highlighting its potential as a therapeutic target in CRC metabolic reprogramming [139]. The study further supports the pivotal role of SPAG4 in modulating both glycolysis and mitochondrial function, with the PI3K/AKT/mTOR‐HIF‐1α axis acting as the primary conduit of these oncogenic effects. An important mechanism underlying OSCC progression has been identified. The immunoregulatory protein B7‐H3 significantly enhances tumor aggressiveness by promoting aerobic glycolysis. Elevated expression of B7‐H3 has been observed in tumor tissues and is correlated with larger tumor size, LN metastasis, and recurrence. Increased glucose uptake and lactate production, indicative of a heightened Warburg effect, have been linked to B7‐H3 overexpression. This metabolic shift has been shown to be mediated by the PI3K/AKT/mTOR pathway, which upregulates HIF‐1α and its downstream glycolytic targets, GLUT1 and PFKFB3. These changes promote tumor cell proliferation, migration, invasion, and in vivo tumor growth. These findings underscore the role of B7‐H3 in tumor metabolism, independent of its immune function [140]. They also emphasize the metabolic dimension of B7‐H3's oncogenic function, linking its expression to enhanced glycolysis through PI3K/AKT/mTOR in OSCC. Targeting glycolytic signaling has been shown to effectively sensitize resistant cancer cells to chemotherapy. A significant enhancement in CDDP‐induced cytotoxicity was observed when ALC1 was silenced in ESCC cells, accompanied by increased apoptosis and reduced viability. This effect was attributed to the suppression of glycolysis, as evidenced by decreased glucose consumption and lactate production, and was mechanistically linked to downregulation of the PI3K/AKT. The reversal of these effects upon pathway reactivation, as well as the mitigation of ALC1 overexpression outcomes through PI3K inhibition or glycolysis blockade, further substantiated this mechanism. Notably, no such effects were observed in normal esophageal epithelial cells, suggesting a potential therapeutic window [141]. These results suggest that interfering with glycolysis via ALC1 modulation may potentiate chemotherapeutic efficacy, offering a strategy for overcoming resistance through PI3K/AKT targeting. Therefore, the PI3K/AKT pathway has potential in the regulation of glycolysis and can affect related pathways and molecular factors, including HK and LDH. Future studies can focus on targeting the PI3K/AKT pathway to regulate glycolysis and energy metabolism in tumor cells.

##### 2.3.5.2 Therapeutic Implications of Targeting PI3K/AKT‐Driven Glycolysis

Dihydroartemisinin (DHA) was found to significantly inhibit the proliferation of LNCaP cells in a dose‐dependent manner and induce apoptosis. Gene expression analysis revealed that DHA downregulated the expression of key glycolytic enzymes and affected pathways related to the cell cycle, DNA replication, and metabolism. DHA treatment decreased glucose uptake, lactate production, and ATP content in LNCaP cells, indicating glycolysis inhibition. DHA reduced the expression of glycolytic regulatory proteins such as GLUT1, HK2, PFKP, PKM2, and LDH, and downregulated HIF‐1α through the PI3K/AKT pathway. These findings suggest that DHA's anticancer effects on PCa cells may be mediated through glycolysis inhibition and modulation of the PI3K/AKT pathway [142]. The present section emphasizes the role of the PI3K/AKT pathway in regulating glycolysis, particularly in cancer metabolism where tumor cells rely on glucose metabolism for ATP production. Various studies have highlighted that upregulation of the PI3K/AKT/mTOR pathway can enhance glucose uptake, lactate generation, and the expression of essential glycolytic enzymes (HK2, LDHA, PFKFB3), which occur as a result of induction by downstream factors including HIF‐1α. Molecular factors such as PTPRH, CPNE1, APLN, PLOD1, SPAG4, B7‐H3, and SIK2 have been shown to facilitate carcinogenesis and glycolysis via PI3K/AKT induction, whereas inhibitors of this pathway reduce glycolytic flux, diminish tumor growth, and increase sensitivity to anticancer drugs. Notably, noncoding RNAs such as miR‐19a‐3p and circ‐100395 also control glycolysis through this pathway, increasing pathway complexity and highlighting the role of epigenetic factors. More studies are required to elucidate the presence of feedback loops. Additionally, the development of small molecules and natural products targeting the PI3K/Akt pathway to regulate glycolysis should be highlighted.

#### 2.3.6 PI3K/AKT/mTOR and Lipid Metabolism

##### 2.3.6.1 Mechanistic Regulation of Lipid Metabolism by PI3K/AKT/mTOR in Cancer

Metabolism is related to the material and energy activities and is vital for living organisms. There are changes in the metabolism of cancer cells to accelerate their proliferation and malignancy. Although the main focus of studies has been on the dysregulation of glycolysis and glutamine‐dependent pathways, lipid metabolism also plays a significant role in tumorigenesis. Lipids consist of triglycerides, phospholipids, cholesterol, and cholesterol esters, and they are vital for cellular functions. The process of lipid metabolism is considered a factor in the production, storage, and degradation of lipids. Phospholipids are considered the main components of the cell membrane. There is an increase in lipid metabolism to meet the energy requirements of cancer cells. Such metabolic adaptation is considered vital for membrane biogenesis, energy storage, signaling molecules, and ATP generation under energy stress conditions [143]. This understanding emphasizes the importance of lipid metabolism in supporting rapid cancer cell growth and survival, pointing to its potential as a therapeutic target in metabolic intervention strategies. GLMP is a possible regulator of lipid metabolism and is transcriptionally suppressed by BRG1. In HCC cells, knockdown of BRG1 leads to GLMP upregulation, decreased lipid droplet formation, and disruption of the PIK3AP1/PI3K/AKT axis. Importantly, suppressing GLMP under these conditions overcomes certain alterations, specifically by increasing lipid droplet generation and modulating the PI3K/AKT pathway [144]. These findings highlight GLMP as a key intermediary in lipid metabolic regulation via the PI3K/AKT pathway, with BRG1 acting as an upstream modulator, offering new avenues for metabolic targeting in HCC. The majority of cancer cells demonstrate an enhanced capacity for de novo lipid synthesis, distinguishing them from normal cells. Therefore, lipid metabolism can also be used as a biomarker. However, the underlying mechanisms have not been fully understood. Oncogenic variants of PI3K (H1047R) and K‐Ras (G12V) increase lipogenesis in breast epithelial cells through the induction of mTORC1. This activation stimulates sterol regulatory element‐binding proteins (SREBP1 and SREBP2), which are vital for lipid production and the proliferation of oncogene‐expressing cells independent of growth factors. Furthermore, mTORC1 upregulation is associated with increased expression of SREBP target genes in human breast cancer specimens [145]. This evidence supports the notion that oncogene‐driven PI3K and mTORC1 activation directly promote lipogenesis, reinforcing the role of SREBP‐driven lipid metabolism in tumor development and progression.

MIEF2 is a crucial regulator of mitochondrial fission and has been associated with worse outcomes in OC. It is considered to accelerate dysregulated lipid metabolism in this tumor. MIEF2 does not substantially affect fatty acid uptake or oxidation; however, it significantly facilitates de novo fatty acid and cholesterol synthesis by upregulating SREBP1 and SREBP2, along with their associated lipogenic target genes, including ACC1, FASN, SCD1 (for fatty acids), and HMGCS1, HMGCR (for cholesterol). This metabolic reprogramming is facilitated by increased mitochondrial ROS generation, which activates the AKT/mTOR pathway, thereby enhancing SREBP activity. MIEF2‐mediated lipid production substantially enhances OC proliferation and metastasis [146]. These results reveal a critical link between mitochondrial dynamics and lipid metabolic reprogramming in ovarian cancer, with MIEF2‐mediated activation of the PI3K/AKT/mTOR/SREBP axis driving tumor aggressiveness. Salt‐inducible kinase 2 (SIK2) has been identified for its role in modulating cellular metabolism and is a significant enhancer of lipid production in OC. SIK2 not only enhances fatty acid oxidation in adipocyte‐affected OC survival but also accelerates de novo lipid synthesis. SIK2 specifically upregulates SREBP1c and its downstream target FASN to increase fatty acid synthesis, while also inducing SREBP2 to increase transcription of HMGCR. These effects are triggered via the PI3K/AKT pathway. Functional studies demonstrate that SIK2‐mediated lipid synthesis is vital for OC cell proliferation [147]. Collectively, these insights confirm SIK2 as a central regulator of lipid biosynthesis in ovarian cancer, further validating the PI3K/AKT axis as a pivotal node in tumor‐associated metabolic reprogramming.

##### 2.3.6.2 Therapeutic Implications of Targeting PI3K/AKT‐Driven Lipid Metabolism

The current section highlights the importance of lipid metabolism in carcinogenesis, emphasizing its role in tumorigenesis similar to glycolysis and glutaminolysis. Lipid metabolism supports proliferation, membrane synthesis, energy storage, and signaling in tumor cells, primarily through enhanced de novo lipogenesis and fatty acid oxidation. The PI3K/AKT/mTOR pathway is a key regulator of lipid metabolism, stimulating the activity of SREBPs and regulating the transcription of lipogenic and cholesterogenic enzymes such as ACC1, FASN, SCD1, and HMGCR. Oncogenic drivers (PI3K H1047R, KRAS G12V), metabolic regulators (MIEF2 and SIK2), and chromatin remodeling factors (BRG1 via GLMP regulation) have been shown to affect lipid metabolism through the PI3K/AKT/mTOR pathway. These alterations not only increase proliferation and metastasis but also present therapeutic opportunities for targeting lipid metabolic pathways. However, there are limitations in understanding the differences in lipid‐related mechanisms across various tumors. More research is needed to elucidate the context‐dependent functions of PI3K/AKT/mTOR‐related lipid metabolism. Furthermore, the development of selective inhibitors to suppress this lipid metabolism pathway and reduce tumorigenesis is warranted.

### 2.4 Noncoding RNAs Regulating PI3K/AKT/mTOR Axis in Cancer

#### 2.4.1 microRNAs

The microRNAs (miRNAs) are among the short RNA molecules lacking capacity in coding and contributing to the regulatory mechanisms including expression regulation. The function of miRNAs is related to the binding to 3’‐UTR of target mRNAs to degrade them or suppress translation. The dysregulation of miRNAs has been observed in the various kinds of tumors and they have close association with PI3K/AKT axis. Significant inhibitory effects on cell proliferation and metastasis have been demonstrated through the upregulation of miR‐137. Specifically, a notable inverse correlation with Notch1 was established, whereby miR‐137 directly bound and down‐modulated Notch1, subsequently suppressing the PI3K/AKT/mTOR pathway. The malignant phenotype induced by miR‐137 deletion was partially reversed via Notch1 inhibition, underscoring its critical role in the regulatory mechanism. Enhanced expression of Notch1 and downstream pathway activation were observed upon miR‐137 suppression, while coinhibition of Notch1 attenuated this effect. Thus, the miR‐137/Notch1/PI3K/AKT/mTOR axis has been identified as a key modulator of CC cell behavior [148]. These findings highlight miR‐137 as a crucial suppressor of CC progression through its negative regulation of Notch1 and the PI3K/AKT/mTOR pathway, revealing a potential therapeutic axis for targeted intervention. A critical regulatory pathway has been identified in which GC cell proliferation and metastasis were significantly suppressed. This suppression was mediated by the upregulation of miR‐30e‐3p, which was shown to directly target and downregulate THOC2. Reduced THOC2 expression subsequently led to the inhibition of the PI3K/AKT/mTOR signaling pathway, a key axis in cancer cell survival and progression. Furthermore, low miR‐30e‐3p expression levels were associated with poor prognosis, while its overexpression hindered tumor growth both in vitro and in vivo. These findings suggest that targeting the miR‐30e‐3p/THOC2/PI3K/AKT/mTOR axis could offer therapeutic value in GC management [149]. This evidence emphasizes the tumor‐suppressive role of miR‐30e‐3p in GC and supports its use as a biomarker and therapeutic agent via modulation of the THOC2/PI3K/AKT/mTOR. A compelling mechanism of tumor suppression has been revealed, wherein miR‐5195‐3p was shown to inhibit melanoma cell proliferation and migration through direct downregulation of PCBP2, a known activator of the PI3K/AKT. Significantly reduced miR‐5195‐3p levels and elevated PCBP2 expression were detected in melanoma tissues, with a clear inverse correlation between the two. The miR‐5195‐3p overexpression suppressed tumor cell behaviors, effects that were partially reversed by PCBP2 reintroduction. Furthermore, PCBP2‐driven activation of the PI3K/AKT pathway was effectively neutralized by miR‐5195‐3p or pharmacologic inhibition, highlighting this miRNA‐pathway axis as a promising therapeutic target [150]. A compelling association between elevated miR‐589 expression and increased tumor aggressiveness has been demonstrated. Upregulation of miR‐589 has been linked with enhanced GC cell migration, invasion, and metastasis both in vitro and in vivo. A direct regulatory effect on LIFR has been established, resulting in the activation of the PI3K/AKT pathway and subsequent c‐Jun expression. Furthermore, c‐Jun has been shown to bind the promoter of miR‐589, amplifying its transcription and forming a self‐sustaining feedback loop. This miR‐589/LIFR/PI3K/AKT/c‐Jun axis has been found to drive metastatic behavior and may represent a promising molecular target for therapeutic intervention in GC [151].

A significant inhibitory effect on BC progression has been demonstrated through the upregulation of miR‐18a‐5p, which was shown to be downregulated in HER2+ BC tissues and cell lines. Suppression of proliferation, adhesion, and migration of BC cells was achieved via miR‐18a‐5p overexpression, accompanied by inactivation of the PI3K/AKT. HER2 expression was directly targeted and reduced, which in turn neutralized the oncogenic effects of HER2 overexpression, including the activation of downstream signaling. Tumor growth was significantly decreased in vivo, and molecular assays confirmed the inverse relationship between miR‐18a‐5p and HER2 expression. These findings highlight the potential therapeutic value of modulating the miR‐18a‐5p–HER2 axis in HER2+ BC [152].

A significant inhibitory effect on CC progression has been demonstrated through the upregulation of miR‐125. Specifically, reduced proliferation, migration, and invasion were observed in cancer cells, while tumor growth was also impeded in vivo. This suppressive influence was exerted via direct targeting of VEGF, whose expression was found elevated in CC and inversely correlated with miR‐125 levels. Furthermore, the activation of the PI3K/AKT pathway, known for its role in cell survival and tumor progression, was attenuated, along with a reduction in EMT markers. These findings underscore the role of miR‐125 as a tumor suppressor mediated through the VEGF/PI3K/AKT axis [153]. These results support the role of miR‐125 as a tumor suppressor in CC through inhibition of VEGF and the PI3K/AKT pathway, with implications for both prognosis and therapy. A significant inhibitory role in tumor progression has been demonstrated through the restoration of miR‐489 expression, which markedly suppressed cell viability, invasion, and migration in GC. Notably, miR‐489 was found to be downregulated, correlating with poor OS and PFS. HDAC7 was identified as a direct downstream target, and its silencing reversed the tumor‐promoting effects triggered by miR‐489 inhibition. Furthermore, modulation of the PI3K/AKT pathway was observed via the miR‐489/HDAC7 axis, leading to alterations in EMT‐related markers and downstream signaling molecules. These molecular interactions collectively highlight the tumor‐suppressive function of miR‐489 and its regulatory control over oncogenic pathways [154]. These molecular insights highlight miR‐489 as a critical regulator of tumor suppression in gastric cancer, acting through the HDAC7/PI3K/AKT axis to modulate EMT and metastatic behavior. In addition, there has been a reduction in the level of miR‐125b‐5p in BCa tissues and cell lines, providing worse prognosis and more aggressive characteristic. The miR‐125b‐5p levels suppressed survival and migration, but caused cell death. The miR‐125b‐5p is able to regulate HK2 in regulating BCa progression. In addition, miR‐125b‐5p suppresses PI3K/AKT/mTOR for exerting its function [155]. These findings suggest that miR‐125b‐5p impairs BCa progression by targeting HK2 and downregulating the PI3K/AKT/mTOR pathway, highlighting its therapeutic promise in aggressive tumors. Overexpression of miR‐19a has been strongly linked to enhanced metastatic potential and poor prognosis. Significantly elevated levels of miR‐19a were observed in tumor tissues and cell lines compared with controls, correlating with LN metastasis and advanced TNM stages. Cell proliferation, migration, and invasion were markedly increased upon miR‐19a upregulation. EMT was induced, as evidenced by morphological changes and altered expression of key markers, including reduced E‐cadherin and elevated vimentin, ZEB1, ZEB2, and Slug. Activation of the PI3K/AKT pathway was identified as the underlying mechanism, with its inhibition effectively reversing the EMT phenotype and associated molecular changes [156]. The oncogenic role of miR‐19a in promoting EMT and metastasis through activation of the PI3K/AKT pathway positions it as a potential biomarker and target in managing advanced malignancies. There are low levels of miR‐22 and high expression of NLRP3 in OC tissues and cells, demonstrating a link between downregulated miR‐22 and a negative prognosis. Furthermore, miR‐22 disrupted proliferation and EMT by binding to NLRP3 mRNA and suppressing the PI3K/AKT pathway [157]. These results establish a mechanistic link between reduced miR‐22 expression and enhanced tumor progression via NLRP3 and PI3K/AKT, suggesting therapeutic potential in ovarian cancer. A significant inhibitory effect on tumor progression was observed through the modulation of a key oncogenic pathway. miR‐1246 was found to be markedly downregulated, while its overexpression suppressed cell proliferation, migration, invasion, and enhanced apoptosis. This effect was mechanistically linked to the direct targeting of PIK3AP1, resulting in reduced PI3K/AKT phosphorylation. The suppressive impact of miR‐1246 on cellular malignancy was reversed by PIK3AP1 overexpression or activation of the PI3K/AKT pathway via IGF‐1, underscoring a negative regulatory relationship. Furthermore, in vivo experiments confirmed that miR‐1246 significantly reduced tumor growth and lung metastasis, positioning it as a promising therapeutic target [158]. These data reinforce the tumor‐suppressive capacity of miR‐1246 via direct targeting of PIK3AP1, offering a promising route for therapeutic development against PI3K/AKT‐driven cancers.

This section highlighted the importance of miRNAs as epigenetic factors involved in posttranscription regulation as regulator of tumorigenesis through affecting PI3K/AKT to control proliferation, metastasis, and survival of cancer cells. A number of miRNAs, such as miR‐181d, miR‐137, miR‐30e‐3p, and miR‐125b‐5p have been demonstrated to suppress tumorigenesis and they can affect mRNA and expression of PI3K/AKT to diminish proliferation, metastasis, and EMT with capacity of increasing apoptosis. In contrast, miRNAs such as miR‐19a and miR‐589 function as oncogenes, upregulating PI3K/AKT and developing aggressive cancer characteristics. A variety of miRNAs demonstrate interaction with certain targets including PTEN, THOC2, PCBP2, HER2, VEGF, HK2, and NLRP3, thereby associating miRNA dysregulation with essential cancer‐related pathways. The dual function of miRNAs is either inhibiting or facilitating carcinogenesis according to the context and provides a problematic condition. A significant limitation is the poor understanding of the extensive biological interactions affected by specific miRNAs and their associations with tumor heterogeneity and therapeutic resistance.

#### 2.4.2 Long Noncoding RNAs

Long noncoding RNAs (lncRNAs) are another member of noncoding RNA molecules with a linear structure and length of more than 200 nts. In the recent years, the function of lncRNAs in the regulation of tumorigenesis has been of importance and it is considered as a diagnostic and therapeutic factors in solid and hematological tumors. There is a close connection between lncRNAs and PI3K/AKT axis in cancers. LOC101928316 expression demonstrates reduction in both GC tissues and cell lines affecting TNM stage and differentiation level. The LOC101928316 upregulation suppressed the migration, invasion, and proliferation of SGC‐7901 cells through suppressing PI3K/AKT/mTOR [159]. These findings underscore the tumor‐suppressive role of LOC101928316 through inhibition of PI3K/AKT/mTOR, offering a potential diagnostic and therapeutic target in gastric cancer. PCAT7 expression increases in breast tumor and it stimulates ErbB/PI3K/AKT pathway, decreasing apoptosis and accelerating proliferation and metastasis [160]. This highlights PCAT7 as an oncogenic lncRNA promoting breast cancer progression via PI3K/AKT activation, suggesting its involvement in cell survival and metastatic potential. A strong link between elevated lncRNA levels and lung cancer aggressiveness has been established. Increased expression of LASTR has been associated with reduced survival and more advanced clinical features. Its knockdown has been shown to inhibit proliferation and metastasis of cancer cells, suggesting its oncogenic role. Functional assays revealed that LASTR exerts this effect by acting as a ceRNA, sponging miR‐137, which in turn regulates TGFA expression. This interaction ultimately activates the PI3K/AKT pathway, a well‐known driver of tumor progression. These findings highlight a mechanistic axis involving LASTR, miR‐137, and TGFA, culminating in PI3K/AKT signaling activation [161]. The elucidation of the LASTR/miR‐137/TGFA axis provides insight into the ceRNA‐mediated regulation of PI3K/AKT and identifies a novel regulatory network driving lung cancer aggressiveness.

Significant suppression of proliferative, migratory, and invasive behaviors in CC cells has been observed following the upregulation of lncRNA–CASC7. This regulatory molecule has been shown to attenuate the expression of key proliferation‐associated proteins such as Ki‐67 and PCNA, as well as invasion‐related markers including N‐cadherin and vimentin, while enhancing E‐cadherin expression. Additionally, the PI3K/AKT pathway, a critical mediator of oncogenic activity, has been significantly inactivated through decreased levels of phosphorylated PI3K and AKT, without affecting total protein levels. These findings underscore the role of lncRNA–CASC7 as a functional tumor suppressor via its negative modulation of the PI3K/AKT axis [162]. These results support the function of lncRNA–CASC7 as a tumor suppressor in CC, primarily through its inhibitory influence on EMT and PI3K/AKT‐mediated oncogenic signaling.

A significant oncogenic role was identified for lncRNA HOXB–AS3 in lung cancer progression. Elevated expression levels were associated with increased cell proliferation, enhanced colony formation, and suppressed apoptosis. Upon silencing, cells exhibited G1 phase arrest and reduced S phase distribution, indicating disrupted cell cycle progression. Migration and invasion abilities were significantly diminished, highlighting the impact on metastatic potential. Mechanistically, this effect was mediated through inactivation of the PI3K/AKT pathway, as evidenced by reduced phosphorylation levels of AKT and PI3K components. In vivo, tumor growth was also substantially inhibited, underscoring the therapeutic relevance of targeting HOXB–AS3 [163]. These findings establish HOXB–AS3 as a promoter of lung cancer progression through PI3K/AKT signaling, with its silencing presenting a viable strategy to hinder tumor growth and metastasis. The lncRNA RHPN1–AS1 is upregulated in PCa and enhances carcinogenesis by suppressing miR‐7‐5p, enhancing EGFR levels, and inducing PI3K/AKT/mTOR. RHPN1–AS1 downregulation suppresses growth and invasion and triggers cell death. miR‐7‐5p upregulation or EGFR downregulation can enhance autophagy and apoptosis in prostate tumor. Furthermore, RHPN1–AS1 stimulates PI3K/AKT/mTOR, suppresses autophagy, and decreases cell death that can be abrogated by PI3K blockage [164]. This regulatory axis involving RHPN1–AS1, miR‐7‐5p, and EGFR underscores the importance of lncRNA‐mediated control of PI3K/AKT/mTOR in PCa and its impact on autophagy and apoptosis. A significant role in BC progression has been attributed to the dysregulation of lncRNA ADAMTS9–AS1. Increased expression of this lncRNA has been associated with enhanced cell proliferation, migration, and invasion, while concurrently suppressing apoptosis and autophagy. These effects have been linked to the activation of the PI3K/AKT/mTOR signaling pathway, suggesting a key regulatory mechanism. Furthermore, reduced expression of ADAMTS9–AS1 has led to opposite outcomes, including diminished tumor cell aggressiveness and heightened cell death. Protein expression analyses confirmed these functional effects, particularly through modulation of EMT markers and autophagy‐related proteins [165]. These results confirm the role of ADAMTS9–AS1 in promoting breast cancer cell survival and invasion via activation of the PI3K/AKT/mTOR axis, reinforcing its potential as a therapeutic target in aggressive tumors. TM4SF1–AS1 upregulation were observed in metastatic lung cancer cells, particularly in those with LN metastasis. TM4SF1–AS1 overexpression enhanced cancer migration and entry, whereas reducing its levels had the opposite outcome. TM4SF1–AS1 stimulated PI3K/AKT [166]. These findings suggest that TM4SF1–AS1 plays a critical role in driving metastatic behavior in lung cancer by activating PI3K/AKT, offering a new angle for therapeutic intervention. LncRNA DLEU2 is highly expressed in GC and is associated with poor tumor differentiation, elevated CA19‐9, and specific histologic classifications. Functional assays showed that silencing DLEU2 reduced GC cell viability, migration, invasion, and induced apoptosis, while also inhibiting the PI3K/AKT pathway. Mechanistically, DLEU2 promotes GC progression and EMT by downregulating E‐cadherin and upregulating N‐cadherin and vimentin via activation of the PI3K/AKT axis. These findings suggest that DLEU2 contributes to GC development by enhancing tumor aggressiveness through the PI3K/AKT‐mediated EMT pathway [167]. These data establish DLEU2 as a significant contributor to gastric cancer progression, acting through PI3K/AKT‐mediated EMT modulation and enhancing tumor cell invasiveness and poor differentiation.

The present section provided essential roles of lncRNAs in the regulation of tumorigenesis from the aspect that is related to PI3K/AKT. LncRNAs can act as both oncogenic and onco‐suppressor factors and if the function of PI3K/AKT as autophagy regulator (dual function) is considered, the complexity increases. A number of lncRNAs, including LOC101928316, CASC7, and DLEU2, have demonstrated the capacity to suppress growth and metastasis through the downregulation of PI3K/AKT/mTOR, while others, such as PCAT7, MALAT1, SNHG20, and TM4SF1–AS1, can accelerate proliferation and migration through induction of this pathway. LncRNAs are able to function as ceRNA or they can directly affect PI3K/AKT. Although there is increasing evidence demonstrating their oncogenic or tumor‐suppressive functions, the biological roles of several lncRNAs have not been fully understood, and their tissue‐specific functions provide further complication. A limitation is related to the lack of knowledge about the underlying mechanisms based on tumor stage and type, reducing practical application of lncRNA‐based therapeutics.

#### 2.4.3 Circular RNAs

Circular RNAs (circRNAs) have been emerged as new kinds of RNA molecules with a stable structure that can be used as diagnostic factors. The circular structure of circRNAs improves their stability in blood circulation and resistance to degradation by enzymes, acting as reliable biomarkers. The function of circRNAs in the regulation of PI3K/AKT axis has been evaluated significantly, highlighting its crucial function in tumorigenesis. A strong correlation has been established between increased circ‐001422 expression and enhanced malignancy in OS. Marked upregulation of circ‐001422 has been consistently observed in both clinical OS samples and cell lines, correlating with advanced tumor stage, larger size, metastasis, and reduced patient survival. Inhibition of circ‐001422 has been shown to suppress proliferation and migration, induce apoptosis, and reduce tumor burden in vivo, while its overexpression produces the opposite effects. Mechanistically, malignant progression is driven through the sponging of miR‐195‐5p, leading to upregulation of FGF2 and activation of the PI3K/AKT pathway. These findings underscore the oncogenic role of circ‐001422 via modulation of the miR‐195‐5p/FGF2/PI3K/AKT axis [168]. These insights establish circ‐001422 as a crucial oncogenic driver in OS progression via the miR‐195‐5p/FGF2/PI3K/AKT axis, highlighting its value as a therapeutic target. A critical regulatory role has been identified in PCa progression, wherein the overexpression of has‐circ‐0030586 was shown to promote EMT through activation of the PI3K/AKT. Upon silencing this circRNA, notable suppression of cell proliferation, migration, invasion, and tumorigenesis was observed, along with upregulation of E‐cadherin and downregulation of p‐AKT, IKKα, PIK3CB, and Twist. These inhibitory effects were reversed when a miR‐145‐3p inhibitor was introduced, indicating a ceRNA mechanism involving miR‐145‐3p. In vivo assays further supported these findings, demonstrating reduced tumor growth and EMT suppression following has‐circ‐0030586 knockdown [169]. The discovery of the has‐circ‐0030586/miR‐145‐3p/PI3K/AKT axis provides a novel mechanism contributing to EMT and tumor progression in PCa, with potential for therapeutic modulation. A significant molecular mechanism contributing to breast cancer progression has been delineated, revealing that aggressive cellular behaviors are enhanced via a specific regulatory axis. Elevated levels of circ‐PRMT5 have been linked to poor patient prognosis, with its knockdown shown to inhibit proliferation, reduce angiogenesis, and promote apoptosis in cancer cells. Mechanistically, circ‐PRMT5 functions as a molecular sponge for miR‐509‐3p, thereby relieving repression on TCF7L2, which in turn activates the PI3K/AKT. This pathway is known for promoting oncogenic traits, and its activation has been confirmed to reverse the suppressive effects induced by circ‐PRMT5 silencing. These findings underscore a crucial posttranscriptional regulatory circuit that exacerbates malignancy via the circ‐PRMT5/miR‐509‐3p/TCF7L2 axis [170].

Significant upregulation of has‐circ‐0008234 was observed in colon cancer tissues and cells, and its knockdown led to reduced proliferation, invasion, and migration in vitro and in vivo. It was determined that has‐circ‐0008234 functions primarily in the cytoplasm as a competing endogenous RNA, acting as a molecular sponge for miR‐338‐3p, thereby increasing ETS1 expression. Subsequently, the PI3K/AKT/mTOR was identified as a downstream effector of the has‐circ‐0008234/miR‐338‐3p/ETS1 axis, contributing to enhanced tumor aggressiveness. Inhibition of has‐circ‐0008234 resulted in suppressed tumor growth and metastasis in mouse models, supporting its potential as a therapeutic target in colon cancer [171]. The has‐circ‐0008234/miR‐338‐3p/ETS1 axis offers further evidence of circRNA‐mediated control of the PI3K/AKT/mTOR pathway, reinforcing its significance in cancer progression. Transfection with pcDNA–has‐circ‐0000520 decreased viability and increased apoptosis when exposed to herceptin in comparison with the control group. Downregulation of Bcl‐2, p‐PI3K, and p‐AKT were highlighted when has‐circ‐0000520 was upregulated. Treatment with IGF‐1 enhanced survival and reduced apoptosis. The hsa‐circ‐0000520 downregulation was related to resistance to herceptin in GC, whereas upregulation of hsa‐circ‐0000520 enhanced sensitivity to herceptin by affecting the PI3K/AKT [172]. These findings indicate that has‐circ‐0000520 can enhance sensitivity to herceptin in gastric GC by modulating PI3K/AKT activity, suggesting its potential as a therapeutic enhancer. Elevated expression of has‐circ‐001569 has been linked to enhanced tumor aggressiveness and poor prognosis. Significant upregulation was observed in both BC tissues and cell lines, correlating with advanced clinical stage, LN metastasis, and reduced overall survival. Cellular assays revealed that silencing of hsa‐circ‐001569 markedly suppressed proliferation, migration, and invasion, while inducing apoptosis. EMT markers such as N‐cadherin and vimentin were also downregulated upon knockdown. Furthermore, reduced activation of the PI3K/AKT pathway was detected, indicating a mechanistic link to tumor progression. These findings highlight hsa‐circ‐001569 as a potential oncogenic driver and independent prognostic indicator in BC [173]. These observations identify hsa‐circ‐001569 as a potent oncogenic factor in breast cancer, acting through PI3K/AKT and EMT regulation to promote malignancy.

**FIGURE 6:**
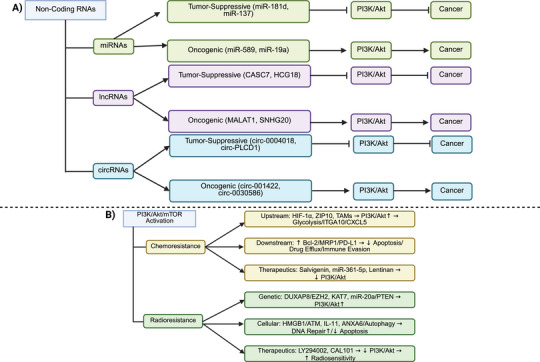
(A) There are different kinds of ncRNAs with both oncogenic and onco‐suppressor functions regulating PI3K/AKT/mTOR axis. The miRNAs can be sponged by circRNAs and lncRNAs to further affect PI3K/AKT expression. These interactions can finally regulate cancer hallmarks including proliferation, metastasis, and drug resistance. (B) The involvement of PI3K/AKT in the drug resistance and radioresistance. It can regulate glycolysis, apoptosis, and immune evasion in chemoresistance with therapeutics such as salvigenin and others capable of downregulating PI3K/AKT. In radioresistance, PI3K/AKT regulates autophagy, DNA repair, and apoptoss in which CAL101 and LY294002 can suppress PI3K/AKT in enhancing response to radiotherapy; (created with Biorender.com).

A compelling regulatory axis involving circ‐0102231, miR‐635, and NOVA2 has been revealed to significantly influence the malignant behavior of NSCLC cells. Elevated levels of circ‐0102231 have been associated with advanced clinical features, while its silencing has been shown to suppress proliferation and angiogenesis and induce apoptosis. Mechanistically, circ‐0102231 has been found to act as a molecular sponge for miR‐635, thereby preventing the miRNA‐mediated downregulation of NOVA2. Consequently, NOVA2 expression is enhanced, contributing to tumor progression. Notably, the circ‐0102231/miR‐635/NOVA2 axis has been demonstrated to modulate the PI3K/AKT, with its inactivation resulting in pronounced antitumor effects both in vitro and in vivo [174]. The circ‐0102231/miR‐635/NOVA2 regulatory axis reveals a functional role for circRNAs in NSCLC progression through modulation of the PI3K/AKT pathway, offering a new therapeutic angle. Activated by p53, circ‐PLCD1 suppressed the growth and metastasis of NSCLC cells while also elevating apoptosis. The circ‐PLCD1 acted as a sponge for miR‐375 and miR‐1179 in order to increase PTEN levels, thereby suppressing the PI3K/AKT and decreasing NSCLC progression [175]. These results position circ‐PLCD1 as a tumor suppressor in NSCLC via PTEN‐mediated inhibition of PI3K/AKT, illustrating the impact of p53‐regulated circRNAs on tumor control. A strong oncogenic role has been attributed to hsa‐circ‐0003596 in ccRCC progression. Its overexpression has been linked to enhanced cellular proliferation, infiltration, migration, and distant metastasis. In vitro and in vivo silencing of this circRNA led to a marked suppression of tumor growth and metastatic potential. Mechanistically, a ceRNA regulatory axis was elucidated, whereby hsa‐circ‐0003596 acts as a sponge for miR‐502‐5p, thereby alleviating its inhibition of IGF1R. This, in turn, activated the PI3K/AKT pathway, which is known for promoting malignancy. The findings suggest that this circRNA/miRNA/mRNA cascade significantly contributes to the malignant phenotype and may serve as a valuable diagnostic or therapeutic target in ccRCC [176]. Altogether, these findings point to hsa‐circ‐0003596 as a key regulator of ccRCC malignancy through the miR‐502‐5p/IGF1R/PI3K/AKT axis, suggesting diagnostic and therapeutic potential. Table 3 demonstrates the function of noncoding RNAs in the regulation of PI3K/AKT in cancers, summarized in Figure 6. Table 4 further modesntrates the function of PI3K/AKT in therapy resistance.

**TABLE 3:** The regulation of PI3K/AKT axis by noncoding RNAs in cancers.

Noncoding RNA	Cancer	Remark	References
Circ‐0002496	Breast cancer	Increase in the stemness and angiogenesis through induction of PI3K/AKT Regulation of miR‐433‐3p and YWHAZ	[177]
Circ‐0008285	Colorectal cancer	Downregulating miR‐382‐5p to increase PTEN to impair PI3K/AKT	[178]
Circ‐0007503	Breast cancer	Eriodictyol impairs tumorigenesis and downregulates PI3K/AKT Downregulation of circ‐0007503	[179]
Circ‐0001313	Colon cancer	Sponging miR‐510‐5p to increase AKT2 expression	[180]
Circ‐0030586	Prostate cancer	Upregulation of PI3K/AKT to induce EMT	[169]
Circ‐0000520	Gastric cancer	Suppressing herceptin resistance through PI3K/AKT downregulation	[172]
Circ‐001422	Osteosarcoma	Sponging miR‐195‐5p to induce FGF2/PI3K/AKT axis	[168]
Has‐circ‐0000520	Bladder cancer	PTEN upregulation and downregulation of PI3K/AKT	[181]
Circ‐PRMT5	Breast cancer	Sponging miR‐509‐3p to increase TCF7L2 levels in the induction of PI3K/AKT	[170]
Circ‐100395	Thyroid cancer	Suppression of PI3K/AKT/mTOR to reduce progression and glycolysis	[136]
Circ‐0004018	Hepatocellular carcinoma	Sponging miR‐1197 to increase PTEN levels in the suppression of PI3K/AKT	[182]
Circ‐0008234	Colon cancer	Sponging miR‐338‐3p and upregulating ETS1 Induction of PI3K/AKT/mTOR axis	[171]
Circ‐0000442	Breast cancer	Sponging miR‐148b‐3p to suppress PI3K/AKT	[183]
Circ‐0085121	Prostate cancer	Upregulation of PI3K/AKT/mTOR Increasing AR‐V7 alternative splicing	[184]
Circ‐PPAPDC1A	Lung cancer	Increasing osimertinib resistance Sponging miR‐30a‐3p Induction of IGF1R/PI3K/AKT/mTOR	[185]
Has‐circ‐0001666	Lung cancer	miR‐1184/miR‐548I sponging to increase AGO1 levels in the induction of PI3K/AKT/mTOR axis	[186]
Circ‐0007386	Lung cancer	Increasing levels of YAP1–EIF4A3 by hypoxia to promote circ‐0007386 levels to compete with CRIM1 pre‐mRNA linear splicing and regulate PI3K/AKT in affecting proliferation and apoptosis	[187]
Circ‐0042881	Breast cancer	EIF4A3 enhances circ‐0042881 levels to sponge miR‐217 to inducing PI3K/AKT and MEK/ERK axis	[188]
LncRNA–HNF1A–AS1	Gastric cancer	Sponging miR‐30b‐3p to induce PI3K/AKT	[189]
miR‐195 and miR‐497	Breast cancer	Stimulation of tamoxifen resistance through induction of PI3K/AKT	[190]
CircVAPA	Lung cancer	Sponging miR‐377‐3p and miR‐494‐3p to induce PI3K/AKT	[191]
miR‐4677‐3p	Gastric cancer	Reducing CEMIP expression Stimulation of PI3K/AKT axis	[192]
miR‐203a	Esophageal cancer	SOX9 inhibited miR‐203a to induce PI3K/AKT/mTOR axis	[193]
miR‐18a‐5p	Breast cancer	Reduction in tumorigenesis through downregulating PI3K/AKT	[152]
LncRNA UCA1	Gastric cancer	Recruitment of EZH2 and induction of PI3K/AKT	[194]
LncRNA–CASC7	Colon cancer	Downregulation of PI3K/AKT to suppress growth and invasion	[162]
LncRNA RP11‐301G19.1	Myeloma	miR‐582‐5p sponging to increase HMGB2 levels and the induction of PI3K/AKT axis	[195]
LncRNA HCG18	Cholangiocarcinoma	Exosomal HCG18 sponging miR‐424‐5p in PI3K/AKT induction	[196]
LncRNA LINC01857	Lymphoma	Growth increase and apoptosis inhibition Sponging miR‐141‐3p and miR‐141‐3p Induction of PI3K/AKT	[197]
LncRNA DUXAP8	Breast cancer	Induction of PI3K/AKT to trigger radioresistance	[198]

Abbreviations: AGO1, argonaute RISC catalytic component 1; AKT2, AKT serine/threonine kinase 2; AR‐V7, androgen receptor splice variant 7; CASC7, cancer susceptibility candidate 7; Circ, circular RNA; CRIM1, cysteine‐rich transmembrane BMP regulator 1; DUXAP8, double homeobox A pseudogene 8; EIF4A3, eukaryotic translation initiation factor 4A3; EMT, epithelial–mesenchymal transition; ERK, extracellular signal‐regulated kinase; ETS1, ETS proto‐oncogene 1; EZH2, enhancer of zeste homolog 2; FGF2, fibroblast growth factor 2; FNDC3B, fibronectin type iii domain containing 3B; HCG18, HLA complex group 18; HMGB2, high mobility group box 2; HNF1A‐AS1, HNF1A antisense RNA 1; hnRNPD, heterogeneous nuclear ribonucleoprotein D; IGF1R, insulin‐like growth factor 1 receptor; LINC01857, long intergenic non‐protein coding RNA 1857; MEK, MAPK/ERK kinase; miR, microRNA; mTOR, mechanistic target of rapamycin; PTEN, phosphatase and tensin homolog; SOX9, SRY‐box transcription factor 9; TCF7L2, transcription factor 7 like 2; THOR, testis‐associated Highly‐conserved oncogenic long noncoding RNA; UCA1, urothelial cancer associated 1; YAP1, Yes‐associated protein 1; YWHAZ, tyrosine 3‐monooxygenase/tryptophan 5‐monooxygenase activation protein zeta.

**TABLE 4:** The function of PI3K/AKT axis in chemoresistance and radioresistance.

Molecular target	Cancer	Remark	References
PDK1	Hepatocellular carcinoma	Upregulation of PDK1 stimulates PI3K/AKT/mTOR axis in mediating radioresistance	[211]
G6PD	Small cell lung cancer	PI3K/mTOR enhances autophagic degradation of G6PD and promote oxidative damage in enhancing radiosensitivity	[212]
miR‐410	Non‐small cell lung cancer	miR‐410 stimulates PI3K/mTOR axis to increase EMT and radioresistance	[213]
IL‐11	Cervical cancer	IL‐11 stimulates PI3K/AKT in radioresistance development	[214]
IL‐8	Gastric cancer	Calcipotriol suppresses PI3K/AKT axis to impair the function of CAFs in the secretion of IL‐8 and mediating oxaliplatin resistance	[215]
ZIP10	Osteosarcoma	ZIP10 triggers ITGA10‐driven PI3K/AKT to enhance growth and drug resistance	[202]
ROS/PI3K/AKT and Wnt/β‐catenin	Colorectal cancer	Stimulation of HIF‐1α to accelerate metabolic reprogramming in mediating 5‐FU resistance	[201]
CircTRIM1	Triple‐negative breast cancer	Encoding TRIM1‐269aa to promote CaM‐dependent MARCKS translocation and PI3K/AKT/mTOR upregulation in drug resistance and invasion	[216]
miR‐20a	Lung cancer	The exosomal miR‐20a derived from CAFs can suppress PTEN to induce PI3K/AKT axis in enhancing tumorigenesis and drug resistance	[217]
CXCL5	Gastric cancer	CXCL5 upregulation stimulates PI3K axis to increase cancer drug resistance	[199]
MDA2	Cholangiocarcinoma	Upregulation of IGF1R/PI3K/AKT axis by MAD2 to promote tumorigenesis and suppressing USP44/LIMA complex	[218]
Hsa‐miR‐3178	Pancreatic cancer	Upregulation of PI3K/AKT axis to mediate expression of ABC transporters in gemcitabine resistance	[219]
ATXN2	Gastric cancer	ATXN2 can increase PI3K/AKT levels to mediate drug resistance and reduce the anticancer activity of T cells	[204]
FOXD1–AS1	Gastric cancer	FOXD1–AS1 stimulates PI3K/AKT/mTOR axis to enhance the interaction of eIF4E with eIF4G in drug resistance development	[200]
c‐Met	Ovarian cancer	HGF can induce c‐Met/PI3K/AKT axis to increase stemness and chemoresistance	[220]
Connexin 43	Glioblastoma	Connexin 43 upregulates PI3K to induce temozolomide resistance	[221]
FTO	Pancreatic cancer	FTO suppresses gemcitabine resistance through PTEN downregulation and inducing PI3K/AKT	[222]

Abbreviations: β‐catenin, beta‐catenin; 5‐FU, 5‐fluorouracil; ABC, ATP‐binding cassette; AKT, protein kinase B; ATXN2, ataxin 2; CAFs, cancer‐associated fibroblasts; CaM, calmodulin; CircTRIM1, circular RNA TRIM1; CXCL5, C‐X‐C motif chemokine ligand 5; eIF4E, eukaryotic translation initiation factor 4E; eIF4G, eukaryotic translation initiation factor 4 gamma; EMT, epithelial–mesenchymal transition; FOXD1–AS1, Forkhead box D1 antisense RNA 1; FTO, fat mass and obesity‐associated protein; G6PD, glucose‐6‐phosphate dehydrogenase; HGF, hepatocyte growth factor; HIF‐1α, hypoxia‐inducible factor 1‐alpha; IGF1R, insulin‐like growth factor 1 receptor; IL‐11, interleukin‐11; IL‐8, interleukin‐8; ITGA10, integrin subunit alpha 10; LIMA, LIM domain and actin binding protein complex; MARCKS, myristoylated alanine‐rich C‐kinase substrate; MDA2, mitotic arrest deficient 2; miR, microRNA; mTOR, mechanistic target of rapamycin; PDK1, 3‐phosphoinositide‐dependent protein kinase‐1; PI3K, phosphoinositide 3‐kinase; PTEN, phosphatase and tensin homolog; ROS, reactive oxygen species; TRIM1‐269aa, tripartite motif‐containing protein 1, 269 amino acid isoform; USP44, ubiquitin specific peptidase 44; Wnt, wingless/integrated signaling pathway; ZIP10, zinc transporter protein 10.

The pivotal function of circRNAs in the regulation of carcinogenesis was discussed with specific focus on the modulation of PI3K/AKT/mTOR. CircRNAs demonstrate a covalently closed‐loop structure and it demonstrates high stability, acting as diagnostic and therapeutic functions in cancers. CircRNAs can be ceRNA for sponging miRNAs to regulate expression of targets. There are oncogenic circRNAs including circ‐001422, circ‐0030586, circ‐PRMT5, and circ‐0002577 capable of accelerating proliferation, metastasis, EMT and reducing apoptosis through PI3K/AKT/mTOR induction by increasing levels of FGF2, TCF7L2, IGF1R, and ETS1. In contrast, tumor‐suppressive circRNAs, specifically circ‐0004018, circ‐0072309, circ‐0000520, and circ‐PLCD1, can diminish PI3K/AKT by upregulating PTEN or downregulating IGF1R, resulting in decreased proliferation, invasion, and increased responsiveness to treatments such as herceptin.

### 2.5 PI3K/AKT/mTOR in Cancer Drug Resistance

#### 2.5.1 Mechanistic Role of PI3K/AKT/mTOR in Drug Resistance

Chemotherapy using 5‐FU is frequently utilized for treating GC; however, resistance to this type of treatment can be difficult to overcome. The connection between chemoresistance and the TME involves the participation of tumor‐associated macrophages (TAMs). TAM density was measured using immunohistochemistry in 103 GC patients receiving 5‐FU‐based chemotherapy. Strong TAM presence was associated with resistance to chemotherapy. GC cells caused polarization of macrophages to a M2‐like phenotype, enhancing resistance to chemotherapy. PI3K/AKT/mTOR upregulation was enhanced by CXCL5 from M2‐polarized macrophages, leading to increased chemoresistance. CXCL5 also recruited monocytes to produce additional M2‐polarized macrophages [199].

A pivotal regulatory mechanism was identified in which the upregulation of lncRNA FOXD1–AS1 enhanced GC progression and chemoresistance. This effect was mediated through posttranscriptional activation of FOXD1 translation by facilitating the assembly of the eIF4G–eIF4E–eIF4A complex, achieved via phosphorylation of 4E‐BP1. Activation of the PI3K/AKT/mTOR pathway was shown to underlie this process, driven by FOXD1–AS1‐mediated upregulation of PIK3CA through miR‐466 sequestration. Suppression of FOXD1–AS1 resulted in reduced tumor growth, motility, and resistance to DDP both in vitro and in vivo, highlighting its critical oncogenic function in GC [200]. These findings establish FOXD1–AS1 as a central driver of GC progression and chemoresistance, operating through a PI3K/AKT/mTOR‐dependent mechanism involving translational regulation and miRNA sequestration.

#### 2.5.2 Tumor‐Specific Examples of PI3K/AKT‐Mediated Chemoresistance

Upon the introduction of chemotherapy, there was significant improvement in the survival and prognosis of patients. However, the cancer patients demonstrated poor response to chemotherapy after frequent and long‐term application of anticancer drugs. The combination therapy and use of nanoparticles have been suggested in the recent years as promising strategies to overcome chemoresistance. However, understanding the underlying mechanisms in the cancer drug resistance is of importance, since they can suggest new kinds of therapeutics targets. Since PI3K/AKT axis participates in the carcinogenesis, this pathway contributes to the regulation of chemoresistance that is aim of current section. Resistance to 5‐FU presents a major challenge in CRC, as targeted therapies have not been effective. Elevated HIF‐1α levels in CRC patients led to glucose reprogramming causing resistance to 5‐FU treatment, resulting in treatment failure and reduced survival rates. The increase in HIF‐1α expression was caused by PI3K/AKT and abnormal β‐catenin activation, regardless of oxygen levels [201]. These findings reveal that PI3K/AKT‐driven metabolic reprogramming, specifically via HIF‐1α upregulation, contributes significantly to 5‐FU resistance in CRC, highlighting the need for strategies targeting glucose metabolism.

In both human tumors and OS, increased levels of the zinc transporters Zrt‐ and Irt‐related protein (ZIP/SLC39) are associated with a poor prognosis, although their impact on the development of cancer and resistance to chemotherapy in OS remains uncertain. ZIP10 levels were evaluated in 64 OS patient samples, with or without chemotherapy, and how it affects OS growth and resistance to chemotherapy. The chemotherapy led to elevated levels of ZIP10 and was closely associated with patient outcomes related to overall survival. Blocking ZIP10 suppressed growth and resistance to chemotherapy, whereas increasing its levels enhanced the expression of integrin α10 (ITGA10) and activated the PI3K/AKT pathway, resulting in chemoresistance. Blocking CREB or PI3K/AKT compromised the resistance in cells overexpressing ZIP10 [202]. These results establish ZIP10 as a critical regulator of chemoresistance in OS through integrin α10 and PI3K/AKT, suggesting that disrupting this axis could improve treatment outcomes. Gas7's participation in the multiple cellular processes, especially its contribution to HCC, remained uncertain. There was a study to explore the function of Gas7 in HCC and demonstrated that it was reduced in HCC cell lines, showing a correlation between low expression and reduced patient survival. Gas7 suppressed growth and migration, caused cell cycle arrest, programmed cell death, and boosted OXA sensitivity by blocking the PI3K/AKT. Sp1 was recognized as a transcription factor inhibiting Gas7. The Sp1/Gas7/PI3K/AKT pathway was critical in the progression of HCC, migration, and response to OXA [203]. Collectively, these findings identify the Sp1/Gas7/PI3K/AKT axis as a novel suppressive mechanism in HCC, where enhancing Gas7 expression may restore OXA sensitivity and reduce malignancy.

Chemotherapy is a frequently utilized method for the treatment stomach cancer; however, its efficacy is often limited by chemoresistance. Immunotherapy is obtaining special attention as a treatment choice for patients. A research focused on evaluating the function of ATXN2 in the effectiveness of chemotherapy and immune evasion in GC cells. ATXN2 levels were higher in tumors and associated with a poor survival. It was also associated with enhancing resistance to chemotherapy by inducing PI3K/AKT and upregulating BCL2L1. Furthermore, ATXN2 increased PD‐L1 levels to improve the effectiveness of immunotherapy. SP1 regulates the ATXN2 expression and plays a role in resistance to chemotherapy and evasion of the immune system in GC [204]. This highlights ATXN2 as a dual mediator of chemoresistance and immune evasion via PI3K/AKT activation and PD‐L1 upregulation, suggesting its relevance in combination therapies for GC. The function of SRPX2 in the regulation of drug resistance in PC through PI3K/AKT/mTOR regulation was evaluated. Patients in partial remission had lower SRPX2 levels postchemotherapy compared with those with stable or progressive disease. Patients who demonstrated a good response to chemotherapy possessed decreased levels of SRPX2. Elevated levels of SRPX2 in the blood and fluctuations in SRPX2 levels were associated with a poor prognosis for PC. Downregulating SRPX2 enhanced the sensitivity of PC cell lines to chemotherapy by reducing levels of p‐PI3K, p‐AKT, and p‐mTOR. IGF‐1 therapy overcome these impacts on PC growth, migration, infiltration, and resistance to chemotherapy [205]. These insights establish SRPX2 as a modulator of drug resistance in PC through the PI3K/AKT/mTOR, positioning it as both a prognostic marker and a target for sensitizing chemotherapy.

A critical mechanism of chemoresistance in glioma has been attributed to the overexpression of TRIM31, which significantly enhances cell viability and reduces apoptosis following TMZ treatment. Increased TRIM31 expression has been linked to higher IC₅₀ values and augmented colony formation, indicating diminished sensitivity to chemotherapy. Apoptotic suppression was found to coincide with decreased p53 levels, suggesting interference with proapoptotic signaling. Mechanistically, TRIM31‐mediated resistance has been associated with the activation of the PI3K/AKT pathway, as evidenced by elevated p‐AKT levels, and this effect was abolished upon PI3K inhibition. These findings underscore TRIM31 as a potential molecular contributor to TMZ resistance via the PI3K/AKT/p53 axis [206]. These findings implicate TRIM31 in TMZ resistance via activation of the PI3K/AKT/p53 axis, providing a mechanistic basis for therapeutic resistance and a candidate target for glioma treatment enhancement.

A significant role in CDDP chemoresistance and tumor progression has been attributed to the overexpression of HPRT1 in OSCC. Elevated levels of this enzyme have been linked to poor prognosis, enhanced tumor growth, and reduced apoptosis in both in vitro and in vivo models. Chemoresistance was shown to be mediated through upregulation of MMP1 and subsequent activation of the PI3K/AKT, which contributed to cell survival and proliferation under CDDP treatment. Downregulation of HPRT1 reduced resistance, increased apoptotic responses, and impaired tumor growth, indicating that targeting this axis may enhance therapeutic efficacy and serve as a prognostic and therapeutic avenue in OSCC management [207]. The study supports HPRT1 as a key driver of chemoresistance and tumor progression in OSCC, operating through MMP1‐mediated PI3K/AKT activation, and highlights its potential as a therapeutic vulnerability.

Despite initial treatment success, SCLC often relapses with multidrug resistance. Using PDX models and proteomic profiling, researchers identified MCAM as highly upregulated in chemoresistant SCLC compared with treatment‐naïve tumors. Silencing MCAM reduced cell proliferation and enhanced chemosensitivity by lowering IC_50_ values. This effect was mediated through SOX2‐dependent activation of MRP1/ABCC1 and the PI3K/AKT pathway. Metabolomic analysis showed that MCAM influences lactate production and supports a low oxidative phosphorylation metabolic phenotype. These findings highlight MCAM as a potential therapeutic target for overcoming SCLC chemoresistance [208]. This research identifies MCAM as a pivotal regulator of chemoresistance in SCLC via SOX2/MRP1/PI3K/AKT and metabolic reprogramming, presenting a compelling case for its therapeutic targeting.

#### 2.5.3 Targeting PI3K/AKT/mTOR to Overcome Drug Resistance

Salvigenin, a trimethoxylated flavone prevalent in *Scutellariae Barbatae Herba* and *Scutellariae Radix*, has exhibited significant antitumor efficacy in colon cancer. Nonetheless, its function and underlying processes in HCC remain little investigated. This study examined the impact of different doses of salvigenin on several cellular activities in HCC cells. The therapy led to a concentration‐dependent decrease in HCC cell proliferation, migration, and invasion. Furthermore, salvigenin impeded glycolytic activity, as observed by decreased glucose absorption and lactate generation, in conjunction with the downregulation of essential glycolytic enzymes. The chemical increased the susceptibility of HCC cells to 5‐FU and reduced drug resistance in 5‐FU‐resistant HCC cells. Network pharmacological research indicated that salvigenin may influence the PI3K/AKT. Salvigenin inhibited the phosphorylation of PI3K, AKT, and GSK‐3β. Moreover, the activation of the PI3K/AKT/GSK‐3β pathway by the PI3K agonist 740Y‐P facilitated oncogenic behaviors in HCC cells; however, these effects were significantly mitigated by salvigenin therapy. In vivo tests with nude mice revealed that salvigenin significantly suppressed tumor development and promoted apoptosis in HCC cells. Salvigenin impedes aerobic glycolysis and mitigates 5‐FU resistance in HCC via inhibiting the PI3K/AKT/GSK‐3β [209]. These findings establish salvigenin as a potent therapeutic agent in HCC, capable of reversing 5‐FU resistance and impairing tumor growth through suppression of the PI3K/AKT/GSK‐3β axis and glycolytic metabolism. Pemetrexed is utilized in the treatment of NSCLC; however, its efficacy is diminished by the development of drug resistance. Lentinan found in Shiitake mushrooms has anticancer features utilized in treating lung cancer. The pemetrexed‐resistant cells were subjected to treatment with both drugs. Lentinan diminished the IC_50_ of pemetrexed, facilitated its antiproliferative effect, promoted levels of oxidative stress markers, and blocked PI3K/AKT. Activator 740Y‐P countered the effects of lentinan on proliferation and oxidative stress [210]. This evidence highlights the ability of lentinan to sensitize pemetrexed‐resistant NSCLC cells by enhancing oxidative stress and inhibiting PI3K/AKT, suggesting its potential as a combinatorial treatment strategy.

The discussions highlight the fact that upregulation of PI3K/AKT/mTOR axis frequently occurs in the different kinds of tumors. Therefore, inhibition of this axis can reduce aggressive behavior of cancer cells. More important function of PI3K/AKT axis is related to impact on chemoresistance because of controlling cellular survival, proliferation, and apoptosis mechanisms interfering with efficacy of therapeutics. Upregulation of PI3K/AKT/mTOR axis can increase the levels of MRP‐related proteins including P‐glycoprotein, leading to chemoresistance and reduction in accumulation of anticancer compounds in the tumor cells. Moreover, PI3K/AKT increases the levels of Bcl‐2 and Mcl‐1, whereas it downregulates Bad and Bax to reduce apoptosis by chemotherapy drugs. The genetic mutations such as PIK3CA mutation or PTEN loss can induce this axis to mediate chemoresistance.

### 2.6 PI3K/AKT/mTOR in Cancer Radioresistance

#### 2.6.1 Mechanistic Basis of PI3K/AKT/mTOR‐Mediated Radioresistance

A strong association has been established between elevated PDK1 expression and enhanced radioresistance in HCC cells. Activation of the PI3K/AKT/mTOR has been found to occur independently of upstream regulators and is linked to poor cellular differentiation, increased motility, invasiveness, and cancer stem cell‐like characteristics. Suppression of PDK1, either genetically or pharmacologically, has been shown to sensitize cells to IR, induce apoptosis via modulation of Bax/Bcl‐2 ratios, inhibit EMT markers, and reduce clonogenic and tumorsphere‐forming capabilities. Additionally, the interaction of PDK1 with ALDH1A1 has been implicated in the acquisition of stemness and evasion of DNA damage. These findings collectively suggest that inhibition of PDK1 could effectively reverse IR resistance and attenuate oncogenic features in aggressive HCC phenotypes [211]. These findings identify PDK1 as a critical mediator of HCC radioresistance through the PI3K/AKT/mTOR pathway, implicating it as a promising therapeutic target to sensitize tumors to radiation and inhibit stemness‐associated traits. Another research evaluated how HMGB1 affects the sensitivity of esophageal cancer cells to radiation by controlling the PI3K/AKT/ATM pathway. Analysis of HMGB1 and p‐ATM expression was performed on biopsies from patients with esophageal cancer. HMGB1 has been demonstrated to trigger radioresistance through PI3K/AKT/ATM. Patients with different levels of HMGB1 and p‐ATM expression experienced different survival outcomes. HMGB1 depletion along with ly294002 disrupted proliferation and invasion, whereas HMGB1 silencing enhanced cancer cell death postradiation, particularly in combination with ly294002 [223]. This study highlights HMGB1 as a key regulator of esophageal cancer radioresistance, acting via the PI3K/AKT/ATM axis, and suggests that its inhibition could improve radiotherapy outcomes through enhanced cell death and impaired invasion. Exosomal HMGB1 has been shown to significantly promote radioresistance in ESCC by enhancing cell proliferation, inhibiting apoptosis, and accelerating DNA damage repair. Elevated levels of HMGB1 in plasma exosomes were associated with poor response to RT, while IR was found to stimulate HMGB1 release into exosomes, which were then internalized by recipient cells. This process activated the PI3K/AKT/FOXO3A, leading to G2/M phase arrest, reduced apoptosis via regulation of Bax and Bcl2, and increased expression of γH2AX, thereby enhancing DNA damage response (DDR). Suppression of HMGB1 attenuated these effects and restored radiosensitivity [224]. These results emphasize the role of exosomal HMGB1 in promoting ESCC radioresistance by activating the PI3K/AKT/FOXO3A and facilitating DNA damage repair, positioning it as a novel biomarker and therapeutic target in radiation response modulation.

#### 2.6.2 Tumor‐Specific Evidence of PI3K/AKT‐Driven Radioresistance

A strong correlation between high lncRNA DUXAP8 expression and enhanced radioresistance in BC has been demonstrated. Increased DUXAP8 levels have been linked to poor clinical outcomes, reduced apoptosis, and decreased DNA damage postirradiation. Radioresistance has been shown to be mediated via activation of the PI3K/AKT/mTOR signaling cascade and epigenetic repression of E‐cadherin and RHOB through interaction with EZH2. Suppression of DUXAP8 or interference with these pathways has resulted in restored radiosensitivity and increased apoptotic activity. These results indicate that modulation of DUXAP8 activity could serve as a potential strategy to overcome radiotherapy resistance in BC [198]. These findings establish DUXAP8 as a critical epigenetic regulator of radioresistance in breast cancer via activation of the PI3K/AKT/mTOR pathway and repression of tumor suppressors, highlighting its potential as a therapeutic target. KAT7 enhances radioresistance in breast cancer by increasing PI3K/AKT. Elevated levels of KAT7 in breast cancer patients have a detrimental effect on survival rates. KAT7 stimulates the transcription of PIK3CA, leading to increased PI3K/AKT level and resistance to radiation. Blocking KAT7 reduces resistance to radiation, but this can be overcome by increased levels of AKT or PIK3CA. KAT7 also enhances AKT phosphorylation and radioresistance by acetyltransferase function [225]. This evidence highlights the oncogenic role of KAT7 in enhancing breast cancer radioresistance through transcriptional upregulation of PIK3CA and PI3K/AKT pathway activation, suggesting therapeutic potential in KAT7 inhibition. A significant mechanism underlying treatment resistance has been identified, where cellular radioresistance is promoted via the activation of the PTEN/PI3K/AKT. Specifically, increased miR‐20a levels have been shown to downregulate PTEN expression, resulting in heightened p‐Akt activity and reduced apoptosis in HCC cells exposed to radiation. Conversely, restoration of PTEN or inhibition of PI3K reversed this resistance, sensitizing cells to radiotherapy. These molecular interactions were consistently observed in both in vitro and in vivo models, suggesting that modulation of this axis could be leveraged to enhance therapeutic outcomes in resistant tumors [226]. These results reveal a pivotal role for the miR‐20a/PTEN/PI3K/AKT axis in driving radioresistance in HCC, with PTEN restoration or PI3K inhibition offering effective radiosensitization strategies.

IL‐11 has been identified as a key contributor to the development of radioresistance in CC cells. Elevated levels of IL‐11 were correlated with advanced tumor stages and poorer survival outcomes. Experimental downregulation of IL‐11 expression resulted in increased radiosensitivity, evidenced by reduced cell viability, enhanced apoptosis, and G2/M phase cell cycle arrest postirradiation. Conversely, exogenous IL‐11 administration significantly increased survival and reduced apoptosis in irradiated cells. Mechanistically, the PI3K/AKT pathway was found to be activated by IL‐11, and its inhibition effectively reversed IL‐11‐induced radioresistance. These findings suggest that modulation of IL‐11 and the PI3K/AKT axis could represent a promising strategy to overcome therapeutic resistance [214]. Collectively, the data demonstrate that IL‐11 promotes CC radioresistance by activating PI3K/AKT signaling, and its suppression could serve as a novel approach to enhance radiotherapy response.

Elevated levels of ANXA6 were associated with resistance to radiation, while blocking ANXA6 enhanced sensitivity to radiation. Radioresistant NPC cells demonstrated increased autophagy levels, and cells became more sensitive to radiation when autophagy was suppressed. Silencing ANXA6 diminished autophagy by activating the PI3K/AKT/mTOR pathway, resulting in increased sensitivity to radiation. Mixing ANXA6 siRNA with CAL101 effectively increased autophagy levels that were previously decreased. CAL101 elevated ANXA6 levels by inhibiting the PI3K/AKT/mTOR pathway in a negative loop [227]. These findings reveal ANXA6 as a modulator of autophagy‐driven radioresistance in NPC, acting through feedback regulation of the PI3K/AKT/mTOR pathway, and suggest its dual role as a potential biomarker and therapeutic target. There are several mechanisms participating in the induction of radioresistance by PI3K/AKT that is mediated through regulating cellular survival, increasing DNA damage repair and decreasing apoptosis after radiation therapy. Upon the upregulation of PI3K/AKT, this axis participates in the increased levels of Bcl‐2 and survivin, while reducing Bax levels to diminish cell death and enhance radioresistance. This pathway also enhances the levels of DNA damage repair by increasing levels of proteins such as DNA‐PK and Rad51 to enhance repair of double‐strand breaks. PI3K/AKT axis also participates in enhancing the cellular proliferation and improving TME for cancer progression including VEGF expression to trigger angiogenesis.

### 2.7 PI3K/AKT/mTOR and Immune Evasion

Loss of PTEN is frequently observed in cancer, where it controls PI3K and enhances tumor growth. PI3Kβ controls immune evasion in breast tumors lacking PTEN. In a mouse model of breast cancer where PTEN and Trp53 were removed, the inactivation of PI3Kβ resulted in a robust immune response against tumors in mice with a healthy immune system, but not in those with a compromised immune system. This was associated with decreased STAT3 level and elevated immune stimulatory molecules. Pharmacologically inhibiting PI3Kβ also boosted anticancer immunity and when combined with immunotherapy, it slowed down tumor growth [228]. This study highlights the importance of PI3Kβ in immune evasion in breast tumor, particularly in the context of PTEN loss, and suggests that pharmacological inhibition of PI3Kβ could enhance anticancer immunity when combined with immunotherapy. Oncogenes facilitate the development of tumors in cells, yet their ability to suppress the immune system's response to cancer remains unclear. In a study, an in vivo expression screen of cancer‐related mutations in mouse tumor models treated with immune checkpoint blockade was performed. The identification of a particular mutation in PIK3CA, known as PIK3CA H1047R, was found to augment tumor growth when treated with immunotherapy through a decrease in CD^8+^ T cells and a rise in inhibitory myeloid cells. Nevertheless, blocking the entry of myeloid cells into these tumors resulted in the restoration of responsiveness to PD‐1 checkpoint blockade [38]. The identification of PIK3CA H1047R mutation's impact on immunotherapy responsiveness underscores the need to further investigate oncogene‐driven immune evasion mechanisms to improve cancer treatment outcomes.

PI3K/AKT shows significant promise in the regulation of immune evasion in human cancers. The immune checkpoint inhibitors such as PD‐L1 can be regulated by this axis. The upregulation of AKT increases PD‐L1 levels on tumor cells to suppress T cell activity and promote immune evasion. Moreover, PI3K/AKT axis can increase the recruitment of Treg cells and impair T cell activity through regulating cytokine levels. Upregulation of PI3K/AKT axis can promote the levels of immunosuppressive cytokines including TGF‐β and IL‐10 in providing immunosuppressive TME. Upregulation of PI3K/AKT can promote the recruitment of MDSCs to suppress T cell activity through arginase‐1 overexpression and nitric oxide generation. In addition, PI3K/AKT axis can suppress the maturation of dendritic cells and antigen‐presenting capacities to reduce immune responses. The expression of ligands for the function of NK cells is suppressed by PI3K/AKT axis reducing the activity of NK cells in the elimination of tumor cells. In addition, PI3K/AKT axis promotes the secretion of soluble form of these ligands for impairing the function of NK cells. The function of PI3K/AKT in Bcl‐2 and Mcl‐1 upregulation and apoptotic protein downregulation leads to resistance of cancer cells to function of immune cells such as T lymphocytes and NK cells. PI3K/AKT axis can enhance the M2 polarization of macrophages in promoting carcinogenesis and reducing anticancer immunity. In addition, this axis enhances VEGF expression to induce angiogenesis and disrupt the infiltration of immune cells to the TME due to the dysfunctional vasculature. The autophagy can be suppressed by PI3K/AKT to impair the immunogenic cell death and processes related to the release of DAMPs that are vital for the induction of DCs and T cell activities. More information on the function of PI3K/AKT axis in the regulation of immune system and cancer can be found in these reviews. More information regarding the function of PI3K/Akt/mTOR on cancer and immune system can be found in these reviews [229, 230, 231, 232, 233, 234].

The PI3K/AKT is a central regulator of immune evasion in cancer, promoting PD‐L1 expression, suppressing antigen presentation, and reshaping the TME to recruit immunosuppressive cells such as Tregs, MDSCs, and M2 macrophages. Modulators such as G3BP1 and oncogenic mutations including PIK3CA H1047R further enhance immune resistance by stabilizing PI3K activity or altering immune cell infiltration, while PTEN loss activates PI3Kβ‐driven immune suppression, especially in breast cancer. Targeting elements of the PI3K/AKT axis such as G3BP1, PI3Kβ, or mutant PIK3CA can restore antitumor immunity and improve responsiveness to immune checkpoint inhibitors, offering promising strategies for treating cancers.

The current section has provided significant discussion about the role of PI3K/AKT in the modulation of immune evasion in tumors and how it can affect the potential of ICIs in cancer therapy such as bladder and breast tumors. Such pathway has been beneficial in increasing the immune resistance through PDl‐L1 upregulation, suppressing antigen presentation (through MHC‐I downregulation) and altering TME to enhance and recruit immunosuppressive cells including Tregs, MDSCs, and M2 macrophages. The upregulation of PI3K/AKT significantly enhances the generation of immunosuppressive cytokines including TGF‐β and IL‐10 and accelerates the VEGF‐triggered angiogenesis. It also suppresses dendritic cell maturation and reduces the cytotoxic function of NK cells. There are some oncogenic mutations such as PIK3CA H1047R that can impair immune surveillance by decreasing CD^8+^ T cell induction and enhancing the invasion of suppressive myeloid cells. Inhibition of PI3K subtypes, such as PI3Kβ, or critical modulators such G3BP1, has demonstrated the capacity to accelerate antitumor immunity and improve responsiveness to PD‐1 inhibition in preclinical models. However, the tumor heterogeneity is one of the problems and limitations that making it difficult for targeting PI3K/AKT in cancer immunotherapy.

### 2.8 PI3K/AKT/mTOR in TME Remodeling

#### 2.8.1 Tumor‐Associated Macrophages

The upregulation of PD‐L1 expression on lung cancer cells has been shown to occur in response to IFN‐γ released by TAMs, primarily through activation of the JAK/STAT3 and PI3K/AKT pathways, while the MAPK/ERK pathway had no significant effect. Elevated PD‐L1 levels facilitated immune evasion by enhancing tumor cell invasiveness and suppressing antitumor immunity. Notably, inhibitors targeting JAK/STAT3 (AG490) and PI3K/AKT (LY294002) effectively reduced PD‐L1 expression, confirming the involvement of these cascades. These findings indicate a dual role of IFN‐γ, traditionally seen as antitumor, in promoting tumor progression under certain microenvironmental conditions [235]. These findings highlight the complex interplay between immune pathways and tumor progression, suggesting that targeting the JAK/STAT3 and PI3K/AKT pathways could be a promising strategy to reduce PD‐L1 expression and enhance antitumor immunity in lung cancer. Nasopharyngeal carcinoma (NPC) is a common head and neck malignancy in Southern China and Southeast Asia. Although the TME of NPC has been thoroughly understood, the significance of cell–cell interactions within the TME in facilitating carcinogenesis requires more investigation. C1q⁺ TAMs are significantly enriched in NPC, with both increased levels of C1q⁺ TAMs and higher C1q expression correlating with disease progression and worse prognosis. C1q has been shown to enhance malignancy and stemness by stimulating the PI3K/AKT via contact with GPR17, a G protein‐coupled receptor. This activation leads to the DNA hypermethylation inside tumor cells, therefore facilitating tumor progression and eliciting an immunosuppressive TAM phenotype. The development of NPC has been remarkably inhibited in a humanized mouse model via the specific inhibition of C1q by a neutralizing antibody. C1q⁺ TAM differentiation may furthermore transpire under conditions encouraging both M1 and M2 polarization [236]. Understanding the role of C1q⁺ TAMs in NPC progression underscores the importance of targeting the PI3K/AKT pathway to disrupt the immunosuppressive TME and improve therapeutic outcomes for NPC patients.

Liver metastasis is still one of the major causes of mortality in CRC because of the low response to therapy. The transcriptomic analysis of patient‐derived CRC and liver metastases highlighted SLITRK4 as the most significantly upregulated gene in metastatic lesions, associated with reduced overall survival. SLITRK4 has been demonstrated to accelerate CRC carcinogenesis, invasion, migration, and angiogenesis via the upregulation of the PI3K/AKT/NF‐κB pathway, alteration of the extracellular matrix, and upregulation of cytokine production. It can also facilitate the invasion and polarization of TAMs, with macrophage reduction significantly reducing SLITRK4‐mediated metastasis. The targeted suppression of SLITRK4 by lipid–polymer hybrid nanostructures for siRNA delivery significantly decreased liver metastasis, highlighting SLITRK4 as a viable therapeutic target for CRC [237]. The identification of SLITRK4 as a driver of CRC liver metastasis through the PI3K/AKT/NFκB pathway highlights its potential as a therapeutic target, emphasizing the need for further investigation into its role in tumor progression and immune modulation.

The GC cells demonstrate resistance to 5‐FU therapy, causing a significant challenge in its treatment and in this case, TAMs play a key role. Analysis of patient samples demonstrated that enhanced infiltration of CD68⁺ macrophages was correlated with a diminished response to treatment. The GC cells, especially those with resistance to 5‐FU therapy, can accelerate M2 polarization of macrophages, enhancing drug resistance. Such function is performed through release of CXCL5 by TAMs to induce PI3K/AKT/mTOR for enhancing recruitment of monocytes to enhance M2 polarization. Clinically, upregulated CXCL5 correlated with increased infiltration of CD163⁺ and CD206⁺ macrophages and reduced overall survival [199]. The correlation between CXCL5 expression and macrophage infiltration in GC suggests that targeting the CXCL5/PI3K/AKT/mTOR axis could enhance the efficacy of 5‐FU therapy by reducing M2 macrophage polarization and improving patient outcomes. IGFBP7 expression has been demonstrated to positively correlate with worse prognosis and increased macrophage infiltration in GC. IGFBP7 enhances GC proliferation and invasion, demonstrating its tumor‐promoting function. This protein is primarily generated in cancer‐associated fibroblasts (CAFs) and mesenchymal cells and its expression is modulated by EMT that can be upregulated by TGF‐β and downregulated by OVOL2 overexpression. FGFR1 expression is decreased during M1 macrophage polarization but its upregulation occurs in M2 polarization. Furthermore, administration of recombinant IGFBP7 in both macrophages and GC cells demonstrated that IGFBP7 promotes TAM polarization via the activation of the FGF2/FGFR1/PI3K/AKT [238]. The role of IGFBP7 in promoting TAM polarization via the FGF2/FGFR1/PI3K/AKT pathway underscores the importance of targeting this pathway to disrupt the immunosuppressive TME and improve therapeutic responses in GC.

#### 2.8.2 Cancer‐Associated Fibroblasts

A three‐dimensional (3D) microfluidic device was fabricated from polydimethylsiloxane to investigate the role of hepatocyte growth factor (HGF) derived by CAFs in the chemoresistance of A549 lung cancer cells. The system effectively replicated the TME, demonstrating that HGF from CAFs can stimulate Met/PI3K/AKT and enhance GRP78 expression in A549 cells, therefore reducing their susceptibility to paclitaxel‐induced apoptosis. Inhibition of HGF, Met, or PI3K abrogated these effects, but suppression of PI3K or GRP78 elevated apoptosis, hence reducing resistance mediated by the CAF matrix [239]. These findings highlight the critical role of HGF from CAFs in mediating chemoresistance in A549 lung cancer cells through the Met/PI3K/AKT pathway, suggesting that targeting this axis could enhance the efficacy of paclitaxel therapy. A compelling molecular mechanism underlying ESCC progression has been identified, where miR‐3656 was found to significantly enhance tumor cell proliferation, migration, and invasion. This miRNA, enriched in exosomes derived from CAFs, targets and downregulates ACAP2, a negative regulator of tumor growth. As a result, downstream PI3K/AKT and Wnt/β‐catenin pathways become activated, thereby accelerating tumor development both in vitro and in vivo. Notably, the tumor‐promoting effect of miR‐3656 was shown to be independent of its exosomal vehicle or cellular origin, indicating a broader oncogenic potential. These findings highlight a critical axis in ESCC progression and suggest miR‐3656 as a promising therapeutic target [240]. The identification of miR‐3656 as a key regulator of ESCC progression through the PI3K/AKT and Wnt/β‐catenin pathways underscores its potential as a therapeutic target, independent of its exosomal delivery mechanism. Another experiment has focused on understanding the function of CAF‐released exosomes in the regulation of OV progression. Gene expression analysis of CAF subpopulations revealed that secretory leukocyte protease inhibitor (SLPI) is significantly upregulated in FAP^high^ α‐SMA^low^ CAFs. SLPI is significantly expressed in CAFs, OC tissues, and EVs. SLPI released by CAF‐derived EVs promotes OC proliferation, migration, invasion, and adhesion by inducing PI3K/AKT. Clinically, increased plasma concentrations of EV‐encapsulated SLPI were associated with advanced tumor stage and decreased overall survival, highlighting SLPI as a reliable prognostic biomarker and therapeutic target in OC [241]. The role of SLPI in promoting OC progression via PI3K/AKT activation highlights its potential as a prognostic biomarker and therapeutic target, emphasizing the need for further investigation into its clinical applications.

The function of vitamin D receptor (VDR) in CAFs and their impact on chemotherapy resistance in GC was evaluated. It was demonstrated that VDR expression is lacking in CAFs and has negative association with disease severity and overall survival. Immunohistochemistry of GC tissues revealed that VDR is mainly located in gastric mucous cells, with downregulation correlated with advanced clinical stages. In a coculture system, the upregulation of VDR by calcipotriol significantly diminished CAF‐mediated OXA resistance in GC. RNA sequencing and cytokine research elucidated that IL‐8 released by CAFs can mediate resistance through the stimulation of the PI3K/AKT, which was significantly inhibited by VDR activation [215]. The study demonstrates that VDR activation in CAFs can overcome OXA resistance in GC by inhibiting IL‐8‐mediated PI3K/AKT signaling, suggesting a novel therapeutic strategy for improving chemotherapy outcomes.

Anal squamous cell carcinoma (ASCC) is a rare cancer and it has becoming prevalent. Currently, chemoradiotherapy is ineffective in around 20% of cases, with no targeted treatments for recurrence. An experiment has identified the PI3K/AKT/mTOR pathway with dysregulation in ASCC, with around 60% of patients demonstrating mutually exclusive genetic mutations in IGF2, IGF1R, PTEN, or PIK3CA. IGF2 that is mainly released by CAFs has been demonstrated to enhance proliferation via paracrine activation of the PI3K pathway. The findings highlight the essential function of the IGF2/PI3K and the role of CAFs in ASCC growth, highlighting that IGF2/IGF1R inhibitors are potential treatment options for this cancer [242]. The dysregulation of the PI3K/AKT/mTOR pathway in ASCC, driven by IGF2 from CAFs, highlights the potential of IGF2/IGF1R inhibitors as targeted treatments for this rare cancer. A pivotal role has been revealed for exosomal miR‐20a in promoting tumor progression and drug resistance. Elevated levels of miR‐20a have been identified within CAF‐derived exosomes, and their transfer to NSCLC cells has been shown to significantly enhance proliferation, inhibit apoptosis, and increase resistance to DDP. This oncogenic effect is mediated through the direct suppression of PTEN, leading to activation of the PI3K/AKT signaling pathway. Notably, PTEN overexpression reversed these malignant phenotypes, confirming its central role as a downstream target. Furthermore, in vivo studies validated that miR‐20a‐rich exosomes significantly promote tumor growth and chemoresistance, underscoring the therapeutic relevance of targeting this axis in NSCLC [217]. The oncogenic role of exosomal miR‐20a in promoting NSCLC progression and chemoresistance through PTEN suppression and PI3K/AKT activation highlights the therapeutic potential of targeting this pathway.

#### 2.8.3 T Cells and Tregs

CASZ1b is considered as an isoform of the TAL1‐regulated transcription factor and demonstrates upregulation in T‐cell acute lymphoblastic leukemia (T‐ALL) and accelerates disease development by inducing PI3K–AKT–mTOR. CASZ1b enhances T‐ALL cell viability, interacts with NOTCH1 in leukemogenesis, and affects chemotherapy resistance, emphasizing its potential as a therapeutic target [243]. The upregulation of CASZ1b in T‐ALL and its role in accelerating disease development through the PI3K/AKT/mTOR pathway highlight its potential as a therapeutic target, emphasizing the need for further investigation into its interactions with NOTCH1 and impact on chemotherapy resistance. Exosomal hsa‐miR‐23b‐3p is released from human bone marrow mesenchymal stem cells and suppresses the development of IA by targeting KLF5 and reducing PI3K/AKT/NF‐κB. Such approach has been beneficial in increasing the contractile phenotype of smooth muscle cells, decreases Th17 cell populations, and maintains the Th17/Treg equilibrium, therefore enhancing vascular remodeling and averting aneurysm formation [244]. The suppressive effect of exosomal hsa‐miR‐23b‐3p on IA development through the PI3K/AKT/NF‐κB pathway provides its therapeutic potential in enhancing vascular remodeling and maintaining immune balance, suggesting a novel approach to aneurysm prevention.

The pan‐Class I PI3K inhibitor copanlisib has demonstrated the capacity to elevate antitumor immune responses through the modulation of the TME. Intermittent administration of copanlisib led to increased infiltration of activated CD^8+^ T cells and macrophages, enhanced CD^8+^/Treg and M1/M2 macrophage ratios, and demonstrated significant antitumor activity, although the in vitro resistance of cancer cells to PI3K inhibition. In combination with anti‐PD‐1 ICIs, copanlisib enhanced treatment results, providing full remission and suppressing tumor recurrence in mice susceptible to ICIs [245]. The ability of copanlisib to modulate the TME and enhance antitumor immune responses, particularly when combined with anti‐PD‐1 ICIs, highlights its potential as a therapeutic strategy for improving treatment outcomes and suppressing tumor recurrence.

Graft‐versus‐host disease (GvHD) remains a significant challenge after allogeneic hematopoietic stem cell transplantation, even existing immunosuppressive treatments aimed against T cell alloreactivity. The PI3K/AKT/mTOR are essential for T cell activation and functionality, it has been recognized as a potential therapeutic target for the prevention of GvHD. The immunosuppressive impacts of PI3K inhibitors, have not been fully understood. The dual PI3K/mTOR inhibitor BEZ235 demonstrated significant suppression of T cell induction and proliferation at lower dosages compared with the selective PI3K inhibitor BKM120. BEZ235 also stimulated tolerance in alloreactive T cells, while preserving responses to CMV. Moreover, BEZ235 administration significantly diminished GvHD severity and increased survival in mouse models [246]. The dual PI3K/mTOR inhibitor BEZ235's efficacy in suppressing T cell induction and proliferation, and its potential to reduce GvHD severity while preserving antiviral responses, underscores its promise as a therapeutic target for preventing GvHD.

In chronic lymphocytic leukemia (CLL), persistent stimulation of B‐cell receptors, receptor tyrosine kinases (RTKs), and subsequent cascades leads to resistance to apoptosis and improved survival of leukemic cells. Idelalisib is a selective inhibitor of PI3K p110δ and has been considered for the treatment of relapsed or refractory CLL or instances with 17p deletions or TP53 mutations. Although it originally has demonstrated significant clinical responses, its therapeutic effectiveness has been restricted by the emergence of severe opportunistic infections. Idelalisib suppresses the activity of T and NK cells from both healthy donors and CLL patients. It specifically downregulated inhibitory checkpoint molecules, decreased T‐cell‐mediated cytotoxicity and granzyme B synthesis, suppressed cytokine secretion, and reduced NK cell proliferation and cytotoxic activity [247]. The selective PI3K p110δ inhibitor idelalisib's impact on T and NK cell activity, despite its clinical benefits, highlights the need to address its immunosuppressive effects and optimize its use in CLL treatment to mitigate the risk of opportunistic infections.

The integration of PI3K inhibition with ICB, especially PD‐1 blocking, has demonstrated to increase antitumor effectiveness in both preclinical and clinical conditions. High dosages of PI3K inhibitors compromise the functionality of both Tregs and CD^8+^ T cells; nevertheless, optimizing dosage and timing accelerates the targeted depletion of intratumoral Tregs. This selective inhibition leads to an increased population of tumor antigen‐specific CD^8+^ T lymphocytes. At the suitable dosage, PI3K is suppressed in both Tregs and activated CD^8+^ T cells; however, due to Tregs’ increased signaling reliance, this leads to their preferential suppression. As a result, the enhanced production of memory CD^8+^ T cells is obtained, facilitating sustained antitumor immunity [248]. The integration of PI3K inhibition with ICB, particularly PD‐1 blocking, demonstrates the potential to enhance antitumor immunity by selectively depleting intratumoral Tregs and increasing the population of tumor antigen‐specific CD^8+^ T cells, suggesting a promising strategy for optimizing cancer treatment.

#### 2.8.4 Hypoxia

AEG‐1 has been identified as a key contributor to hypoxia‐induced chemoresistance in HCC through modulation of the PI3K/AKT/HIF‐1α/MDR‐1 pathway. Elevated expression of AEG‐1 and MDR‐1 was observed in tumor tissues and further upregulated under hypoxic conditions. Knockdown of AEG‐1 significantly enhanced the cytotoxic and proapoptotic effects of adriamycin (ADM), 5‐FU, and DDP in hypoxic HCC cells. The suppression of AEG‐1 disrupted the PI3K/AKT cascade, reduced levels of HIF‐1α and MDR‐1, and upregulated PTEN, thereby sensitizing cells to chemotherapeutic agents [249]. The identification of AEG‐1 as a key regulator of hypoxia‐induced chemoresistance in HCC highlights its potential as a therapeutic target, emphasizing the need to disrupt the PI3K/AKT/HIF‐1α/MDR‐1 pathway to enhance chemotherapy efficacy. Radiosensitivity has been effectively enhanced through inhibition of the PI3K/mTOR pathway, leading to significant suppression of the HIF1‐α/VEGF‐A axis, which in turn augments the cytotoxic effects of IR. Reduced survival fractions and D10 values were observed, particularly in previously radioresistant cells, regardless of TP53 status. MEK inhibition showed minimal impact, indicating the specificity of the PI3K/mTOR pathway's role in radiosensitization. HIF1‐α knockdown further amplified IR‐induced cell death, highlighting its contribution to radioresistance [250]. The effective enhancement of radiosensitivity through PI3K/mTOR pathway inhibition and HIF1‐α knockdown highlights the potential of targeting these pathways to overcome radioresistance in cancer treatment.

Apigenin has been introduced as a potent chemotherapy drug in cancer therapy. In OC, a predominant cause of cancer‐related death in females, apigenin has been demonstrated to suppress the production of VEGF, as a factor involved in angiogenesis and proliferation. Such interaction correlates with decreased levels of HIF‐1α. Apigenin downregulates HIF‐1α and VEGF via inhibiting the PI3K/AKT/p70S6K1 and HDM2/p53. Furthermore, apigenin prevents tube formation by endothelial cells, demonstrating its antiangiogenic features [251]. The suppressive effect of apigenin on VEGF and HIF‐1α in OC highlights its potential as an antiangiogenic and antiproliferative agent, suggesting further investigation into its therapeutic applications in ovarian cancer.

One of the challenges in cancer therapy is the development of resistance due to the hypoxia. SM22α is an actin‐binding protein found in smooth muscle, fibroblasts, and certain epithelial cells and demonstrates upregulation in A549 NSCLC under hypoxic circumstances. The SM22α upregulation stimulates proliferation and increases resistance to chemotherapy and radiation through upregulating IGF1R/PI3K/AKT via direct interaction with IGF1Rβ. These results demonstrate SM22α as a prospective target for decreasing therapeutic resistance caused by hypoxia [252]. The role of SM22α in promoting hypoxia‐induced resistance in NSCLC through the IGF1R/PI3K/AKT pathway highlights its potential as a target to reduce therapeutic resistance in lung cancer.

A hypoxic TME facilitates the GC progression, especially via hypoxia‐induced autophagy as a survival strategy. Isorhamnetin (ISO) is a phytochemical extracted from sea buckthorn and has demonstrated antitumor features; nevertheless, its effect under hypoxic condition in stomach cancer remains challenging. ISO suppresses hypoxia‐induced autophagy by downregulating PI3K and suppressing the PI3K/AKT/mTOR. Consequently, ISO suppresses growth, diminishes mitochondrial membrane potential, and stimulates mitochondria‐mediated death [253]. The ability of isorhamnetin to suppress hypoxia‐induced autophagy and tumor growth in GC through PI3K/AKT/mTOR inhibition displays its potential as a therapeutic agent in hypoxic tumor environments.

Microcalcifications in invasive breast cancer are associated with increased tumor proliferation, migration, and resistance to doxorubicin, providing a more aggressive character. Therefore, PI3K/AKT pathway facilitates calcification and enhances HIF1α expression, hence supporting tumor proliferation and medication resistance. Inhibition of HIF1α diminishes proliferation and migration, while causing doxorubicin sensitivity despite persistent calcification [254]. In addition to this, highlighting the association between hypoxia and cancer progression (metastasis) in terms of PI3K/AKT regulation is of importance. Tumor hypoxia enhances cancer aggressiveness and adversely affects prognosis via HIF‐1α‐mediated signaling enhancing angiogenesis, EMT, and metastasis. The cinnamaldehyde (CA) is a principal component derived from *Cinnamon cassia* bark and significantly suppresses hypoxia‐induced angiogenesis and metastasis in sarcoma 180 and B16F10 melanoma models. CA decreases VEGF production, FLK phosphorylation, MMP activity, and EMT markers (TWIST and ZEB1). Mechanistically, CA diminishes HIF‐1α protein levels by suppressing its synthesis through the suppression of the PI3K/AKT/mTOR pathway, highlighting its potential as a hypoxia‐targeting anticancer drug [255]. The suppressive effect of CA on hypoxia‐induced angiogenesis and metastasis through the PI3K/AKT/mTOR pathway underscores its potential as a hypoxia‐targeting anticancer drug.

Hypoxia‐mediated pharmacological resistance provides a significant challenge in cancer therapy. Wogonin significantly suppresses hypoxia‐induced resistance in HCT116 colon cancer, remarkably enhancing sensitivity to paclitaxel, ADM, and DDP. Wogonin performs this by downregulating HIF‐1α and essential glycolysis‐related proteins (HKII, PDHK1, LDHA), thereby reducing glucose absorption and lactate synthesis. The reversal effect is associated with the suppression of the PI3K/AKT. Wogonin suppresses tumor development and the generation of HIF‐1α, glycolytic enzymes, and PI3K/AKT, highlighting its potential as an effective treatment against hypoxia‐induced chemoresistance [256].

Tumor hypoxia is a feature of solid tumors and it can accelerate tumorigenesis, EMT, and therapeutic resistance, causing poor prognosis. The HIF‐1α is the key regulator of hypoxia and it can control the expression of genes related to the angiogenesis, glycolysis, metastasis, and resistance to treatment. Several studies have recognized the PI3K/AKT/mTOR as a vital upstream regulator of HIF‐1α, providing it as a significant therapeutic target. In GC, HIF‐1α expression demonstrates upregulation in serum and tumor tissues, associating with enhanced proliferation, migration, and invasion via PI3K/AKT‐triggered VEGF production. Natural compounds such as wogonin, CA, Huaier, and isorhamnetin have demonstrated efficacy in overcoming hypoxia‐induced drug resistance or suppressing EMT and carcinogenesis by affecting PI3K/AKT/HIF‐1α, consequently reducing glycolysis, angiogenesis, and metastatic potential in cancers. The dual PI3K/mTOR inhibitor NVP‐BEZ235 has demonstrated potential in overcoming hypoxia‐ and TGF‐β1‐induced EMT in OC and PCa by downregulating HIF‐1α and its related molecules, including Snail and phosphorylated Smad2/3, resulting in the reinstatement of E‐cadherin and a decrease in cellular motility. In CRC, the nuclear receptor Nur77 has been recognized as a novel promoter of hypoxia‐induced metastasis by controlling miRNA biogenesis through the suppression of Dicer via p63 suppression, which subsequently increases PI3K subunit p110α stability and stimulates AKT/β‐catenin, thereby facilitating EMT and cancer stemness [257, 258, 259, 260]. The recognition of the PI3K/AKT/mTOR pathway as a key regulator of hypoxia‐induced EMT and drug resistance in various cancers underscores the importance of targeting this pathway to improve treatment outcomes and overcome therapeutic challenges.

In breast cancer, hypoxia‐induced HIF‐1α upregulation is accelerated by a cascade including ROS, PI3K/ERK upregulation, and Rac1 signaling, causing upregulation of VEGF and enhanced tumor aggressiveness. In CRC, HIF‐1α is vital in accelerating resistance to 5‐FU by upregulating glucose metabolic reprogramming, enhancing glycolysis and activating pentose phosphate pathway. The elevation of HIF‐1α in 5‐FU‐resistant CRC cells is performed via nonhypoxic pathways involving ROS‐mediated induction of PI3K/AKT and nuclear β‐catenin. The suppression or knockdown of HIF‐1α improves drug sensitivity and highlights its potential as a predictive biomarker for 5‐FU response. In OC, hypoxia has been demonstrated to stimulate the production of miR‐9‐5p, accelerating migration, invasion, and tumor proliferation through the PI3K/AKT/mTOR/GSK3β, thereby recognizing miR‐9 as a prospective oncogenic driver and therapeutic target [201, 261, 262].

The present section highlighted the complicated role of PI3K/AKT in the regulation of immune evasion, stromal interaction, and hypoxia‐mediated mechanisms within the TME, therefore highlighting its importance as therapeutic factor in tumor cells. The PI3K/AKT pathway not only accelerates proliferation and survival but also enables immune evasion by upregulating PD‐L1, suppressing antigen presentation, and providing immunosuppressive TME elements such as M2 macrophages, Tregs, and MDSCs. The oncogenic mutations (PIK3CA H1047R) and the loss of tumor suppressors (PTEN) enhance immune resistance via this pathway, whereas pharmacological inhibition (PI3Kβ) enhances immune responses and improves the efficiency of immunotherapy. Furthermore, PI3K/AKT enhances the carcinogenic function of TAMs and CAFs. Cytokines produced from TAMs (CXCL5) and substances released by CAFs (SLPI, IGF2, IL‐8) stimulate the PI3K/AKT pathway, accelerating carcinogenesis, metastasis, angiogenesis, and chemoresistance in several tumors, including GC, OC, and CRC. Hypoxia promotes these impacts, since PI3K/AKT‐induced stabilization of HIF‐1α facilities angiogenesis, EMT, glycolysis, and therapeutic resistance. Natural substances (wogonin, isorhamnetin, CA) and specific inhibitors (NVP‐BEZ235) have highlighted the potential in providing these effects by suppressing PI3K/AKT/HIF‐1α. The dual function of PI3K/AKT in regulating immune cell activity, such as T cells and NK cells, provides a therapeutic challenge, requiring highlighting and understanding dosing and timing strategies to enhance CD^8⁺^ T cell efficacy, while reducing inhibitory Tregs. A significant limitation is the complexity of the TME and the context‐dependent impacts of PI3K/AKT in various cancers. The subsequent research should focus on the development, application, and combination of PI3K inhibitors with immunotherapeutic agents, the recognition of predictive biomarkers for the therapeutic response, and the evaluation of how such regulation of this pathway can affect the complicated interactions among tumor cells, stromal factors, and immunological elements.

### 2.9 PI3K/AKT/mTOR Regulators in Cancer

#### 2.9.1 Small Molecules

Small molecules with significant anticancer activity demonstrate promise for enhancing treatment efficacy and minimizing side effects. In an effort, new composites as PI3K/AKT inhibitors have been synthesized in which W934 is the most potent one. The anticancer function of W934 is related to the downregulation of PI3Kα kinase and significant reduction in viability, with IC_50_ values of 0.25 and 0.23 µmol/L. Moreover, W934 suppressed migration, and promoted apoptosis. It also disrupted G0–G1 phase progression and prevented the PI3K/AKT axis [263]. The potent anticancer activity of W934, particularly its ability to downregulate PI3Kα kinase and inhibit the PI3K/AKT axis, highlights its potential as a therapeutic agent for enhancing treatment efficacy and minimizing side effects. A promising therapeutic strategy has been demonstrated with significant efficacy and reduced toxicity. By utilizing a nanoprodrug with high drug loading, codelivery of HSP and TPL has been achieved, enabling dual inhibition of EGFR and the PI3K/AKT pathway. Enhanced cellular uptake, targeted delivery via EGFR binding, and acid‐triggered drug release have been confirmed. In vitro, tumor cell proliferation was suppressed and apoptosis was induced, while in vivo, substantial tumor growth inhibition was observed without marked systemic toxicity. Notably, improved pharmacokinetics and biosafety profiles were also achieved, supporting the therapeutic potential of this approach for BCa [264]. The successful codelivery of HSP and TPL via a nanoprodrug, achieving dual inhibition of EGFR and the PI3K/AKT pathway, highlights a promising therapeutic strategy for BCa with enhanced efficacy and reduced toxicity.

SCH‐527123, a small‐molecule antagonist of CXCR1 and CXCR2, was found to significantly inhibit proliferation, migration, and invasion of human melanoma cell lines A375 and M14, while promoting apoptosis. These effects were associated with the downregulation of CXCR1 and CXCR2 expression and the suppression of the PI3K/AKT. The findings suggest that SCH‐527123 may be a potential therapeutic agent for melanoma by targeting chemokine receptors and interfering with key molecular pathways involved in tumor progression [265]. The significant inhibition of proliferation, migration, and invasion of melanoma cells by SCH‐527123 through the downregulation of CXCR1 and CXCR2 and suppression of the PI3K/AKT pathway suggests its potential as a therapeutic agent for melanoma. Since melanoma is a skin cancer and chemokines participate in its progression, utilizing previous strategy in the modulation of CXCR1/CXCR A study was developed to design a novel PSMA–PI3K small patch drug conjugate known through affecting Pi3K/AKT can further enhance its treatment.

A study was developed to design a novel PSMA–PI3K small patch drug conjugate known as conjugate 1. It disrupts PI3K with an IC_50_ of 0.40 nM, specifically emphasizing on PSMA and significantly reducing growth in PSMA‐positive cancer cells. Furthermore, conjugate 1 decreases phosphorylation of vital PI3K downstream effectors, causes the cell cycle arrest in the G0/G1 phase, and demonstrates favorable efficacy and tolerability in vivo [266]. In addition to this small molecule, another compound has been developed to affect PI3K subunits. The pharmacologically active vasicinone analogue, RLX, possesses significant anticancer effects. RLX effectively inhibits proliferation in colon tumor. The medium involves downregulation of PI3K subunits p110α and p85, resulting in the reduced downstream protein expression. Besides, RLX promotessub‐G1 arrest and changes mitochondrial function, attesting its antiproliferative action via the PI3K pathway [267]. The anticancer effects of RLX, particularly its ability to inhibit proliferation through the downregulation of PI3K subunits and induction of cell cycle arrest, underscore its potential as a therapeutic agent for colon cancer.

Regarding the potential of PI3K in the autophagy regulation, the application of small molecules targeting this pathway can affect autophagy mechanism in human cancers. A011 has been introduced as a potent anticancer compound for the breast tumor therapy and it is a ligand of σ_2_ receptor. A011 increases the calcium levels in the cells and facilitates ROS generation. In addition, A011 mediates autophagy and it can stimulate ER stress. A011 stimulates PERK–eIF2α–CHOP axis, while it impairs PI3K/AKT/mTOR, leading to the apoptosis [268]. The role of A011 in mediating autophagy and apoptosis through the disruption of the PI3K/AKT/mTOR pathway highlights its potential as a therapeutic agent for breast cancer, emphasizing the importance of targeting autophagy mechanisms. A novel and potent mechanism for selective anticancer activity has been demonstrated through the inhibition of mTOR/AKT signaling via disruption of trans‐Golgi‐network trafficking. Significant suppression of AKT phosphorylation was achieved indirectly, without direct enzymatic inhibition, by inducing lipid raft disorganization, displacing mTOR from its functional complex, and triggering ER stress. Schweinfurthin compounds showed enhanced toxicity toward PTEN‐deficient cancer cells, particularly in DLBCL, while sparing normal PBMCs and fibroblasts. The trafficking blockade was mechanistically linked to the interaction of schweinfurthins with oxysterol‐binding proteins, suggesting a unique dependency on vesicular lipid transport for mTOR/AKT pathway maintenance [269].

Targeting ChoKα with a novel competitive inhibitor has been shown to significantly disrupt key oncogenic pathways and suppress tumor progression. Through selective inhibition of ChoKα activity, intracellular levels of PCho, PC, and PA were markedly reduced, leading to attenuation of MAPK and PI3K/AKT signaling. This biochemical disruption impaired cytoskeletal organization, diminished membrane ruffling, and selectively inhibited proliferation and anchorage‐independent growth in cancer cells without affecting normal cells. In vivo, tumor growth was substantially reduced alongside decreased phosphorylation of ERK and AKT, confirming the therapeutic potential of small molecule ChoKα antagonists in Ras‐driven malignancies [270]. The significant disruption of oncogenic pathways and suppression of tumor progression through selective inhibition of ChoKα activity highlights the therapeutic potential of small molecule ChoKα antagonists in Ras‐driven malignancies. The clinical studies have also evaluated the application of small molecules for cancer therapy. PF‐05212384 (PKI‐587) is a small molecule capable of dual inhibition of PI3K and mTOR. This small molecule has been administered intravenously (154 mg). The patients responded to the treatment and a number of patients demonstrated side effects such as inflammation, stomatitis, nausea, hyperglycemia, fatigue, and vomiting, among others [271].

In spite of the promises by small molecules in cancer therapy, there are a number of challenges to be considered. First of all, a variety of cancers demonstrate intrinsic resistance to small molecules because of the tumor heterogeneity or induction of survival mechanisms. Therefore, the combination of small molecules with genetic tools can empower their potential in tumor suppression. In addition, the frequent application of small molecules can result in the induction of adaptive resistance mechanisms. There are a number of side effects for these small molecules, as PI3K/AKT/mTOR axis participates in the regulation of normal mechanisms including metabolism and immune regulation. Therefore, the suppression of PI3K/AKT/mTOR axis can cause side effects such as hyperglycemia, immune suppression, and gastrointestinal issues. The poor selectivity is another problem of small molecules and these compounds lack specificity and in addition to the cancer cells, they can affect normal cells. Therefore, it is suggested to utilize nanostructures for the delivery of small molecules in cancer therapy and suppression of PI3K/AKT/mTOR axis. The nanoparticles can also deliver small molecules with other compounds and drugs in cancer therapy. There are challenges regarding the responsive patient subgroups and application of biomarkers for predicting therapeutic efficacy resulting from inter‐ and intratumor heterogeneity. The downregulation of PI3K/AKT/mTOR axis can further stimulate complementary mechanisms for reducing therapeutic efficacy of small molecules. The small molecules suffer from poor solubility, rapid metabolism, or inadequate tumor penetration, restricting their clinical application. Another issue is that TME has mild acidic pH and it should be highlighted that this pH can provide any impact on the chemical structure and function of small molecules. In this case, encapsulation in other carriers can provide protection against such stimuli.

#### 2.9.2 Phytochemicals

In addition to small molecules as PI3K/AKT/mTOR regulator, the naturally occurring compounds have been emerged as other modulators of this pathway. Promising therapeutic effects have been demonstrated through targeted inhibition of the PI3K/AKT. Significant reductions in GC cell viability, migration, and invasion were observed following treatment with the natural flavonoid astragalin (AST). Tumor growth was suppressed in vivo without evident toxicity, and a dose‐dependent decrease in tumor volume and weight was recorded. Induction of apoptosis was confirmed via increased PARP cleavage and modulation of Bcl‐2 and Bim protein levels. Furthermore, phosphorylation of PI3K and AKT was attenuated, while EGF‐stimulated activation of this pathway diminished AST's inhibitory effects, highlighting pathway specificity [272]. More importantly, the phytochemicals demonstrate pleiotropic functions and they can affect several pathways and molecular factors. Significant anticancer activity has been demonstrated by rosmanol, a phenolic diterpene, through its ability to selectively inhibit the proliferation of MCF‐7 and MDA‐MB‐231 breast cancer cells without affecting normal MCF‐10A cells. Apoptosis has been induced via mitochondrial pathways, involving increased ROS production, DNA damage, and activation of caspase‐8, ‐9, and ‐3, with the process attenuated by NAC and Z‐VAD–FMK. Cell cycle arrest has been observed in the S phase, accompanied by upregulation of cyclin A and downregulation of cyclins B1, D1, and E. Furthermore, ERK and PI3K/AKT pathways have been suppressed, while p‐p38 expression has been elevated. The JAK2/STAT3 axis has been effectively inhibited by reducing phosphorylation levels and upregulating PIAS3, contributing to the proapoptotic and antiproliferative effects of rosmanol [273].

Flavokawain C accumulates preferentially in liver and significantly inhibits cancer proliferation and migration, induces apoptosis, and causes DNA damage. Flavokawain C suppresses the FAK/PI3K/AKT axis by binding to ATP on FAK and PI3K, therefore inhibiting their phosphorylation [274]. In addition to apoptosis and DNA damage, they can affect cell cycle arrest. Therefore, another experiment was performed to follow this step. Voacamine (VOA) remarkably inhibited the proliferation of MCF‐7 and 4T1 bone cancer cells with IC_50_ values of 0.99 and 1.48 µM. VOA also halted migration and colony conformation, arrested the cell cycle in S phase, and facilitated natural apoptosis while suppressing the PI3K/AKT/mTOR axis [275].

A promising strategy has been identified for overcoming DDP resistance in NSCLC. Significant growth inhibition, G0/G1 cell cycle arrest, and apoptosis were induced in lung cancer cells following exposure to halofuginone (HF). A synergistic effect with DDP was demonstrated, with notable reductions in IC_50_ values when combined treatment was applied. Downregulation of the PI3K/AKT and MAPK was confirmed at both transcriptomic and protein levels, indicating that dual‐pathway suppression played a crucial role in sensitizing resistant cells. These findings suggest that enhanced therapeutic responses may be achieved by targeting key resistance‐associated pathways using natural compounds [276]. HF's ability to overcome DDP resistance in NSCLC through dual suppression of the PI3K/AKT and MAPK pathways highlights its potential as a synergistic therapeutic agent for enhancing treatment efficacy.

Echinacoside (ECH) can affect a number of factors including PI3K, AKT, HIF‐1, and VEGF in cancer therapy. PIK3R1 and PIK3CD as crucial targets were considered. ECH's inhibitory effect on MCF‐7 cell proliferation and its capability to induce apoptosis by downregulating crucial proteins, while in vivo trials showed significant growth reduction, indicating that ECH influences BC through the PI3K/AKT/HIF‐1α/VEGF pathway [277]. In addition to apoptosis, the phytochemicals are able to regulate angiogenesis and other cancer hallmarks. A promising therapeutic effect was observed with EC, as cell proliferation, migration, invasion, and angiogenesis were significantly reduced, while apoptosis was significantly enhanced in a dose‐dependent manner. These outcomes were achieved through the suppression of the PI3K/AKT/mTOR pathway, as evidenced by the decreased phosphorylation levels of PI3K, AKT, and mTOR. The inhibition effects were partially reversed upon activation of PI3K, confirming pathway involvement. Furthermore, tumor growth was effectively attenuated in vivo without adverse impacts on body weight, aligning with reduced expression of VEGFA and other malignancy‐associated markers. Overall, strong antitumor potential was demonstrated for EC via molecular and phenotypic suppression of OC progression [278]. More importantly, since the PI3K axis is related to autophagy mechanism, the phytochemicals are also able to indirectly affect autophagy. Chaetocochin J (CJ), an ETP alkaloid from *Chaetomium sp*., was insulated, and its anti‐CRC activity was examined. CJ demonstrated significant proliferation inhibition, with an IC_50_ of roughly 0.5 µM, and facilitated apoptosis. CJ induced autophagic flux without affecting its apoptotic effect when autophagy was inhibited, acting through the AMPK upregulation and PI3K/AKT/mTOR downregulation [279].

A potent anticancer effect was demonstrated through a dual apoptotic mechanism following treatment with lupiwighteone. Significant inhibition of cell proliferation was observed in both ER‐positive and triple‐negative human breast cancer cells, along with classical apoptotic features such as DNA fragmentation and nuclear condensation. Caspase‐dependent apoptosis was induced, as evidenced by increased expression of caspase‐3, ‐7, ‐8, ‐9, Bax, and cleaved‐PARP, and reduced levels of Bid and Bcl‐2. Concurrently, caspase‐independent apoptosis was triggered through the upregulation of cytosolic AIF and Endo G. These apoptotic events were associated with inhibition of the PI3K/AKT/mTOR, as lupiwighteone treatment led to downregulation of PI3K, p‐AKT, and p‐mTOR, indicating suppression of survival and proliferative signaling [280].

There are a number of challenges with phytochemicals including poor solubility, rapid metabolism, and short half‐life. The phytochemicals demonstrate poor water solubility that decreases their absorption and bioavailability. The natural compounds show significant first‐pass metabolism in the liver tissue and gastrointestinal tract. In addition, restricted systemic circulation reduces the therapeutic index of phytochemicals. The function of phytochemicals is not certain and in addition to PI3K/AKT axis, they can affect other molecular pathways, highlighting their poor specificity, but pleiotropic impacts. Due to the differences in potential, determining an effective and safe dose for the phytochemicals is challenging. The application of high levels of phytochemicals may cause cytotoxicity and a number of adverse impacts. There is inconsistent absorption and poor tissue penetration. The mechanisms by which phytochemicals affect PI3K/AKT/mTOR axis have not been fully understood. Moreover, cancer cells may induce survival pathways to develop resistance to anticancer activity of phytochemicals. Since both small molecules and phytochemicals suffer from poor pharmacokinetic profile, the next section will focus on the delivery of these agents with nanoparticles in effective cancer therapy. In order to improve the regulation on PI3K/AKT/mTOR axis, the studies can focus on the combination of phytochemicals and small molecules in cancer therapy.

#### 2.9.3 Nanoparticles

In the recent years, nanoparticles have been significantly deployed in the different parts of oncology. The interest toward nanoparticles results from their potential in targeted drug and gene delivery, and accelerating chemotherapy, radiotherapy, and immunotherapy. There are several challenges with the traditional therapies in which resistance and poor tumor accumulation are considered to be the most prominent ones. In case of cancer therapy, the application of nanoparticles can significantly promote the accumulation of drugs at the tumor site. Therefore, nanostructures have been introduced for the targeted cancer therapy and this section focuses on the specific regulation of PI3K/AKT/mTOR axis.

An experiment has designed isoliquiritigenin‐zein phosphatidylcholine nanostructures for breast cancer therapy. These nanostructures demonstrated prolonged organ retention and significant antitumor efficacy suppressing growth and metastasis by downregulating PI3K/AKT/mTOR and MMP2/9 [281]. The function of this nanocarrier is beyond the regulation of mTOR and it is able to selectively deliver the drug for boosting anticancer function. Therefore, this activity can be related to the improved biomedical applications of nanoparticles. A promising therapeutic potential has been demonstrated through the use of 0.8% Ni‐doped CFO nanoparticles, which significantly inhibited the proliferation and migration of MCF‐7 cells while enhancing intracellular ROS production. Apoptotic induction was evidenced by increased LPO, decreased antioxidant enzyme levels (SOD, CAT, GPx), and upregulated expression of proapoptotic markers (p53, Bax, cleaved caspases 3/8/9), accompanied by the downregulation of Bcl‐2. Notably, suppression of p‐PI3K, p‐AKT, and p‐mTOR confirmed the inhibition of the PI3K/AKT/mTOR. Furthermore, potent antibacterial activity was observed against multiple pathogens, highlighting the dual functional efficacy of these nanoparticles in biomedical applications [282].

The remarkable cytotoxic effect was demonstrated by Si‐SeNPs against GC cells through selective induction of apoptosis and autophagy without affecting normal cells. Elevated expression of Bax, Cyt *c*, and cleaved caspases alongside suppression of Bcl‐2 indicated activation of mitochondrial apoptosis. Concurrently, autophagy flux was triggered, as shown by increased LC3‐II and Beclin1 and decreased p62 levels. This dual effect was further intensified by downregulation of the PI3K/AKT/mTOR pathway, confirmed by reduced phosphorylation of key pathway proteins. Moreover, inhibition of this pathway was shown to mediate the cross‐talk between autophagy and apoptosis, enhancing cancer cell death [283]. Along with this, such impacts have been also evaluated on CC. Another research highlights the biosynthesis of bimetallic silver/zinc oxide nanocomposites (Ag@ZnO NCs) utilizing Crocus sativus extract and evaluates their anticancer efficacy against cervical tumor. The biosynthesized Ag@ZnO NCs demonstrated considerable stability, demonstrating a median particle size of 80–90 nm and a zeta potential of −14.70 mV. The EDX analysis confirmed the existence of AgNPs and ZnO. A 5 µg/mL treatment produced a 58 ± 2.9% inhibitory effect on HeLa cells after 24 h, triggering apoptosis and the production of ROS, while also suppressing the PI3K, AKT, and mTOR [284].

In spite of the promising results of nanoparticles in cancer therapy, addressing some challenges can improve the chance in cancer therapy. The long‐term stability of nanostructures during storage and within biological system should be ensured. Moreover, nanoparticles should demonstrate high drug loading and encapsulation efficiency for the suppression of PI3K/AKT/mTOR axis in cancer therapy. The large‐scale production of nanoparticles with consistent quality should be also considered. There is risk of toxicity on the healthy tissues because of the nonspecific distribution of nanostructures. In addition, the penetration of nanoparticles to the TME can be decreased by the dense extracellular matrix and high interstitial fluid pressure. This is also prominent in some of the tumors including pancreatic cancer possessing dense TME. The function of nanoparticles may be affected by the hypoxia and acidic pH. There is also inconsistent delivery due to the variations in EPR effect in various cancers. The biocompatibility and biosafety of the nanoparticles for the long‐term should be considered. Therefore, the synthesis of nanoparticles from green sources is suggested. The pre‐mature drug release before reaching to the tumor site and the development of stimuli‐responsive nanostructures can improve the efficacy in the regulation of PI3K/AKT axis in cancer therapy. More focus should be directed to the development of biomimetic nanoparticles capable of immune evasion and reaching to the tumor site. The biomimetic nanoparticles decrease the clearance by the reticuloendothelial system and increase penetration through cell membrane. One of the new advances in understanding the function of nanoparticles and their circulation in bloodstream is related to the formation of protein corona around the nanoparticles, affecting their accumulation and reaching to the tumor cells.

### 2.10 Association of PI3K/AKT/mTOR With Core Pathways and Biological Mechanisms

The present review provided a comprehensive discussion of the function of PI3K/AKT/mTOR axis in cancer. This pathway exerts an oncogenic function and its abnormal expression decreases survival of patients. The core pathways and mechanisms regulated by this pathway can be summarized as follows:

The PI3K/AKT/mTOR signaling axis is a central driver of oncogenesis, fundamentally intertwined with the core pathways and biological mechanisms defining cancer. This hyperactivated pathway, frequently triggered by gain‐of‐function mutations in PIK3CA (encoding the p110α catalytic subunit of PI3K), loss‐of‐function mutations or deletions in the tumor suppressor PTEN (which dephosphorylates PIP3), or constitutive activation of upstream RTKs, serves as a critical signaling hub. Its activation converges diverse extracellular growth and survival signals, including insulin, IGF‐1, and EGF, into potent intracellular responses. The axis functions by phosphorylating and modulating a vast array of downstream substrates. PI3K‐generated PIP3 recruits AKT to the membrane, where it's activated by PDK1 and mTORC2 phosphorylation. Activated AKT then phosphorylates numerous targets, crucially including the activation of mTORC1, the master regulator of anabolic processes. This positions the PI3K/AKT/mTOR axis as a linchpin integrating external cues with the internal machinery controlling cell fate and metabolism, making it indispensable for many cancer hallmarks.

This axis exerts profound control over core cancer biological mechanisms, including uncontrolled proliferation, evasion of cell death, and altered metabolism. AKT directly phosphorylates and inhibits key proapoptotic proteins, such as BAD and procaspase‐9, while promoting antiapoptotic signals via NF‐κB activation, thereby conferring robust resistance to cell death. Simultaneously, AKT drives cell cycle progression by phosphorylating and inhibiting negative regulators, including GSK‐3β (stabilizing cyclin D1) and the FoxO transcription factors. Furthermore, the axis is a master regulator of cancer metabolism. AKT promotes glucose uptake (via GLUT transporters) and glycolytic flux (via regulation of HK2 and PFKFB), facilitating the Warburg effect. mTORC1, activated by AKT and nutrients, is the primary driver of protein synthesis through phosphorylation of 4E‐BP1 and S6K1, and stimulates lipid and nucleotide biosynthesis, providing the essential building blocks for rapid tumor growth. This orchestration of anabolic processes fuels uncontrolled proliferation.

Critically, the PI3K/AKT/mTOR axis exhibits extensive crosstalk and integration with other major oncogenic pathways, forming a complex signaling network. It has well‐documented bidirectional interactions with the RAS/MAPK pathway; RAS can activate PI3K, while downstream effectors such as S6K1 (an mTORC1 target) can provide negative feedback to RTKs and IRS proteins, dampening PI3K activation. It antagonizes the p53 tumor suppressor pathway; AKT phosphorylates MDM2, promoting p53 degradation, and mTORC1 can inhibit p53 activation under stress. Conversely, p53 can induce PTEN and inhibit the PI3K pathway. The axis also intersects with pathways controlling angiogenesis (via HIF‐1α stabilization by mTORC1), invasion and metastasis (via regulation of EMT transcription factors including Snail and MMP expression), and the DDR. AKT phosphorylates CHK1, influencing cell cycle arrest after damage, and can modulate BRCA1 function. This intricate web means PI3K/AKT/mTOR dysfunction amplifies oncogenic signals across multiple pathways and undermines tumor suppressive mechanisms.

The centrality of the PI3K/AKT/mTOR axis makes it a prime therapeutic target, but its complex regulation and feedback loops also drive therapeutic resistance. Inhibition of mTORC1 (with rapalogs) often relieves feedback inhibition of AKT (via S6K1–IRS1), leading to paradoxical AKT activation and limited efficacy. Similarly, AKT inhibitors can relieve feedback inhibition on RTKs, causing pathway reactivation. Combining inhibitors targeting different nodes of the axis (PI3K and mTOR) or combining axis inhibitors with agents targeting compensatory pathways (MEK inhibitors, ERK inhibitors, antiangiogenics) or DNA repair mechanisms (PARP inhibitors, especially in tumors with PTEN loss or PIK3CA mutations showing homologous recombination deficiency phenotypes) represents key strategies. Furthermore, the axis profoundly influences the TME, promoting immunosuppression by inhibiting T‐cell function and promoting Treg activity, highlighting its role in immune evasion. Targeting specific PI3K isoforms, such as PI3Kδ/γ, shows promise in modulating immune responses. Understanding the context‐specific wiring of this axis within a tumor's unique genetic and epigenetic landscape, including its integration with other core pathways such as RAS/MAPK, p53, and DDR, remains crucial for developing effective, personalized therapeutic strategies to overcome the inherent resilience of cancer cells driven by this master regulator.

### 2.11 Clinical Studies of PI3K/AKT/mTOR Inhibitors

The PI3K/AKT/mTOR has become an essential therapeutic target in several solid cancers because of its pivotal function in tumor proliferation, survival, and resistance to therapy. Although the function of this pathway in hematological cancers has been ignored, this pathway is also critical for the regulation of these tumors. A variety of early‐phase clinical trials have investigated various PI3K inhibitors, both as treatments and in conjunction with other drugs in various tumors, highlighting the potential and difficulties of targeting this pathway. Buparlisib, a pan‐PI3K inhibitor, has demonstrated desirable tolerability at a daily dosage of 100 mg when administered with cetuximab in recurrent/metastatic HNSCC, showing signs of disease stabilization even in patients previously treated with cetuximab. In the BASALT‐1 trial, buparlisib monotherapy possessed limited effectiveness in PI3K‐activated NSCLC, demonstrating the requirement for the combination treatments. Taselisib, a beta‐sparing PI3K inhibitor, had favorable tolerability when combined with tamoxifen in ER‐positive metastatic breast cancer and highlighted early efficacy, with disease control being significant in both PIK3CA mutant and wild‐type tumors. Gedatolisib, a dual PI3K/mTOR inhibitor, combined with irinotecan or the MEK inhibitor PD‐0325901, had moderate therapeutic benefits in patients with advanced solid tumors; however, frequent dosage delays impeded prolonged exposure. Clinical activity was demonstrated in certain subgroups of individuals with ovarian and endometrial cancers possessing KRAS mutations. Another PI3K/mTOR inhibitor, PF‐05212384, demonstrated a desirable safety profile with promising results of clinical efficacy, including partial responses and improved disease stability in a pretreated population, thereby reinforcing the need for more assessment of PI3K pathway‐targeted therapies [271, 285, 286, 287, 288].

A phase I study evaluated the safety and efficacy of mTOR inhibitors (temsirolimus or everolimus) in combination with liposomal doxorubicin and bevacizumab (DAT or DAE regimens) in 52 females diagnosed with advanced metaplastic TNBC, a histological surrogate for mesenchymal TNBC, demonstrating PI3K pathway abnormalities. Such combination treatment demonstrated an objective response rate of 21% and a clinical benefit rate (CBR) of 40%, with responses confined to patients whose tumors displayed PI3K pathway abnormalities, present in 74% of those evaluated. The existence of these abnormalities was substantially associated with enhanced response (31 vs. 0%, *p* = 0.04), demonstrating that PI3K pathway can function as a predictive biomarker for the efficacy of this regimen. These results demonstrate that there should be more randomized trials with DAT and DAE in metaplastic and mesenchymal TNBC cohorts, especially among those with detectable PI3K pathway modifications [289].

A phase I dose‐escalation study has evaluated the combination of sapanisertib, a potent dual mTORC1/2 inhibitor, with metformin in 30 patients with advanced solid tumors that were resistant or refractory to conventional therapies. Patients were administered sapanisertib (3 or 4 mg daily) in combination with metformin (500–1500 mg daily) after a metformin titration phase. Different tumors demonstrated a significant frequency of alterations in the PI3K/AKT/mTOR pathway, including mutations in PIK3CA (27%), PTEN (17%), AKT1/2 (10%), and mTOR (10%). The disease control rate (comprising partial responses and stable illness) was 63%, with four patients demonstrating partial responses, three of whom possessed PTEN mutations, and two with concomitant mutations affecting TSC or STK11. The predominant adverse events were gastrointestinal symptoms and rash, but grade 3 toxicities, including hyperglycemia and exhaustion, were treatable. The maximum tolerated dosage (MTD) was determined to be 4 mg sapanisertib in combination with 1000 mg metformin. The sapanisertib–metformin combination is tolerated and safe and can provide therapeutic advantages in cancers with PTEN or wider PI3K pathway abnormalities, requiring more studies [290].

A phase I/II study evaluated the safety, pharmacokinetics, and efficacy of the PI3K inhibitor pilaralisib and the dual PI3K/mTOR inhibitor voxtalisib, both in combination with letrozole, in patients with HR+, HER2‐negative metastatic breast cancer resistant to nonsteroidal aromatase inhibitors. In phase I, the maximum tolerated doses were determined to be 400 mg of pilaralisib administered once daily, 50 mg of voxtalisib administered twice daily, and 2.5 mg of letrozole administered daily. Phase II had minimal clinical activity, with a solitary partial response in the pilaralisib cohort and 6‐month progression‐free survival rates of 17% for pilaralisib and 8% for voxtalisib. Both combinations were generally well tolerated, with the most common grade ≥3 adverse events being enhanced in liver enzymes and rash. Pharmacokinetic analyses demonstrated no significant interactions with letrozole, whereas pharmacodynamic evaluations showed a more prominent effect from pilaralisib, especially on glucose metabolism. No association was identified between modifications in the PI3K pathway and therapy response, demonstrating limited efficacy of these combinations in this patient group, despite acceptable safety profiles [291].

A phase I/Ib multicenter study has evaluated the MTD, safety, and pharmacokinetics of BEZ235, an oral dual PI3K/mTOR inhibitor, when used as monotherapy or in combination with trastuzumab in patients with advanced solid tumors, including HER2‐positive advanced breast cancer (aBC). BEZ235 was assessed in various oral formulations including hard gelatin capsules, solid dispersion system (SDS) capsules, and SDS sachets with the SDS sachet eventually identified as the optimal format. In a cohort of 183 recruited patients, the MTD for BEZ235 monotherapy was established at 1200 mg/day for the SDS sachet and 1000 mg/day for SDS capsule A. However, when combined with trastuzumab, the MTD decreased to 600 mg/day due to enhanced gastrointestinal toxicity. The partial responses were significant in 13.3% of patients within the HER2+ aBC group. Prevalent treatment‐related side effects including nausea, diarrhea, and vomiting, significantly occurring with increased frequency at increased dosages. Pharmacokinetic studies demonstrated significant variability at dosages of 100 mg or above [292].

A phase Ib/II TBCRC032 study has investigated the safety and effectiveness of the androgen receptor antagonist enzalutamide in combination with the PI3K inhibitor taselisib in patients with metastatic AR‐positive (≥10%) breast cancer, including TNBC. During the dose‐escalation phase, patients with either ER+ or TNBC were administered enzalutamide (160 mg) in combination with taselisib, and no dose‐limiting toxicities were recognized, therefore providing the regimen's tolerability. In the randomized phase II segment, TNBC patients were administered either enzalutamide monotherapy or in combination with taselisib. The combined treatment provided a CBR of 35.7% and a median progression‐free survival (PFS) of 3.4 months. Patients with luminal AR (LAR) subtype TNBC displayed superior CBRs (75 vs. 12.5%) and extended PFS (4.6 vs. 2.0 months) relative to non‐LAR patients, demonstrating subtype‐specific efficacy. The predominant adverse effects were hyperglycemia and dermatological rash. Genomic profiling recognized FGFR2 fusions and AR splice variants that affecting treatment results [293]. Table 5 is a summary of the clinical trials regarding the PI3K/AKT/mTOR axis in cancers.

**TABLE 5:** The clinical trials regarding PI3K/AKT/mTOR axis.

Cancer	Drug	NCT No	Phase	Remark	References
Pancreatic ductal adenocarcinoma (PDAC)	Abemaciclib (±LY3023414 or galunisertib); SOC: gemcitabine or capecitabine	NCT02981342	Phase II	Abemaciclib‐based therapies did not improve disease control or PFS vs. SOC.	[294]
Hormone receptor‐positive, HER2‐negative advanced breast cancer (with PIK3CA mutation)	Alpelisib + fulvestrant	NCT02437318	Phase III	Effective with improved PFS; common AEs (hyperglycemia, rash, diarrhea) manageable with early intervention and dose adjustment.	[295]
Metastatic breast cancer	Buparlisib + capecitabine	NCT01300962	Phase I	Well‐tolerated combo; observed complete and partial responses; more studies are required to investigate pharmacokinetics and biomarkers.	[296]
HER2‐negative locally advanced/metastatic breast cancer	Buparlisib + paclitaxel	NCT01572727	Phase II/III	No PFS benefit in full or PI3K‐activated populations; trial stopped for futility after phase II.	[297]
Metastatic castration‐resistant prostate cancer (mCRPC)	Abiraterone + prednisone + BEZ235	NCT01717898	Phase I	Poorly tolerated; high toxicity with no observed response; development of BEZ235 in prostate cancer discontinued.	[298]
Advanced solid tumors (endometrial cancer)	LY3023414	NCT01655225	Phase I	Tolerable safety profile; partial response in endometrial cancer with PIK3R1/PTEN mutations; RP2D is 200 mg twice daily.	[299]
Advanced solid tumors (mainly lung cancer)	SAR245409 + erlotinib	NCT00777699	Phase I	Combination was tolerable at reduced doses; stable disease in 37.5% of patients; no major pharmacokinetic interaction.	[300]
Relapsed/refractory lymphoma and multiple myeloma (notably DLBCL)	CUDC‐907	NCT01742988	Phase I	Promising activity in DLBCL (5/9 patients responded); recommended phase 2 dose is 60 mg (5/2 schedule); manageable safety profile.	[301]
Pediatric relapsed/refractory solid tumors	Copanlisib	NCT03458728	Phase I	Model‐informed dosing (28 mg/m^2^) achieved consistent exposures across pediatric age groups; dose aligns with adult pharmacokinetics.	[302]
Advanced endometrial cancer (PI3K pathway‐mutated)	LY3023414	NCT02549989	Phase II	Modest single‐agent activity (ORR 16%) with manageable toxicity; no clear link between mutation type and response.	[303]
Advanced solid tumors (ovarian, breast, endometrial)	Sapanisertib + serabelisib + paclitaxel	NCT03154294	Phase I	Safe and well tolerated; ORR 47%, CBR 73%; strong activity even in platinum‐refractory and heavily pretreated patients.	[304]
Recurrent/metastatic head and neck squamous cell carcinoma (HNSCC)	Copanlisib + cetuximab	NCT02822482	Phase I	Poor tolerability because of hyperglycemia; restricted efficacy; trial was stopped early due to unfavorable toxicity profile.	[305]
ER/PR‐positive breast, ovarian, and endometrial cancers	Anastrozole + everolimus	NCT01197170	–	Well tolerated; clinical benefit in 24% of patients; responses associated with PI3K/AKT/mTOR pathway alterations.	[306]
Advanced solid tumors (thymus, sinonasal, Bartholin's gland cancers)	PQR309	NCT01940133	Phase I	RP2D = 80 mg daily; well tolerated with dose‐proportional PK; partial response and disease control observed, even without PI3K mutations.	[307]
Advanced cancers (notably ER⁺/high‐proliferative breast cancer)	Ridaforolimus + dalotuzumab	NCT00730379	Phase I	Proof‐of‐concept study demonstrated ideal activity, especially in ER⁺/high‐proliferative breast cancer; key DLTs were stomatitis and asthenia.	[308]
Castration‐resistant prostate cancer (CRPC), posttaxane	Ridaforolimus	NCT00110188	Phase II	No objective responses; 47% had stable disease; well tolerated; future experiments consider combo or maintenance strategies.	[309]
Advanced sarcoma (PTEN‐loss UPS, TSC2‐mutant tumors, ER⁺ leiomyosarcoma)	Nab‐sirolimus + nivolumab	NCT05103358	Phase I/II	Safe combo; modest efficacy; best responses in biomarker‐selected patients (PTEN loss, TSC2 mutation); provides biomarker‐based future research.	[310]
HER2‐overexpressing metastatic breast cancer (trastuzumab‐pretreated)	Everolimus + vinorelbine + trastuzumab	NCT00426530	Phase I	Well tolerated; DCR 83%, ORR 19.1%, PFS ∼31 weeks; no drug–drug interaction; supports use posttrastuzumab in heavily pretreated patients.	[311]
Advanced pancreatic adenocarcinoma	Capecitabine + everolimus	NCT01079702	Phase II	Moderately active regimen; DCR 38%, RR 6%; median OS 8.9 months; manageable toxicity profile supports further exploration.	[312]
Advanced cancers with ErbB alterations (breast, colorectal, cervical, endometrial)	Neratinib + everolimus	NCT03065387	Phase I	ORR 19%, CBR 28.6%; manageable toxicity; MTD defined; reduced neratinib clearance noted—supports further evaluation in ErbB‐driven cancers.	[313]
Locally advanced cervical cancer	Everolimus + cisplatin + radiotherapy	NCT01217177	Phase I	MTD established at 5 mg/day; manageable toxicity; preclinical rationale and early data support further clinical investigation.	[314]
Advanced squamous non‐small cell lung cancer (sq‐NSCLC)	Ibrilatazar + paclitaxel + carboplatin	NCT03366480	Phase II	ORR 52% (FA population), PFS 6.2 months, OS up to 22.5 months; well tolerated with promising efficacy; supports further development.	[315]
Metastatic castration‐resistant prostate cancer (mCRPC)	Temsirolimus + cixutumumab	NCT01026623	Phase I	Limited antitumor activity; no PSA responses; high toxicity including pneumonitis and hyperglycemia; trial discontinued.	[316]
Hormone receptor‐positive recurrent or metastatic endometrial cancer	Vistusertib + anastrozole	NCT02730923	Phase I/II	Improved PFS (5.2 vs. 1.9 months) and response rate vs. anastrozole alone; well tolerated; supports use in hormone‐resistant disease.	[317]
Advanced solid tumors	PAMs (PI3K/AKT/mTOR inhibitors), CTAs, ICIs, MTAs	–	Phase I	PAMs and CTAs significantly increase infection risk vs. MTAs; ICIs do not increase infection risk compared with MTAs.	[318]
ER‐positive, HER2‐negative primary breast cancer (esp. luminal B subtype)	Pictilisib + anastrozole	–	Phase II	Combination significantly enhanced tumor proliferation suppression; greatest effect seen in luminal B subtype; no benefit in luminal A.	[319]
RAS‐ or BRAF‐mutant ovarian, NSCLC, and pancreatic cancer	Buparlisib + trametinib	NCT01155453	Phase Ib	Promising activity in KRAS‐mutant ovarian cancer (ORR 29%, PFS 7 mo); limited effect in NSCLC and pancreatic cancer; high toxicity.	[320]
Advanced solid tumors (PIK3CA‐mutant breast and gynecologic cancers)	AZD5363 (capivasertib)	NCT01226316	Phase I	Well tolerated at 480 mg BID (4/7 schedule); modest response rates (<10%) in PIK3CA‐mutant cohorts led to stopping further enrollment.	[321]
Various solid and hematologic tumors (breast cancer)	AZD5363 (capivasertib)	–	Phase I	Potent AKT inhibitor; effective in PIK3CA/PTEN‐mutant and trastuzumab‐resistant HER2+ breast cancers; resistance linked to RAS mutations; supports monotherapy and combination.	[322]
Persistent/recurrent epithelial ovarian cancer/primary peritoneal cancer (EOC/PPC)	Temsirolimus	NCT00429793	Phase II	Modest activity (PFS ≥6 mo in 24%); not sufficient for phase III in unselected patients; cyclin D1 and CTC markers show promise for future stratification.	[323]
Advanced malignancies (notably parotid, metaplastic breast, and endometrial endometrioid cancer)	Temsirolimus + bevacizumab + liposomal doxorubicin	NCT00761644	Phase I	Promising activity (42% DCR); highest responses in tumors with PIK3CA/PTEN aberrations; well tolerated; supports further tumor‐ and biomarker‐specific trials.	[324]
Advanced solid tumors with TSC1/TSC2 or MTOR mutations (histology‐agnostic)	Everolimus	–	Phase II	Low ORR (7%) despite mutation selection; modest PFS/OS; rare responses seen in specific cases (PEComa‐like uterine carcinoma).	[325]
Advanced solid tumors (metastatic melanoma)	Temsirolimus + hydroxychloroquine	–	Phase I	Safe and tolerable combo; no objective responses but high rates of stable disease (67–74%); supports further study of mTOR + autophagy inhibition.	[326]
Advanced cancers (Hodgkin lymphoma, PEComa, HCC)	Sirolimus + vorinostat	NCT01087554	Phase I	Feasible combo; main DLT was thrombocytopenia; RP2D: sirolimus 4 mg + vorinostat 300 mg; early signs of activity in select tumor types.	[327]

## 3 Conclusion and Perspectives

One of the most commonly dysregulated pathways in cancer is PI3K/AKT/mTOR axis with significant contribution to the tumor biology. The abnormal expression of PI3K and downstream targets has been shown to control carcinogenesis, progression, metastasis, and therapeutic resistance. Since cancer is a leading cause of death, the development of novel therapeutics based on targeting PI3K/AKT can significantly improve the prognosis and survival of patients. The present review highlighted the versatile and multifaceted functions of PI3K/AKT/mTOR axis in solid and hematological tumors. This pathway has been demonstrated to exert significant biological functions regulating survival, proliferation, metabolism, angiogenesis, immune evasion, EMT, apoptosis, autophagy, ferroptosis, and glycolysis. Since the function of PI3K is carcinogenic, upregulation of PI3K/AKT/mTOR can reduce apoptosis and ferroptosis, while it increases EMT and angiogenesis. The only challenge is related to the regulation of autophagy in human cancers. Notably, autophagy has dual function exerting protective and cytotoxic impacts. The mutations in the components of PI3K/AKT/mTOR axis such as *PIK3CA*, *PTEN*, and *mTOR* have been demonstrated during initiation, development and progression of cancer. Such mutations are responsible for the aberrant signaling, enhancing carcinogenesis and apoptosis resistance. The upregulation of PI3K/AKT/mTOR axis has been shown to increase resistance to chemotherapy, radiotherapy, and immunotherapy. Its overexpression can increase levels of Bcl‐2, while it downregulates Bax to reduce apoptosis and cause therapy resistance. However, apoptosis is not the only target and PI3K/AKT/mTOR axis can affect other cell death mechanisms including autophagy and ferroptosis in regulating therapeutic resistance. The functional of PI3K/AKT/mTOR axis in the regulation of EMT is vital for affecting the metastasis of tumor cells. Silencing PI3K axis can impair EMT in reducing metastasis. Interestingly, PI3K/AKT/mTOR axis has been demonstrated to regulate both glycolysis and ferroptosis in tumor cells. Such support of glycolysis is vital for increasing the proliferation of cancer cells. The noncoding RNAs as epigenetic factors can regulate PI3K/AKT/mTOR axis in cancers. The phytochemicals, small molecules, and nanoparticles can suppress this axis in cancer inhibition.

The PI3K/AKT/mTOR has been among the most common pathways in cancer therapy due to function in the regulation of growth, metastasis, survival, and therapeutic resistance. The therapeutic importance of PI3K/AKT results from its function in the regulation of EMT, autophagy, and immune evasion along with therapeutic resistance. Therefore, the specific inhibitors of PI3K/AKT should be developed including as small compounds, phytochemicals, and nanoparticle‐based systems, as effective ways to improve treatment efficacy and overcome resistance to chemotherapy, radiation, and immunotherapy. Furthermore, the incorporation of PI3K/AKT/mTOR‐targeted therapies with genomic sequencing facilitates the precision oncology strategies, customizing interventions according to distinct tumor characteristics and enhancing patient outcomes. There should be also a critical view toward the expression of PI3K/AKT in the different stages of cancers, and evaluating its procarcinogenic function.

The future researches and studies should focus on revealing the exact molecular interactions and underlying mechanisms between PI3K/AKT/mTOR and other oncogenic or immune‐regulatory pathways in the TME. There is also a vital need for elucidating the context‐dependent dual functions of autophagy and apoptosis regulated by this axis, enhancing treatment timing and combinations. The participation of noncoding RNAs in regulating the PI3K/AKT/mTOR provides a promising gate for the progression of RNA‐based diagnostics and treatments. The impact of PI3K/AKT on the ferroptosis and metabolism can provide new insights in cancer therapy. Preclinical and clinical investigations into combination therapies and biomarker‐based patient classification are vital for increasing the translational potential of targeting the PI3K/AKT/mTOR in cancer.

Emerging targeting strategies for the PI3K/AKT/mTOR signaling pathway in cancer are pushing beyond traditional small molecule inhibitors toward more sophisticated and selective approaches. PROTACs (Proteolysis Targeting Chimeras) represent a promising modality by harnessing the ubiquitin–proteasome system to selectively degrade key oncogenic proteins in the PI3K/AKT/mTOR axis, potentially overcoming resistance associated with mere enzymatic inhibition. Metamorphic inhibitors, a novel class of compounds, exploit the conformational plasticity of target proteins to induce dysfunctional or inactive structural states, offering the advantage of disrupting noncatalytic functions of proteins like AKT or mTOR that are critical for cancer cell survival and proliferation. Additionally, combination immunotherapy strategies are increasingly being explored to augment the effects of PI3K/AKT/mTOR inhibition, especially in immune‐evasive tumors. By combining pathway inhibitors with checkpoint blockade therapies (anti‐PD‐1/PD‐L1), researchers aim to reverse the immunosuppressive TME often driven by aberrant PI3K signaling. These combinatorial approaches not only enhance tumor immunogenicity but also sensitize tumors to immune‐mediated eradication. Collectively, these innovative strategies hold promise to overcome limitations of conventional therapies, mitigate resistance, and offer more durable responses in PI3K/AKT/mTOR‐driven malignancies.

## Author Contributions

Mingyang Jiang: conceptualization, writing—review editing, and writing—original draft; Ke Zhang: conceptualization, writing—review editing, and writing—original draft; Zhenwang Zhang: visualization and preparation of tables; Xinran Zeng: writing—review editing; Zihang Huang: writing—review editing; Peizhuo Qin: writing—review editing; Zhilin Xie: writing—review editing; Xue Cai: writing—review editing; Milad Ashrafizadeh: conceptualization and writing—review editing; Yu Tian: conceptualization, writing—review editing, and literature collection; Ruqiong Wei: conceptualization, writing—review editing, and literature collection. The final version of manuscript has been read and approved by all authors.

## Ethics Statement

The authors have nothing to report.

## Conflicts of Interest

The authors declare no conflict of interest.

## Data Availability

The authors have nothing to report.
